# One-Pot Synthesis of Alkynyl-Conjugated Phenylalanine Analogues for Peptide-Based Fluorescent Imaging

**DOI:** 10.1021/acs.orglett.5c02361

**Published:** 2025-07-10

**Authors:** Olivia Marshall, Andrew Sutherland

**Affiliations:** School of Chemistry, The Joseph Black Building, 3526 University of Glasgow, Glasgow G12 8QQ, United Kingdom

## Abstract

A series of unnatural amino acids featuring conjugated aryl–alkyne side chains was synthesized from tyrosine via a one-pot hydroxyl group activation and copper-free Sonogashira cross-coupling. This efficient strategy enabled rapid access to modified phenylalanine analogues and the discovery of a fluorescent lead compound with enhanced photophysical properties and sensitivity to lipid-rich environments. Excitation of the alkenyl-conjugated lead analogue in dipeptides, without quenching or interference from intrinsic proteinogenic fluorophores demonstrates its potential for biological imaging applications.

## Graphical abstract

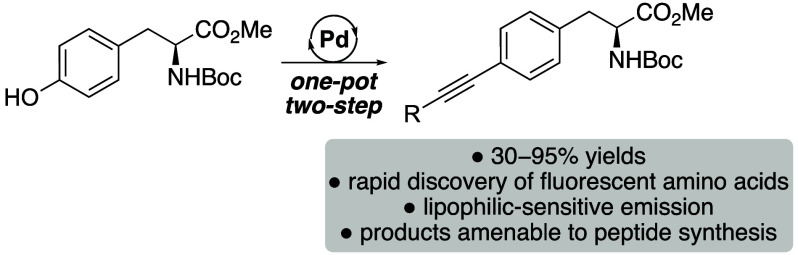

Fluorescence spectroscopy is an important optical technique widely utilized in biological imaging, chemical sensing, and materials science.^1^ The high sensitivity down to single-molecule level and the ability to perform real-time monitoring have resulted in valuable applications of this technique in the understanding of biological processes.^2,3^ However, to investigate biological mechanism and cellular processes requires the development of small molecules with tuned fluorescence. The efficiency and brightness of fluorescent small molecules are linked to their electronic structures, which can be tuned through molecular design. One of the most effective strategies for enhancement of fluorescence is the extension of π-conjugation within the molecule.^4^ Extended conjugation lowers the energy gap between the highest occupied and lowest unoccupied molecular orbitals (HOMO–LUMO), enabling a shift of absorption and emission to longer wavelengths. Additionally, increased conjugation often improves the molar absorptivity and quantum yield by promoting delocalization of excited-state electrons, thereby stabilizing the excited state and reducing nonradiative decay. These effects collectively contribute to stronger, longer-lasting fluorescence, making the design of extended conjugated compounds a key approach for the development of advanced fluorescent probes.

The intrinsic fluorescence of proteinogenic amino acids is limited to tryptophan, tyrosine and phenylalanine, which exhibit relatively modest photophysical properties.^2a^ To create bright fluorogenic amino acids that can be easily incorporated into peptides and proteins for biological imaging, a key tactic has involved extending side chain conjugation.^5^ The introduction of planarity and rigidity within the side chain of amino acids restricts vibrational and rotational degrees of freedom that contribute to nonradiative decay, resulting in fluorophores with enhanced quantum yields. This approach has been widely used to improve the photophysical properties of l-phenylalanine (**1**) (Figure 1a), which has the weakest quantum yield of the natural amino acids (Φ = 0.024).^2a^ For example, the incorporation of a biphenyl motif via a Suzuki-Miyaura cross-coupling reaction generated terphenyl amino acid **2**,^6^ which has a quantum yield of 0.83 ^7^ and was incorporated into dihydrofolate reductase to measure conformational change of the protein during inhibitor binding.^8^ The fluorescence of phenylalanine has also been enhanced by the incorporation of fluorophores. The Ackermann and Vendrell groups synthesized a library of phenylalanine-BODIPY conjugates via Pd-catalyzed C­(sp^3^)–H activation and showed that compounds such as **3**, which display 2 orders of magnitude higher quantum yields in lipophilic environments, could be used to detect *Candida* strains in urine samples.^9^

**Figure 1:**
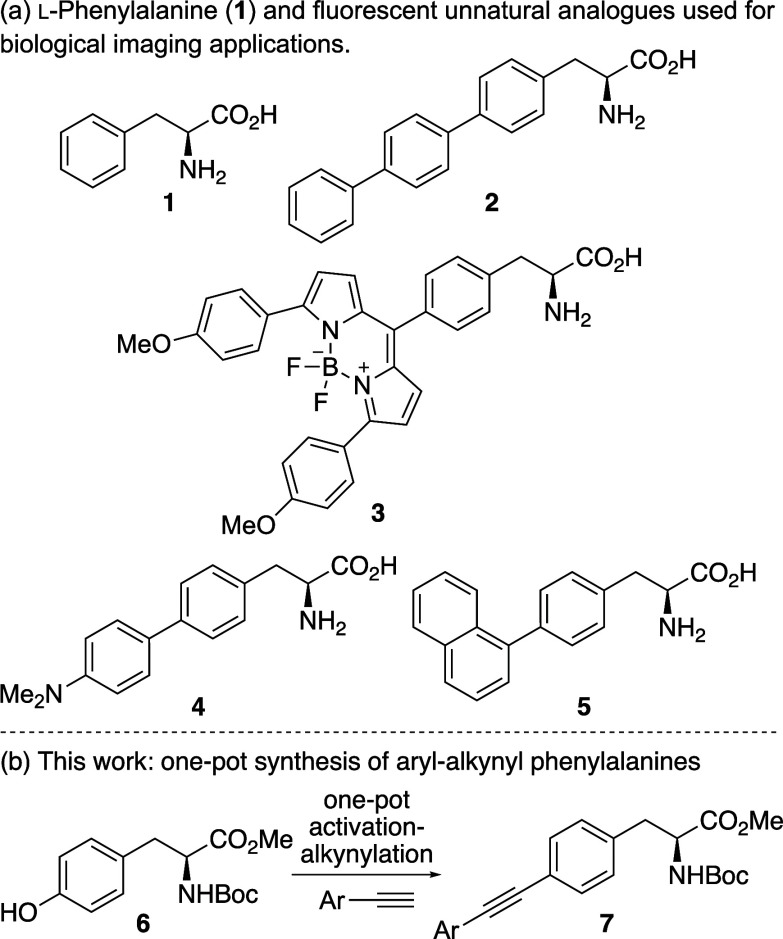
(a) l-Phe and fluorescent analogues. (b) This work.

Recently, we reported the preparation of aryl-substituted phenylalanine analogues from tyrosine using a one-pot, two-step approach involving a Suzuki-Miyaura cross-coupling reaction.^7^ This one-pot approach allowed the rapid discovery of novel fluorescent amino acids such as dimethylaminobiphenyl analogue **4** (Figure 1), which displayed bright charge transfer-based fluorescence (Φ = 0.73) and could be used for monitoring protease activity via Förster resonance energy transfer (FRET) experiments. To enhance the brightness of biaryl amino acids, we proposed that the insertion of an alkyne between the aryl groups would generate compounds with improved photophysical properties. While the stretching of fluorophores using alkynes has been reported with other molecular structures,^2–4^ this type of modification is rare for the development of fluorescent unnatural amino acids. The two reported examples utilize multistep routes to access amino acids with either benzoxazole or benzotriazole side chains, which are then subjected to Sonogashira reactions generating compounds with improved photophysical properties.^10,11^ To access alkyne-enhanced fluorescent amino acids more quickly and efficiently, we believed this could be achieved via a one-pot process involving activation and a Sonogashira cross-coupling reaction of tyrosine. We now report the one-pot synthesis and photophysical properties of a series of alkynyl-conjugated phenylalanine analogues (Figure 1b). As well as demonstrating the sensitivity of the lead amino acid for lipid-rich environments, we also show that this 1-naphthyl analogue can be incorporated into dipeptides and excited in the presence of fluorescent proteinogenic amino acids.

Initially, a brief study was conducted for the optimization of the one-pot hydroxyl activation and Sonogashira cross-coupling reaction with tyrosine derivative **6** (Table 1). An aryl nonaflate (ArONf),^12^ an intermediate increasingly used for cross-coupling reactions was selected as the activated species and readily prepared by reaction of **6** with perfluoro-1-butanesulfonyl fluoride in the presence of cesium carbonate. On completion of this step, a Sonogashira cross-coupling reaction with phenylacetylene was investigated.^13^ The first attempt investigated a separate cross-coupling reaction using a palladium and copper cocatalytic system, triethylamine as the base and with initial activation at 90 °C (entry 1). However, this produced no product. Akai and co-workers previously reported the one-pot activation and alkynylation of simple phenols using PdCl_2_(MeCN)_2_ and XPhos for the Sonogashira reaction.^14^ Use of this catalytic system with tyrosine **6** as a one-pot, two-step process required a reaction time of 21 h for the cross-coupling step and gave **7a** in 51% yield (entry 2). During our one-pot synthesis of biaryl amino acids using a Suzuki-Miyaura reaction,^7^ we found that the use of the Buchwald precatalyst XPhos Pd G2 was highly effective.^15^ The use of XPhos Pd G2 (5 mol %) in a single batch addition to the nonaflate intermediate resulted in a fast Sonogashira reaction at 70 °C (3.5 h), which gave **7a** in 88% yield (entry 3).

**Table 1:**
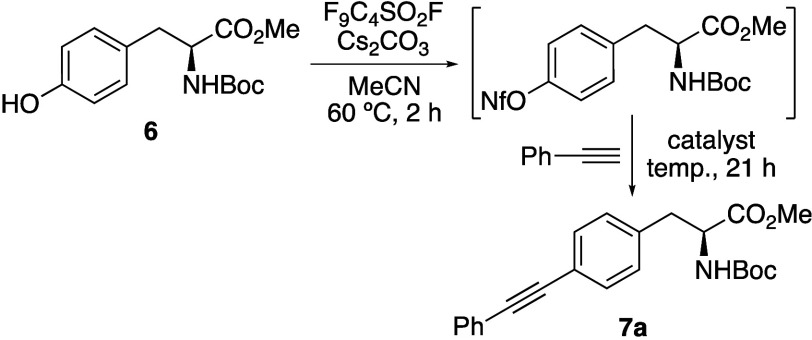
Optimization of the One-Pot Alkynylation of Tyrosine Derivative **6**

entry	catalyst system	loading (mol %)	temp (°C)	yield (%)^a^
1^b^	Pd(PPh_3_)_2_Cl_2_/CuI	10 + 20	90 to rt	0
2	PdCl_2_(MeCN)_2_/XPhos	1 + 2	60	51
3^c^	XPhos Pd G2	5	70	88

a Isolated yields.

b Nonaflate was isolated and Et_3_N/DMF was used during the 2nd step.

c Sonogashira reaction was left for 3.5 h.

Following optimization of the one-pot process, the scope was explored with a range of arylacetylenes with various electronics that would allow full examination of the photophysical properties of the resulting amino acids (Scheme 1).^16^ Under the optimized one-pot conditions, a range of phenylacetylenes and naphthylacetylenes reacted rapidly and cleanly with tyrosine **6** to give the corresponding adducts (**7a**–**7g**) in moderate to excellent yields (51–95%). The synthesis of a heteroaryl example using 2-ethynylpyridine was more challenging. This required a higher temperature (85 °C) to promote the Sonogashira reaction and gave **7h** in 30% yield. Although the primary aim of this study was to develop conjugated phenylalanine analogues for fluorescent imaging, it was proposed that the one-pot process could be used to access other important amino acid building blocks. To explore this, the one-pot coupling of tyrosine **6** with TMS-acetylene was investigated. However, application of the optimized procedure led to product mixtures due to carbonate-mediated removal of the TMS-group. Substituting the base with Et_3_N enabled a clean one-pot transformation. Further optimization revealed that batch addition of catalyst (2 × 3 mol %) over a 23 h reaction period resulted in complete conversion and the quantitative isolation of **7i**. This demonstrates that this one-pot method can be effectively extended to prepare other alkyne-functionalized analogues, broadening its utility for applications such as click chemistry.

**Scheme 1:**
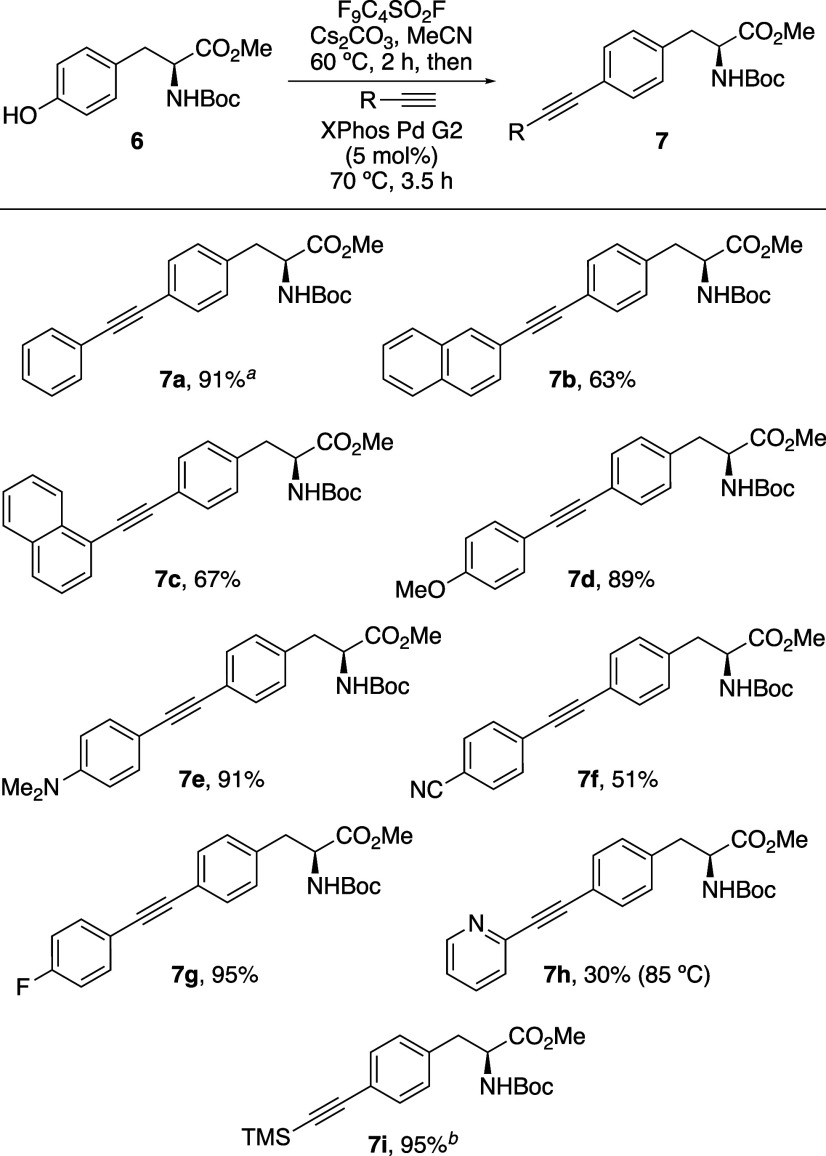
Synthesis of Alkynyl α-Amino Acids **7a**–**i**

a 1 mmol scale reaction.
b Prepared using Et_3_N, batch addition of catalyst (2 × 3 mol %) and with a reaction time of 23 h.

The photophysical properties of arylalkynyl amino acids **7a**–**h** were measured and found to exhibit red-shifted absorption and emission spectra compared to parent compound, l-phenylalanine (**1**).^1,17^ Among these, the most fluorescent compounds, naphthyl analogues **7b** and **7c**, as well as alkynylphenylalanines with conjugated electron-withdrawing substituents (**7f** and **7h**), were selected for further analysis (Table 2). Notably, 2-naphthyl analogue **7b** and 4-cyanophenyl analogue **7f** demonstrated strong fluorescence with good quantum yields and brightness. However, 1-naphthyl analogue **7c**, which exhibited mirrored vibrational bands in both its absorption and emission spectra (Figure 2a), showed the highest quantum yield of 0.65, resulting in a brightness of 15,550 cm^–1^ M^–1^.

**Table 2:** Photophysical Data of Selected α-Amino Acids^17^

amino acid	λ_Abs_ (nm)^a^	ε (cm^–1^ M^–1^)	λ_Em_ (nm)^a^	Φ_F_ ^b^	brightness (cm^–1^ M^–1^)
**1**	258	200	282	0.024	5
**7b**	274	39200	341	0.23	8950
**7c**	316, 336	24100	343, 359	0.65	15550
**7f**	302	34400	356	0.21	7340
**7h**	296	20400	334	0.06	1270
**8**	316, 346	25300	342, 362	0.57	14480
**5** ^7^	282	10300	342	0.27	2800

a Spectra were recorded at 5 μM in MeOH.

b Quantum yields (Φ_F_) were determined using l-Trp and anthracene as standards.

**Figure 2:**
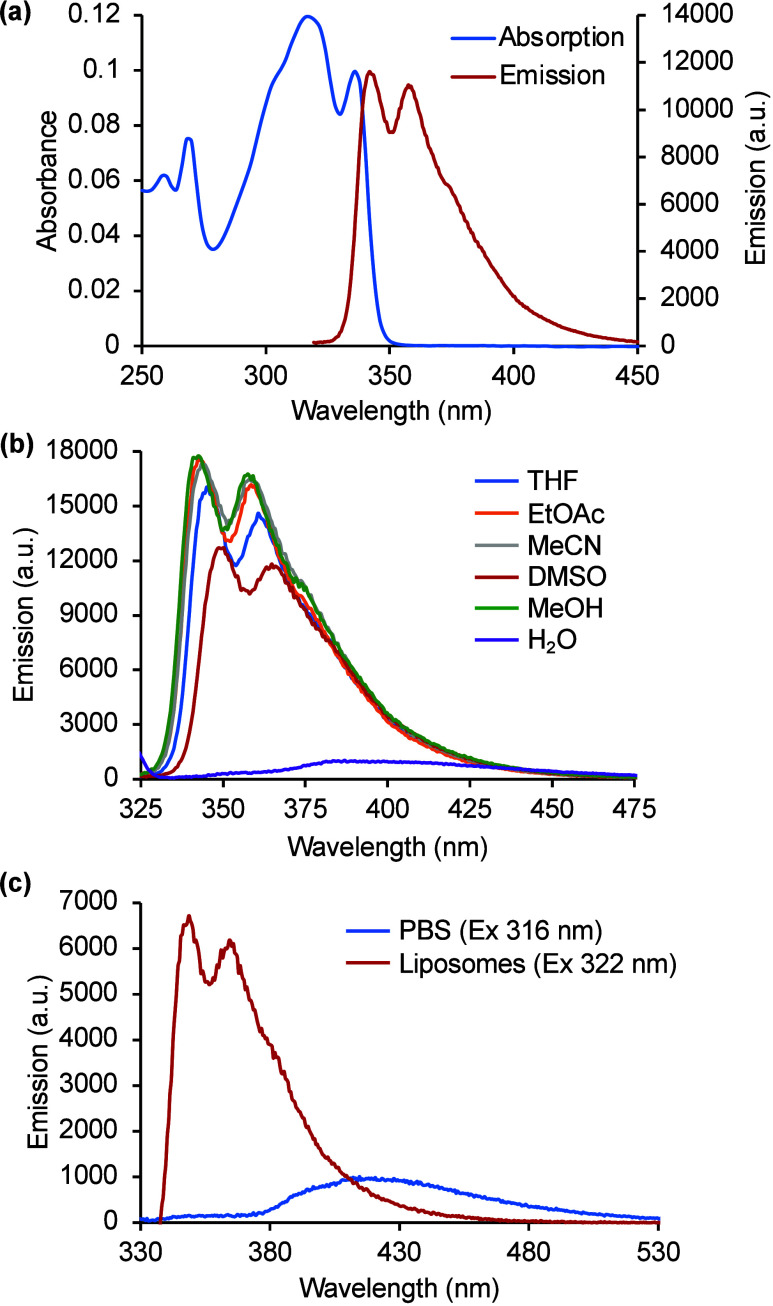
(a) Absorption and emission spectra of **7c** (5 μM in MeOH). (b) Solvatochromic study of **7c** (5 μM). (c) Emission spectra of **7c** in PBS vs liposomes (PC/cholesterol, 7:1) (5 μM).

On identification of 1-naphthyl analogue **7c** as the lead compound, an in-depth study of its photophysical properties was conducted. A viscosity-dependent experiment, using various concentrations of ethylene glycol in methanol revealed minimal changes in both absorption and emission spectra, suggesting a rigid conformation in both the ground and excited states.^17^ An aggregation study showed that fluorescence intensity peaked at 80 μM, while aggregation induced quenching was only observed at concentrations (350 μM) much higher than would be used for biological imaging.^3,5^ The most interesting results for **7c** emerged from a solvatochromic study. In the absorption and emission spectra, negligible changes were observed using various organic solvents (Figure 2b). However, in water, **7c** displayed a bathochromic shift of emission to 390 nm and significantly reduced fluorescence intensity. Lipophilic sensitive fluorophores are important for biological imaging as they can selectively integrate into lipid-rich environments like cell membranes, enabling precise visualization of membrane dynamics and organization.^18^ Based on the fluorescence behavior of **7c** in organic solvents versus water, and its linear structure that may enable insertion into lipid bilayers, it was proposed that this unnatural amino acid could serve as a lipophilic-sensitive probe. To evaluate this potential, the fluorescence emission of **7c** in phosphate-buffered saline (PBS) was compared to its emission in phospholipid bilayer membranes designed to mimic biological environments (Figure 2c). In PBS, **7c** exhibited weak fluorescence, likely due to micelle or aggregate formation, which can lead to quenching. In contrast, **7c** showed strong fluorescence in liposomes (phosphatidylcholine/cholesterol, 7:1),^19^ with an 8.5-fold increase in intensity. These results indicate that upon accumulation within lipid environments, **7c** adopts a membrane-bound form that is highly emissive.

To evaluate the effect of alkyne insertion, 1-naphthyl analogue **7c** was deprotected (Figure 3a), to allow direct comparison with the corresponding biaryl amino acid **5** (Figure 1a).^7^ Deprotection was achieved by cesium carbonate-mediated ester hydrolysis, followed by acid removal of the Boc-protecting group, affording amino acid **8** in 64% yield over the two steps. Photophysical analysis revealed that amino acid **8** retained the enhanced properties observed for protected derivative **7c**. Comparison of the alkyne-extended fluorophore **8** with the truncated biphenyl analogue **5** confirms the advantages of this structural modification (Table 2). Amino acid **8** exhibits red-shifted absorption, increased molar absorption coefficient and improved quantum yield, resulting in a 5-fold enhancement in brightness.

**Figure 3:**
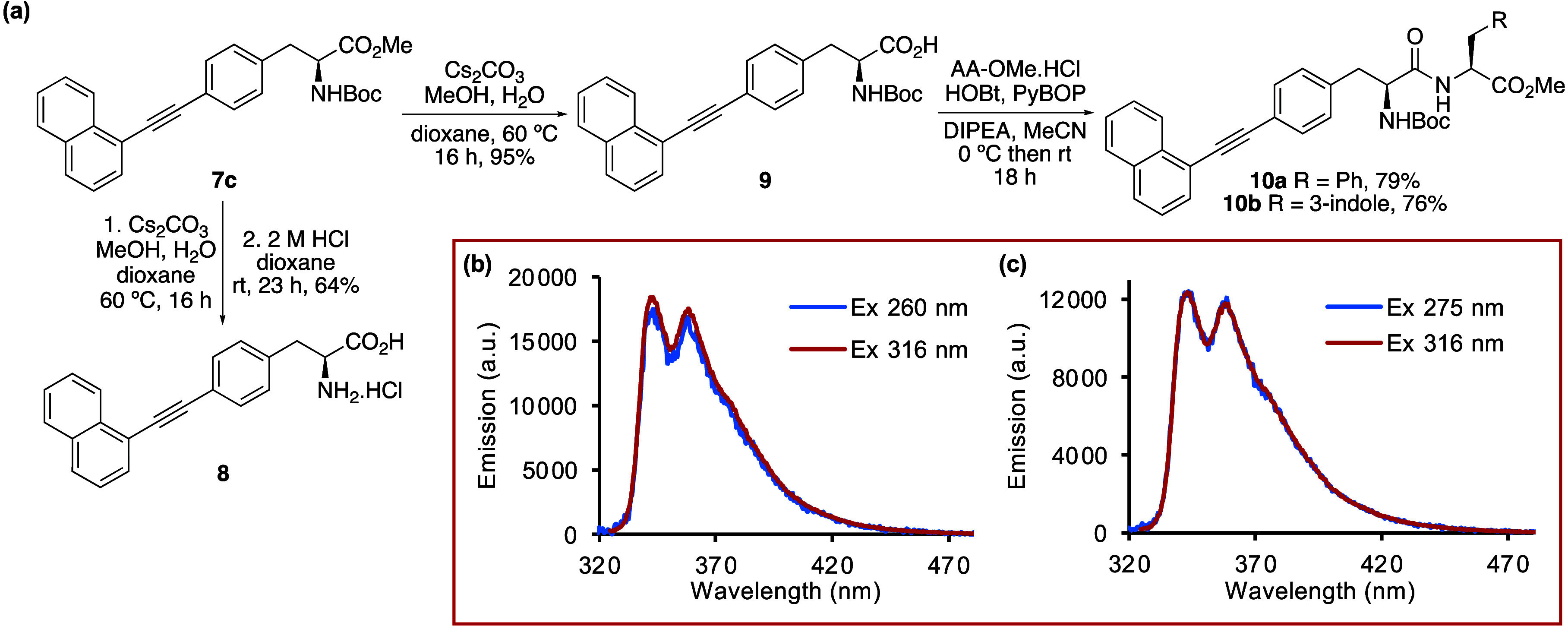
(a) Synthesis of deprotected amino acid **8** and dipeptides **10a** and **10b**. (b) Emission spectra of **10a** (5 μM in MeOH). (c) Emission spectra of **10b** (5 μM in MeOH).

To demonstrate the utility of alkyne-extended phenylalanines for peptide synthesis and their ability to undergo excitation in the presence of naturally fluorescent amino acids, two dipeptides were prepared (Figure 3a). After ester hydrolysis of **7c**, the resulting carboxylic acid **9** was coupled with the methyl esters of l-phenylalanine and l-tryptophan using HOBt and PyBOP as the coupling reagents. This gave dipeptides **10a** and **10b** in 79% and 76%, respectively. Each dipeptide was excited at the wavelength of the proteinogenic amino acid (**10a**: 260 nm; **10b**: 275 nm) and that of the 1-naphthylalkyne (316 nm), producing clear, well-defined emission spectra (Figure 3b,c), consistent with those of compounds **7c** and **8**. In addition to demonstrating the compatibility of alkyne-extended phenylalanines for peptides synthesis, these results show that such analogues can be combined with structurally related phenylalanine and the natural amino acid with the strongest fluorescence, tryptophan, without quenching or interference from intrinsic fluorescence.

In summary, arylalkyne-extended phenylalanines were designed as novel fluorophores for peptide-based imaging, using the extended conjugation provided by an alkyne unit to improve photophysical properties. A one-pot synthesis protocol was developed to generate a small library of these compounds from a readily available tyrosine derivative, using nonaflate activation followed by a copper-free Sonogashira reaction. Employing the Buchwald precatalyst, XPhos Pd G2 enabled the rapid synthesis of analogues with diverse electronic characteristics. Photophysical analysis identified 1-naphthyl analogue **7c** as the lead compound, exhibiting red-shifted absorption and a high quantum yield. Comparison with its biaryl analogue demonstrated the value of added alkyne conjugation, with a 5-fold increase in brightness. Further evaluation of 1-naphthyl analogue **7c** highlighted its potential as a probe for lipid-rich biological environments and its compatibility in peptides without interference from endogenous proteinogenic fluorophores. Current work is focused on the application of these arylalkyne-extended phenylalanines for solid phase peptide synthesis and subsequent applications as biological imaging probes.

## Supplementary Material



## Data Availability

The data underlying this study are available in the published article and its Supporting Information.

## Notes

Experimental procedures, characterization data, photophysical data, and NMR spectra of all compounds (PDF)

## References

[1] Lakowicz, J. R. Principles of Fluorescence Spectroscopy, 3rd ed.; Springer, New York, 2006.

[2] Sinkeldam R. W., Greco N. J., Tor Y. (2010). Fluorescent Analogs
of Biomolecular Building Blocks: Design, Properties and Applications. Chem. Rev..

[3] Vendrell M., Zhai D., Er J. C., Chang Y.-T. (2012). Combinatorial
Strategies in Fluorescent Probe Development. Chem. Rev..

[4] Crawford H., Dimitriadi M., Bassin J., Cook M. T., Abelha T. F., Calvo-Castro J. (2022). Mitochondrial Targeting and Imaging
with Small Organic Conjugated Fluorophores: A Review. Chem. Eur. J..

[5] Krueger A. T., Imperiali B. (2013). Fluorescent Amino Acids: Modular
Building Blocks for the Assembly of New Tools for Chemical Biology. ChemBioChem..

[6] Papst S., Noisier A. F. M., Brimble M. A., Yang Y., Krissansen G. W. (2012). Synthesis
and Biological Evaluation of Tyrosine Modified Analogues of the α4β7
Integrin Inhibitor Biotin-R_8_ERY. Bioorg. Med. Chem..

[7] Marshall O., McGrory R., Songsri S., Thomson A. R., Sutherland A. (2025). Expedient
Discovery of Fluorogenic Amino Acid-Based Probes *via* One-Pot Palladium-Catalyzed Arylation of Tyrosine. Chem. Sci..

[8] Chen S., Fahmi N. E., Wang L., Bhattacharya C., Benkovic S. J., Hecht S. M. (2013). Detection of Dihydrofolate Reductase
Conformational Change by FRET Using Two Fluorescent Amino Acids. J. Am. Chem. Soc..

[9] Wang W., Lorion M. M., Martinazzoli O., Ackermann L. (2018). BODIPY Peptide Labeling by Late-Stage C­(sp^3^)–H Activation. Angew. Chem., Int. Ed..

[10] Guzow K., Szabelski M., Malicka J., Karolczak J., Wiczk W. (2002). Synthesis and Photophysical
Properties of 3-[2-(Pyridyl)­benzoxazole-5-yl]-L-alanine Derivatives. Tetrahedron.

[11] Riley L. M., Mclay T. N., Sutherland A. (2023). Synthesis
and Fluorescent Properties
of Alkynyl- and Alkenyl-Fused Benzotriazole-Derived α-Amino
Acids. J. Org. Chem..

[12] Rottländer M., Knochel P. (1998). Palladium-Catalyzed
Cross-Coupling
Reactions with Aryl Nonaflates: A Practical Alternative to Aryl Triflates. J. Org. Chem..

[13] Sonogashira K. (2002). Development
of Pd-Cu Catalyzed Cross-Coupling of Terminal Acetylenes with sp^2^-Carbon Halides. J. Organomet. Chem..

[14] Ikawa T., Saito K., Akai S. (2012). Palladium-Catalyzed
One-Pot Cross-Coupling
of Phenols Using Nonafluorobutanesulfonyl Fluoride. Synlett.

[15] Kinzel T., Zhang Y., Buchwald S. L. (2010). A New Palladium Precatalyst Allows
for the Fast Suzuki-Miyaura Coupling Reactions of Unstable Polyfluorophenyl
and 2-Heteroaryl Boronic Acids. J. Am. Chem.
Soc..

[16] Doan N.-D., de Molliens M. P., Létourneau M., Fournier A., Chatenet D. (2015). Optimization of On-Resin
Palladium-Catalyzed Sonogashira Cross-Coupling Reaction for Peptides
and its Use in a Structure-Activity Relationship Study of a Class
B GPCR Ligand. Eur. J. Med. Chem..

[17] See Supporting Information for all photophysical measurements, data, and individual spectra.

[18] Gamage R. S., Chasteen J. L., Smith B. D. (2023). Lipophilic
Anchors that Embed Bioconjugates in Bilayer Membranes: A Review. Bioconjugate Chem..

[19] Mendive-Tapia L., Zhao C., Akram A. R., Preciado S., Albericio F., Lee M., Serrels A., Kielland N., Read N. D., Lavilla R., Vendrell M. (2016). Spacer-Free BODIPY Fluorogens in Antimicrobial Peptides
for Direct Imaging of Fungal Infection in Human Tissue. Nat. Commun..

