# Supplementary material for: One-Pot Synthesis of Alkynyl-Conjugated Phenylalanine Analogues for Peptide-Based Fluorescent Imaging
Source: Org Lett. 2025 Jul 10;27(29):8023–7. doi: 10.1021/acs.orglett.5c02361 (PMC12305640; doi:10.1021/acs.orglett.5c02361)

Supporting Information for:

One-Pot Synthesis of Alkynyl-Conjugated Phenylalanine Analogues for Peptide-Based Fluorescent Imaging

*Olivia Marshall and Andrew Sutherland**

School of Chemistry, The Joseph Black Building, University of Glasgow, Glasgow G12 8QQ,

United Kingdom.

Table of Contents

1. General Experimental	S2–S3
2. Experimental Procedures and Spectroscopic Data for all Compounds	S3–S12
3. Photophysical Data for all Compounds	S13–S23
4. References	S24
5. ^{1}H and ^{13}C NMR Spectra for all Compounds	S25–S50

1. General Experimental

All reagents and starting materials were obtained from commercial sources and used as received. Reactions were performed open to air unless otherwise mentioned. All reactions performed at elevated temperatures were heated using an oil bath. Brine refers to a saturated aqueous solution of sodium chloride. Flash column chromatography was performed using silica gel 60 (40–63 μm). Aluminium-backed plates pre-coated with silica gel $60F_{254}$ were used for thin layer chromatography and were visualized with a UV lamp or by staining with potassium permanganate, vanillin or ninhydrin. ^{1}H NMR spectra were recorded on a NMR spectrometer at either 400 or 500 MHz and data are reported as follows: chemical shift in ppm relative to the solvent as internal standard ($CHCl_3$, δ 7.26 ppm; CH_3OH, δ 3.31 ppm; DMSO, δ 2.50), multiplicity (s = singlet, d = doublet, t = triplet, q = quartet, m = multiplet or overlap of non-equivalent resonances, integration). ^{13}C NMR spectra were recorded on a NMR spectrometer at either 101 or 126 MHz and data are reported as follows: chemical shift in ppm relative to tetramethylsilane or the solvent as internal standard ($CDCl_3$, δ 77.2 ppm; CD_3OD, δ 49.0 ppm; DMSO-d_6, δ 39.5). Infrared spectra were recorded on a FTIR spectrometer; wavenumbers are indicated in cm^{-1}. Mass spectra were recorded using electrospray techniques. HRMS spectra were recorded using quadrupole time of flight (Q-TOF) mass spectrometers. Melting points are uncorrected. Optical rotations were determined as solutions irradiating with the sodium D line (λ = 589 nm) using a polarimeter. $[\alpha]_D$ values are given in units 10^{-1} deg cm^{-1} g^{-1}. UV-Vis and fluorescence spectra were recorded on a fluorescence and absorbance spectrometer. Absorbance spectra were recorded with an integration time of 0.05 s and a band pass of 5 nm. Emission spectra were recorded with excitation and emission band pass of 5 nm, an integration time of either 1.0 or 0.1 s, and with detector accumulations set to 1. Quantum yields were determined using a comparative method against two standards.[1,2] Anthracene (Φ = 0.27, in ethanol) and L-tryptophan (Φ = 0.14 in methanol) were used as standard references. The integrated fluorescence intensity of each compound was determined from the emission spectra given. Measurements were performed at six different concentrations. Concentrations were chosen to ensure the absorption value was below 0.1 to avoid re-absorption effects. Integrated fluorescence intensity was plotted as a function of the measured absorbance and a linear fit was calculated. The resultant gradient was then used to calculate the quantum yield, using the equation below:

$$\Phi_X = \Phi_{ST}\left(\frac{Grad_X}{Grad_{ST}}\right)\left(\frac{\eta_X^2}{\eta_{ST}^2}\right)$$

Subscript ST signifies the quantities associated with the quantum yield standard. Subscript X signifies the quantities associated with the novel compound. GradX is the determined gradient associated with the novel compound. GradST is the determined gradient associated with quantum yield standard. η is

the refractive index of the solvent used in the fluorescence measurements: 1.361 for ethanol and 1.331 for methanol.

2. Experimental Procedures and Spectroscopic Data for all Compounds

General Procedure for One-Pot Nonaflate Formation and Sonogashira Cross-Coupling Reaction

A solution of methyl (2*S*)-2-(*tert*-butoxycarbonylamino)-3-(4-hydroxyphenyl)propanoate (1 equiv.) and caesium carbonate (3 equiv.) in dry acetonitrile (3 mL per mmol) was degassed under argon for 0.2 h. To this was added perfluoro-1-butanesulfonyl fluoride (1.5 equiv.). The reaction mixture was heated to 60 °C and stirred for 2 h. To this was added acetylene (1.5 equiv.), XPhos Pd G2 (5 mol%). The reaction mixture was stirred at 70 °C for 3.5 h. After cooling to room temperature, the reaction mixture was diluted in ethyl acetate (30 mL) and washed with water (3 × 30 mL). The combined organic layer was dried ($MgSO_4$), filtered and concentrated *in vacuo*. Purification by flash column chromatography gave the arylacetylene-substituted amino acids.

Methyl (2*S*)-2-(*tert*-butoxycarbonylamino)-3-[4'-(phenylethynyl)phenyl]propanoate (7a)

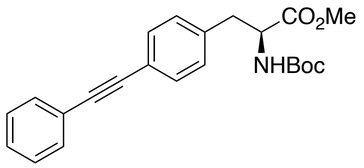

Methyl (2*S*)-2-(*tert*-butoxycarbonylamino)-3-[4'-(phenylethynyl)phenyl]propanoate (**7a**) was synthesized as described in the general procedure using methyl (2*S*)-2-(*tert*-butoxycarbonylamino)-3-(4-hydroxyphenyl)propanoate (**6**) (0.140 g, 0.474 mmol), caesium carbonate (0.460 g, 1.41 mmol), perfluoro-1-butanesulfonyl fluoride (0.130 mL, 0.710 mmol), phenylacetylene (0.160 mL, 1.41 mmol) and XPhos Pd G2 (0.0180 g, 0.0230 mmol) in acetonitrile (1.5 mL). Purification by flash column chromatography, eluting with 20% ethyl acetate in hexane gave methyl (2*S*)-2-(*tert*-butoxycarbonylamino)-3-[4'-(phenylethynyl)phenyl]propanoate (**7a**) (0.156 g, 88%) as a brown solid. Mp 37–39 °C; IR (neat) 3354, 2977, 2363, 1749, 1713, 1509, 1365, 1164 cm^{-1}; $[\alpha]_D^{18}$ +80.7 (*c* 0.1, $CHCl_3$); ^1H NMR (400 MHz, $CDCl_3$) δ 7.54–7.49 (m, 2H), 7.46 (d, J = 7.8 Hz, 2H), 7.40–7.29 (m, 3H), 7.12 (d, J = 7.8 Hz, 2H), 4.99 (d, J = 8.2 Hz, 1H), 4.63–4.56 (m, 1H), 3.72 (s, 3H), 3.14 (dd, J = 14.0, 6.0 Hz, 1H), 3.06 (dd, J = 14.0, 6.4 Hz, 1H), 1.43 (s, 9H); $^{13}C\{^1H\}$ NMR (101 MHz, $CDCl_3$) δ 172.3, 155.2, 136.5, 131.9, 131.7, 129.5, 128.5, 128.4, 123.4, 122.1, 89.7, 89.2, 80.2, 54.5, 52.4, 38.5, 28.4; HRMS (APCI-TOF) *m/z*: $[(MH - CO_2{}^tBu) + H]^+$ Calcd for $C_{18}H_{17}NO_2H$ 280.1332; Found 280.1339.

Methyl (2*S*)-2-(*tert*-butoxycarbonylamino)-3-[4'-(phenylethynyl)phenyl]propanoate (7a): 1 mmol Scale Reaction

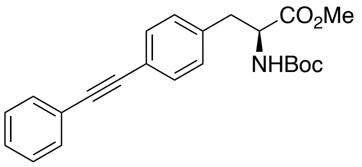

Methyl (2*S*)-2-(*tert*-butoxycarbonylamino)-3-(4-hydroxyphenyl)propanoate (0.295 g, 1.00 mmol) and caesium carbonate (0.977 g, 3.00 mmol) were suspended in dry acetonitrile (4.4 mL) and degassed for 0.2 h. Perfluoro-1-butanesulfonyl fluoride (0.270 mL, 1.50 mmol) was added. The reaction vial was sealed and the reaction mixture was stirred at 60 °C for 2 h. Phenylacetylene (0.330 mL, 3.00 mmol) and XPhos Pd G2 (0.0390 g, 0.0500 mmol) were added and the solution degassed again for 0.1 h. The reaction vial was sealed and the reaction mixture was stirred at 70 °C for 3.5 h. After cooling to room temperature, the reaction mixture was diluted in ethyl acetate (30 mL) and washed with water (3 × 30 mL). The combined organic layer was dried ($MgSO_4$), filtered and concentrated *in vacuo*. Purification by flash column chromatography, eluting with 10–20% ethyl acetate in hexane gave methyl (2*S*)-2-(*tert*-butoxycarbonylamino)-3-[4'-(phenylethynyl)phenyl]propanoate (**7a**) as a brown solid (0.343 g, 91%). Spectroscopic data as described above.

Methyl (2*S*)-2-(*tert*-butoxycarbonylamino)-3-[4'-(2''-ethynylnaphthalene)phenyl]propanoate (7b)

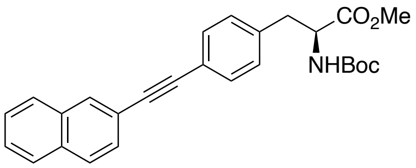

Methyl (2*S*)-2-(*tert*-butoxycarbonylamino)-3-[4'-(2''-ethynylnaphthalene)phenyl]propanoate (**7b**) was synthesized as described in the general procedure using methyl (2*S*)-2-(*tert*-butoxycarbonylamino)-3-(4-hydroxyphenyl)propanoate (**6**) (0.149 g, 0.505 mmol), caesium carbonate (0.495 g, 1.52 mmol), perfluoro-1-butanesulfonyl fluoride (0.140 mL, 0.762 mmol), 2-ethynylnaphthalene (0.229 g, 1.50 mmol) and XPhos Pd G2 (0.0200 g, 0.0254 mmol) in acetonitrile (2.5 mL). Purification by flash column chromatography, eluting with 20% ethyl acetate in hexane followed by flash column chromatography eluting in 50–100% dichloromethane in hexane gave methyl (2*S*)-2-(*tert*-butoxycarbonylamino)-3-[4'-(2''-ethynylnaphthalene)phenyl]propanoate (**7b**) (0.137 g, 63%) as a brown solid. Mp 104–107 °C; IR (neat) 3361, 2980, 2359, 1736, 1686, 1509, 1247, 1163, 1012, 818 cm^{-1}; $[\alpha]_D^{18}$ +60.0 (*c* 0.1, $CHCl_3$); ^1H NMR (400 MHz, $CDCl_3$) δ 8.05 (br s, 1H), 7.87–7.77 (m, 3H), 7.57 (dd, *J* = 8.6, 1.6 Hz, 1H), 7.53–7.46 (m, 4H), 7.14 (d, *J* = 8.0 Hz, 2H), 5.00 (d, *J* = 8.4 Hz), 4.66–4.56 (m, 1H), 3.73 (s, 3H), 3.15 (dd, *J* = 13.8, 5.6 Hz, 1H), 3.07 (dd, *J* = 13.8, 6.2 Hz, 1H), 1.44 (s, 9H); $^{13}C\{^1H\}$ NMR (101 MHz, $CDCl_3$)

δ 172.3, 155.2, 136.6, 133.2, 132.9, 131.9, 131.6, 129.5, 128.5, 128.2, 127.9, 126.8, 126.7, 122.2, 120.7, 90.1, 89.6, 80.2, 54.5, 52.4, 38.5, 28.4; HRMS (ESI-TOF) *m/z*: $[(MH - CO_2{}^tBu) + H]^+$ Calcd for $C_{22}H_{19}NO_2H$ 330.1489; Found 330.1488.

Methyl (2*S*)-2-(*tert*-butoxycarbonylamino)-3-[4'-(1''-ethynylnaphthalene)phenyl]propanoate (7c)

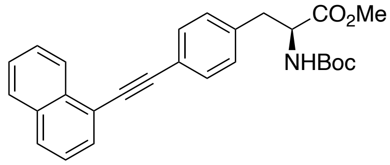

Methyl (2*S*)-2-(*tert*-butoxycarbonylamino)-3-[4'-(1''-ethynylnaphthalene)phenyl]propanoate (**7c**) was synthesized as described in the general procedure using methyl (2*S*)-2-(*tert*-butoxycarbonylamino)-3-(4-hydroxyphenyl)propanoate (**6**) (0.200 g, 0.679 mmol), caesium carbonate (0.655 g, 2.01 mmol), perfluoro-1-butanesulfonyl fluoride (0.180 mL, 1.02 mmol), 1-ethynylnaphthalene (0.290 mL, 2.01 mmol) and XPhos Pd G2 (0.0260 g, 0.0330 mmol) in acetonitrile (3.0 mL). Purification by flash column chromatography, eluting in 20% diethyl ether in hexane followed by a second purification by flash column chromatography eluting in 5% ethyl acetate in toluene gave methyl (2*S*)-2-(*tert*-butoxycarbonylamino)-3-[4'-(1''-ethynylnaphthalene)phenyl]propanoate (**7c**) (0.198 g, 67%) as a yellow oil. IR (neat) 3360, 2977, 1743, 1710, 1504, 1364, 1162, 800, 774 cm^{-1}; $[\alpha]_D^{25}$ +62.3 (*c* 0.1, $CHCl_3$); ^{1}H NMR (400 MHz, $CDCl_3$) δ 8.44 (d, J = 8.0 Hz, 1H), 7.89–7.82 (m, 2H), 7.76 (dd, J = 7.2, 1.2 Hz, 1H), 7.63–7.50 (m, 4H), 7.46 (dd, J = 8.0, 7.2 Hz, 1H), 7.17 (d, J = 8.0 Hz, 2H), 5.03 (d, J = 8.4 Hz, 1H), 4.70–4.55 (m, 1H), 3.74 (s, 3H), 3.18 (dd, J = 13.6, 5.8 Hz, 1H), 3.09 (dd, J = 13.6, 6.0 Hz, 1H), 1.45 (s, 9H); $^{13}C\{^1H\}$ NMR (101 MHz, $CDCl_3$) δ 172.3, 155.2, 136.7, 133.4, 133.3, 131.9, 130.5, 129.6, 128.9, 128.5, 126.9, 126.6, 126.3, 125.4, 122.3, 121.0, 94.2, 87.8, 80.2, 54.5, 52.4, 38.5, 28.4; HRMS (ESI-TOF) *m/z*: $[(MH - CO_2{}^tBu) + H]^+$ Calcd for $C_{22}H_{19}NO_2H$ 330.1489; Found 330.1489.

Methyl (2*S*)-2-(*tert*-butoxycarbonylamino)-3-[4'-(4''-methoxyphenylethynyl)phenyl]propanoate (7d)

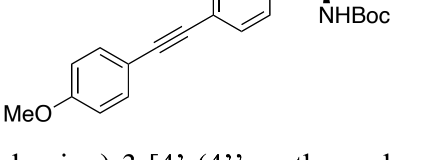

Methyl (2*S*)-2-(*tert*-butoxycarbonylamino)-3-[4'-(4''-methoxyphenylethynyl)phenyl]propanoate (**7d**) was synthesized as described in the general procedure using methyl (2*S*)-2-(*tert*-butoxycarbonylamino)-3-(4-hydroxyphenyl)propanoate (**6**) (0.202 g, 0.684 mmol), caesium carbonate (0.653 g, 2.00 mmol), perfluoro-1-butanesulfonyl fluoride (0.180 mL, 1.02 mmol), 4'-methoxyphenylacetylene (0.260 mL,

2.01 mmol) and XPhos Pd G2 (0.0260 g, 0.0330 mmol) in acetonitrile (3.0 mL). Purification by flash column chromatography, eluting with 20% ethyl acetate in hexane gave methyl (2*S*)-2-(*tert*-butoxycarbonylamino)-3-[4'-(4''-methoxyphenylethynyl)phenyl]propanoate (**7d**) (0.248 g, 89%) as a brown solid. Mp 86–88 °C; IR (neat) 3369, 2954, 1739, 1692, 1518, 1240, 1164, 825 cm^{-1}; $[\alpha]_D^{16}$ +60.6 (*c* 0.1, $CHCl_3$); ^{1}H NMR (400 MHz, $CDCl_3$) δ 7.49–7.40 (m, 4H), 7.10 (d, *J* = 7.8 Hz, 2H), 6.87 (d, *J* = 7.9 Hz, 2H), 4.98 (d, *J* = 8.3 Hz, 1H), 4.64–4.51 (m, 1H), 3.83 (s, 3H), 3.71 (s, 3H), 3.13 (dd, *J* = 14.0, 5.6 Hz, 1H), 3.05 (dd, *J* = 14.0, 6.4 Hz, 1H), 1.42 (s, 9H); $^{13}C\{^1H\}$ NMR (101 MHz, $CDCl_3$) δ 172.3, 159.7, 155.2, 136.1, 133.1, 131.7, 129.4, 122.5, 115.5, 114.1, 89.7, 87.9, 80.1, 55.4, 54.4, 52.4, 38.4, 28.4; HRMS (ESI-TOF) *m/z*: $[M + Na]^+$ Calcd for $C_{24}H_{27}NO_5Na$ 432.1781; Found 432.1791.

Methyl (2*S*)-2-(*tert*-butoxycarbonylamino)-3-[4'-(4''-dimethylaminophenylethynyl)phenyl]propanoate (7e)

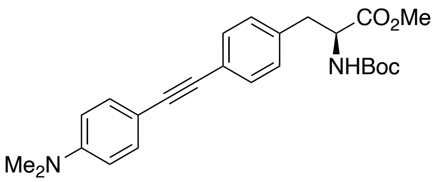

Methyl (2*S*)-2-(*tert*-butoxycarbonylamino)-3-[4'-(4''-dimethylaminophenylethynyl)phenyl]propanoate (**7e**) was synthesized as described in the general procedure using methyl (2*S*)-2-(*tert*-butoxycarbonylamino)-3-(4-hydroxyphenyl)propanoate (**6**) (0.281 g, 0.951 mmol), caesium carbonate (0.917 g, 2.81 mmol), perfluoro-1-butanesulfonyl fluoride (0.240 mL, 1.42 mmol), 4'-dimethylaminophenylacetylene (0.410 g, 2.82 mmol) and XPhos Pd G2 (0.0360 g, 0.0460 mmol) in acetonitrile (4 mL). Purification by flash column chromatography, eluting with 20% ethyl acetate in hexane gave methyl (2*S*)-2-(*tert*-butoxycarbonylamino)-3-[4'-(4''-dimethylaminophenylethynyl)phenyl]propanoate (**7e**) (0.368 g, 91%) as a green solid. Mp 126–128 °C; IR (neat) 3346, 2928, 1731, 1688, 1609, 1522, 1168, 815 cm^{-1}; $[\alpha]_D^{16}$ +72.4 (*c* 0.1, $CHCl_3$); ^{1}H NMR (400 MHz, $CDCl_3$) δ 7.44–7.36 (m, 4H), 7.08 (d, *J* = 8.1 Hz, 2H), 6.66 (d, *J* = 7.6 Hz, 2H), 4.97 (d, *J* = 8.2 Hz, 1H), 4.62–4.53 (m, 1H), 3.71 (s, 3H), 3.15–3.02 (m, 2H), 2.99 (s, 6H), 1.43 (s, 9H); $^{13}C\{^1H\}$ NMR (101 MHz, $CDCl_3$) δ 172.4, 155.2, 150.3, 135.6, 132.8, 131.6, 129.4, 123.0, 112.0, 110.2, 90.9, 87.2, 80.1, 54.5, 52.4, 40.4, 38.4, 28.4; HRMS (APCI-TOF) *m/z*: $[M + H]^+$ Calcd for $C_{25}H_{30}N_2O_4H$ 423.2278; Found 423.2289.

Methyl (2*S*)-2-(*tert*-butoxycarbonylamino)-3-[4'-(4''-cyanophenylethynyl)phenyl]propanoate (7f)

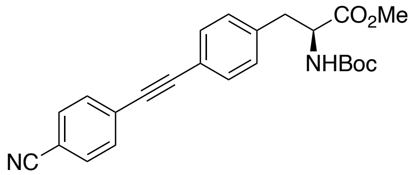

Methyl (2*S*)-2-(*tert*-butoxycarbonylamino)-3-[4'-(4''-cyanophenylethynyl)phenyl]propanoate (**7f**) was synthesized as described in the general procedure using methyl (2*S*)-2-(*tert*-butoxycarbonylamino)-3-(4-hydroxyphenyl)propanoate (**6**) (0.150 g, 0.508 mmol), caesium carbonate (0.495 g, 1.52 mmol), perfluoro-1-butanesulfonyl fluoride (0.140 mL, 0.762 mmol), 4'-cyanophenylacetylene (0.194 g, 1.53 mmol) and XPhos Pd G2 (0.0200 g, 0.0254 mmol) in acetonitrile (2.5 mL). Purification by flash column chromatography, eluting with 20–30% ethyl acetate in hexane gave methyl (2*S*)-2-(*tert*-butoxycarbonylamino)-3-[4'-(4''-cyanophenylethynyl)phenyl]propanoate (**7f**) (0.111 g, 51%) as a yellow solid. Mp 128–130 °C; IR (neat) 3358, 2955, 2205, 1726, 1674, 1293, 1053, 832 cm^{-1}; $[\alpha]_D^{15}$ +27.1 (*c* 0.1, MeOH); ^{1}H NMR (400 MHz, $CDCl_3$) δ 7.63 (d, *J* = 8.4 Hz, 2H), 7.59 (d, *J* = 8.4 Hz, 2H), 7.47 (d, *J* = 8.2 Hz, 2H), 7.15 (d, *J* = 8.2 Hz, 2H), 4.99 (d, *J* = 8.3 Hz, 1H), 4.64–4.55 (m, 1H), 3.72 (s, 3H), 3.16 (dd, *J* = 14.0, 5.8 Hz, 1H), 3.06 (dd, *J* = 14.0, 6.4 Hz, 1H), 1.42 (s, 9H); $^{13}C\{^1H\}$ NMR (101 MHz, $CDCl_3$) δ 172.1, 155.0, 137.5, 132.1, 131.9, 129.5, 128.2, 120.9, 118.5, 111.5, 93.6, 87.9, 80.1, 54.3, 52.3, 38.4, 28.3; HRMS (ESI-TOF) *m/z*: $[(MH - CO_2{}^tBu) + H]^+$ Calcd for $C_{19}H_{16}N_2O_2H$ 305.1285; Found 305.1297.

Methyl (2*S*)-2-(*tert*-butoxycarbonylamino)-3-[4'-(4''-fluorophenylethynyl)phenyl]propanoate (7g)

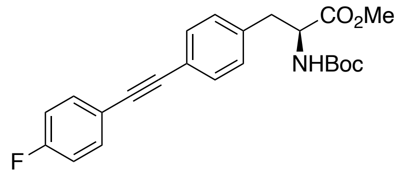

Methyl (2*S*)-2-(*tert*-butoxycarbonylamino)-3-[4'-(4''-fluorophenylethynyl)phenyl]propanoate (**7g**) was synthesized as described in the general procedure using methyl (2*S*)-2-(*tert*-butoxycarbonylamino)-3-(4-hydroxyphenyl)propanoate (**6**) (0.201 g, 0.681 mmol), caesium carbonate (0.653 g, 2.00 mmol), perfluoro-1-butanesulfonyl fluoride (0.180 mL, 1.02 mmol), 4'-fluorophenylacetylene (0.241 g, 2.01 mmol) and XPhos Pd G2 (0.0260 g, 0.0330 mmol) in acetonitrile (2.5 mL). Purification by flash column chromatography, eluting with 20% ethyl acetate in hexane gave methyl (2*S*)-2-(*tert*-butoxycarbonylamino)-3-[4'-(4''-fluorophenylethynyl)phenyl]propanoate (**7g**) (0.256 g, 95%) as a brown solid. Mp 153–156 °C; IR (neat) 3368, 2977, 2359, 1743, 1710, 1515, 1501, 1366, 1219, 1156, 835 cm^{-1}; $[\alpha]_D^{16}$ +75.1 (*c* 0.1, $CHCl_3$); ^{1}H NMR (400 MHz, $CDCl_3$) δ 7.52–7.46 (m, 2H), 7.44 (d, *J* =

8.2 Hz, 2H), 7.12 (d, J = 8.2 Hz, 2H), 7.07–7.00 (m, 2H), 4.99 (d, J = 7.5 Hz, 1H), 4.64–4.55 (m, 1H), 3.71 (s, 3H), 3.14 (dd, J = 14.0, 6.0 Hz, 1H), 3.05 (dd, J = 14.0, 6.0 Hz, 1H), 1.42 (s, 9H); $^{13}C\{^1H\}$ NMR (101 MHz, $CDCl_3$) δ 172.3, 162.6 (d, $^1J_{CF}$ = 249.6 Hz), 155.2, 136.6, 133.6 (d, $^3J_{CF}$ = 8.4 Hz), 131.8, 129.5, 121.9, 119.5 (d, $^4J_{CF}$ = 3.6 Hz), 115.8 (d, $^2J_{CF}$ = 22.0 Hz), 88.9, 88.6, 80.2, 54.4, 52.4, 38.5, 28.4; HRMS (APCI-TOF) *m/z*: [(MH – $CO_2{}^tBu$) + H]$^+$ Calcd for $C_{18}H_{16}FNO_2H$ 298.1238; Found 298.1247.

Methyl (2*S*)-2-(*tert*-butoxycarbonylamino)-3-[4'-(2''-ethynylpyridine)phenyl]propanoate (7h)

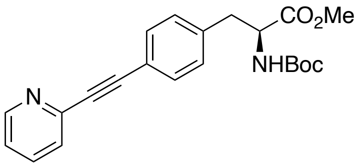

Methyl (2*S*)-2-(*tert*-butoxycarbonylamino)-3-[4'-(2''-ethynylpyridine)phenyl]propanoate (**7h**) was synthesized as described in general procedure **3** using methyl (2*S*)-2-(*tert*-butoxycarbonylamino)-3-(4-hydroxyphenyl)propanoate (**6**) (0.152 g, 0.515 mmol), caesium carbonate (0.496 g, 1.52 mmol), perfluoro-1-butanesulfonyl fluoride (0.140 mL, 0.762 mmol), 2-ethynylpyridine (0.150 g, 1.52 mmol) and XPhos Pd G2 (0.0200 g, 0.0254 mmol) in acetonitrile (2.5 mL) at 85 °C. Purification by flash column chromatography, eluting with 5% diethyl ether in dichloromethane gave methyl (2*S*)-2-(*tert*-butoxycarbonylamino)-3-[4'-(2''-ethynylpyridine)phenyl]propanoate (**7h**) (0.0570 g, 30%) as a green solid. Mp 148–150 °C; IR (neat) 3347, 2977, 2366, 2223, 1745, 1702, 1583, 1508, 1466, 1362, 1160, 783 cm^{-1}; $[\alpha]_D^{20}$ +19.2 (*c* 0.1, $CHCl_3$); ^1H NMR (400 MHz, $CDCl_3$) δ 8.61 (br d, J = 4.0 Hz, 1H), 7.67 (td, J = 7.6, 2.0 Hz, 1H), 7.54–7.48 (m, 3H), 7.23 (ddd, J = 7.6, 4.8, 1.2 Hz, 1H), 7.13 (d, J = 8.0 Hz, 2H), 5.00 (d, J = 8.0 Hz, 1H), 4.64–4.57 (m, 1H), 3.71 (s, 3H), 3.14 (dd, J = 14.0, 5.6 Hz, 1H), 3.05 (dd, J = 14.0, 6.0 Hz, 1H), 1.41 (s, 9H); $^{13}C\{^1H\}$ NMR (101 MHz, $CDCl_3$) δ 172.2, 155.1, 150.2, 143.6, 137.4, 136.3, 132.3, 129.5, 127.3, 122.9, 121.1, 89.1, 88.9, 80.2, 54.4, 52.4, 38.5, 28.4; HRMS (APCI-TOF) *m/z*: [M + H]$^+$ Calcd for $C_{22}H_{24}N_2O_4H$ 381.1809; Found 381.1810.

Methyl (2*S*)-2-(*tert*-butoxycarbonylamino)-3-[4'-(trimethylsilylethynyl)phenyl]propanoate (7i)[3]

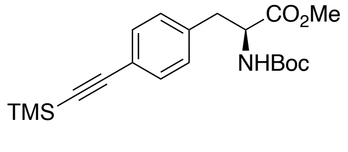

Methyl (2*S*)-2-(*tert*-butoxycarbonylamino)-3-(4-hydroxyphenyl)propanoate (**6**) (0.150 g, 0.508 mmol) was dissolved in dry acetonitrile (2.5 mL) and degassed for 0.2 h. Triethylamine (0.210 mL, 1.52 mmol) and perfluoro-1-butanesulfonyl fluoride (0.140 mL, 0.762 mmol) were added. The reaction mixture was

heated to 60 °C and stirred for 2 h. Trimethylsilyl acetylene (0.220 mL, 1.52 mmol) and XPhos Pd G2 (0.0120 g, 0.0150 mmol) were added. The reaction mixture was degassed for 0.2 h, heated to 70 °C and stirred for 5 h. A further portion of trimethylsilyl acetylene (0.0400 mL, 0.276 mmol) and XPhos Pd G2 (0.0120 g, 0.0150 mmol) were added. The reaction mixture was degassed for 0.2 h, heated to 70 °C and stirred for 18 h. The reaction mixture was cooled to room temperature, diluted in ethyl acetate (30 mL) and washed with water (3 × 30 mL). The organic layer was dried ($MgSO_4$), filtered and concentrated *in vacuo*. Purification by flash column chromatography, eluting with 20% diethyl ether in hexane gave methyl (2*S*)-2-(*tert*-butoxycarbonylamino)-3-[4'-(trimethylsilylethynyl)phenyl]propanoate (**7i**) (0.178 g, 95%) as an orange oil. $[\alpha]_D^{22}$ +72.1 (*c* 0.1, $CHCl_3$); Spectroscopic data was consistent with the literature.[3] ^{1}H NMR (400 MHz, $CDCl_3$) δ 7.39 (d, J = 8.2 Hz, 2H), 7.06 (d, J = 8.2 Hz, 2H), 4.94 (d, J = 8.4 Hz, 1H), 4.60–4.52 (m, 1H), 3.69 (s, 3H), 3.10 (dd, J = 14.0, 5.6 Hz, 1H), 3.03 (dd, J = 14.0, 6.0 Hz, 1H), 1.42 (s, 9H), 0.24 (s, 9H); ^{13}C{^{1}H} NMR (101 MHz, $CDCl_3$) δ 172.2, 155.1, 136.8, 132.3, 129.3, 122.0, 104.9, 94.5, 80.2, 54.4, 52.4, 38.4, 28.4, 0.1; MS (ESI) *m/z* 276 [(M – $CO_2{}^t$Bu) + H^+, 100].

(2*S*)-2-(*tert*-Butoxycarbonylamino)-3-[4'-(1''-ethynylnaphthalene)phenyl]propanoic acid (9)

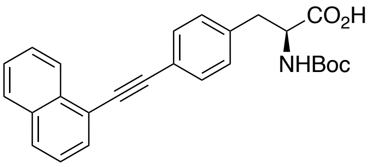

To a stirred solution of methyl (2*S*)-2-(*tert*-butoxycarbonylamino)-3-[4'-(1''-ethynylnaphthalene)phenyl]propanoate (**7c**) (0.172 g, 0.401 mmol) in methanol (6 mL) and 1,4-dioxane (3 mL) at 60 °C was added caesium carbonate (0.170 g, 0.522 mmol) followed by water (3 mL). The reaction mixture was stirred at 60 °C overnight then cooled to room temperature and concentrated *in vacuo*. The reaction mixture diluted in water (20 mL), acidified to pH 1 using 1 M aqueous hydrochloric acid and extracted with ethyl acetate (3 × 20 mL). The organic layers were combined, dried ($MgSO_4$), filtered and concentrated *in vacuo* to give (2*S*)-2-(*tert*-butoxycarbonylamino)-3-[4'-(1''-ethynylnaphthalene)phenyl]propanoic acid (**9**) in a quantitative yield as a pale yellow solid (0.166 g). Mp 71–74 °C; IR (neat) 3320, 2977, 2928, 2362, 1715, 1505, 1394, 1162, 767 cm^{-1}; $[\alpha]_D^{17}$ +31.1 (*c* 0.1, $CHCl_3$); NMR spectra showed a 2:1 mixture of rotamers. Only signals for the major rotamer are recorded. ^{1}H NMR (400 MHz, $CDCl_3$) δ 10.73 (br s, 1H), 8.43 (d, J = 8.4 Hz, 1H), 7.84 (m, 2H), 7.74 (d, J = 7.2 Hz, 1H), 7.64–7.48 (m, 4H), 7.43 (dd, J = 8.4, 7.2 Hz, 1H), 7.25–7.19 (m, 2H), 5.02 (d, J = 8.4 Hz, 1H), 4.69–4.59 (m, 1H), 3.27 (dd, J = 14.0, 6.4 Hz, 1H), 3.27 (dd, J = 14.0, 6.4 Hz, 1H), 1.45 (s, 9H); ^{13}C{^{1}H} NMR (101 MHz, $CDCl_3$) δ 176.5, 155.5, 136.5, 133.35, 133.30, 131.9, 130.5, 129.7, 128.8, 128.8, 126.9, 126.6, 126.3, 125.4, 122.3, 121.0, 94.2, 87.9, 80.6, 54.3, 37.9 28.4; HRMS (ESI-TOF) *m/z*: [M − H]$^-$ Calcd for $C_{26}H_{24}NO_4$ 414.1711; Found 414.1704.

(2*S*)-2-Amino-3-[4'-(1''-ethynylnaphthalene)phenyl]propanoic acid hydrochloride (8)[4]

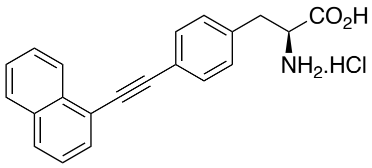

To a stirred solution of (2*S*)-2-(*tert*-butoxycarbonylamino)-3-[4'-(1''-ethynylnaphthalene)phenyl]propanoic acid (**9**) (0.101 g, 0.243 mmol) in 1,4-dioxane (1 mL) was added 2 M hydrochloric acid in 1,4-dioxane (1 mL) dropwise. The reaction mixture was stirred at room temperature for 23 h. The reaction mixture was cooled to 0 °C for 0.5 h and filtered. The precipitate was washed with diethyl ether and dried to give (2*S*)-2-amino-3-[4'-(1''-ethynylnaphthalene)phenyl]propanoic acid hydrochloride (**8**) as an off-white solid (0.0489 g, 64%). Mp 265–267 °C; $[\alpha]_D^{15}$ −9.4 (*c* 0.1, MeOH); Spectroscopic data was consistent with the literature.[4] ^{1}H NMR (400 MHz, CD_3OD) δ 8.38 (d, *J* = 8.0 Hz, 1H), 7.95–7.88 (m, 2H), 7.75 (dd, *J* = 7.2, 1.2 Hz, 1H), 7.67 (d, *J* = 8.2 Hz, 2H), 7.64–7.46 (m, 3H), 7.39 (d, *J* = 8.2 Hz, 2H), 4.15 (dd, *J* = 8.0, 5.2 Hz, 1H), 3.38 (dd, *J* = 14.4, 5.2 Hz, 1H), 3.17 (dd, *J* = 14.4, 8.0 Hz, 1H); $^{13}C\{^1H\}$ NMR (101 MHz, CD_3OD) δ 171.0, 136.2, 134.7, 134.4, 133.1, 131.4, 130.9, 130.0, 129.5, 128.0, 127.6, 126.8, 126.4, 124.1, 121.7, 94.8, 88.7, 54.9, 37.1; HRMS (ESI-TOF) *m/z*: $[M]^+$ Calcd for $C_{21}H_{18}NO_2$ 316.1332; Found 316.1334.

(2*S*)-2-(*tert*-Butoxycarbonylamino)-3-[4'-(1''-ethynylnaphthalene)phenyl]-1-propanamide-L-phenylalanine methyl ester (10a)

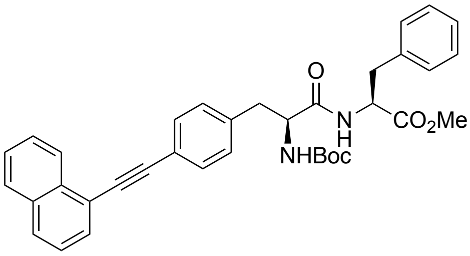

To a stirred solution of (2*S*)-2-(*tert*-butoxycarbonylamino)-3-[4'-(1''-ethynylnaphthalene)phenyl]propanoic acid (**9**) (39.0 mg, 0.0940 mmol) in acetonitrile (3 mL) at 0 °C was added L-phenylalanine methyl ester hydrochloride (22.0 mg, 0.102 mmol) and 1-hydroxybenzotriazole hydrate (6.00 mg, 0.0470 mmol). The reaction mixture was stirred at 0 °C for 5 minutes prior to the addition of *N,N*-diisopropylethylamine (0.0490 mL, 0.282 mmol) and PyBOP® (75.0 mg, 0.141 mmol). After 0.5 h, the reaction mixture was warmed to room temperature overnight and then concentrated *in vacuo*. The reaction mixture was diluted in ethyl acetate (30 mL), washed with 1 M hydrochloric acid (30 mL) and then brine (30 mL). The organic layer was dried ($MgSO_4$), filtered and concentrated *in vacuo*. Purification by flash column chromatography eluting in 30% ethyl acetate in hexane gave (2*S*)-2-(*tert*-butoxycarbonylamino)-3-[4'-(1''-ethynylnaphthalene)phenyl]-1-propanamide-L-phenylalanine methyl ester (**10a**) as a white solid (42.7 mg, 79%). Mp 122–150 °C; IR (neat) 3309, 3290, 2965, 1741, 1654, 1511, 1366, 1216, 755 cm^{-1}; $[\alpha]_D^{17}$ +60.0 (*c* 0.1, $CHCl_3$); ^1H NMR (400 MHz, $CDCl_3$) δ 8.42 (dd, *J* = 8.0, 0.8 Hz, 1H), 7.89–7.82 (m, 2H), 7.75 (dd, *J* = 7.2, 1.4 Hz, 1H), 7.62–7.51 (m, 4H), 7.46 (dd, *J* = 8.0, 7.2 Hz, 1H), 7.30–7.19 (m, 5H), 7.06–7.00 (m, 2H), 6.29 (d, *J* = 7.2 Hz, 1H), 5.00 (br s, 1H), 4.86–4.74 (m, 1H), 4.37 (d, *J* = 6.0 Hz, 1H), 3.70 (s, 3H), 3.14–3.00 (m, 4H), 1.43 (s, 9H); $^{13}C\{^1H\}$ NMR (101 MHz, $CDCl_3$) δ 171.5, 170.7, 155.4, 137.1, 135.7, 133.4, 133.3, 132.0, 130.5, 129.6, 129.3, 128.9, 128.7, 128.5, 127.3, 126.9, 126.6, 126.3, 125.4, 122.2, 121.0, 94.2, 87.8, 80.5, 55.7, 53.4, 52.5, 38.4, 38.1, 28.4; HRMS (ESI-TOF) *m/z*: $[M + Na]^+$ Calcd for $C_{36}H_{36}N_2O_5Na$ 599.2516; Found 599.2513.

(2*S*)-2-(*tert*-Butoxycarbonylamino)-3-[4'-(1''-ethynylnaphthalene)phenyl]-1-propanamide-L-tryptophan methyl ester (10b)

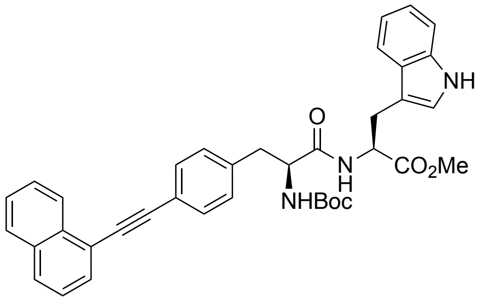

To a stirred solution of (2*S*)-2-(*tert*-butoxycarbonylamino)-3-[4'-(1''-ethynylnaphthalene)phenyl]propanoic acid (**9**) (56.0 mg, 0.135 mmol) in acetonitrile (2.5 mL) at 0 °C was added L-tryptophan methyl ester hydrochloride (38.0 mg, 0.148 mmol) and 1-hydroxybenzotriazole hydrate (9.00 mg, 0.0675 mmol). The reaction mixture was stirred at 0 °C for 5 minutes prior to the addition of *N,N*-diisopropylethylamine (0.0700 mL, 0.405 mmol) and PyBOP® (105 mg, 0.203 mmol). After a further 0.5 h, the reaction mixture was warmed to room temperature overnight then concentrated *in vacuo*. The reaction mixture was diluted in ethyl acetate (30 mL), washed in 1 M hydrochloric acid (30 mL) and then brine (30 mL). The organic layer was dried ($MgSO_4$), filtered and concentrated *in vacuo*. Purification by flash column chromatography eluting in 40% ethyl acetate in hexane gave (2*S*)-2-(*tert*-butoxycarbonylamino)-3-[4'-(1''-ethynylnaphthalene)phenyl]-1-propanamide-L-tryptophan methyl ester (**10b**) as a white solid (63.0 mg, 76%). Mp 88–91 °C; IR (neat) 3314, 2979, 2921, 1656, 1506, 1438, 1336, 1250, 1213, 1161, 742 cm^{-1}; $[\alpha]_D^{17}$ +55.6 (*c* 0.1, $CHCl_3$); ^1H NMR (400 MHz, $CDCl_3$) δ 8.43 (dd, J = 8.0, 0.8 Hz, 1H), 8.25 (br s, 1H), 7.90–7.82 (m, 2H), 7.76 (dd, J = 7.2, 1.2 Hz, 1H), 7.62–7.41 (m, 6H), 7.33 (d, J = 8.0 Hz, 1H), 7.22–7.15 (m, 3H), 7.11 (t, J = 7.4 Hz, 1H), 6.87 (d, J = 2.8 Hz, 1H), 6.38 (d, J = 7.2 Hz, 1H), 5.07 (d, J = 5.6 Hz, 1H), 4.93–4.82 (m, 1H), 4.45–4.17 (m, 1H), 3.66 (s, 3H), 3.30 (dd, J = 14.6, 5.6 Hz, 1H), 3.25 (dd, J = 14.6, 5.6 Hz, 1H), 3.04 (d, J = 6.8 Hz, 2H), 1.41 (s, 9H); $^{13}C\{^1H\}$ NMR (101 MHz, $CDCl_3$) δ 171.9, 170.8, 155.4, 137.2, 136.2, 133.3, 131.9, 130.5, 129.6, 128.9, 128.5, 127.6, 126.9, 126.6, 126.3, 125.4, 123.0, 122.4, 122.0, 120.9, 119.8, 118.5, 111.5, 109.7, 94.3, 87.8, 80.3, 55.7, 53.1, 52.5, 38.6, 28.4, 27.7; HRMS (ESI-TOF) *m/z*: $[M + Na]^+$ Calcd for $C_{38}H_{37}N_3O_5Na$ 638.2625; Found 638.2622.

3. Photophysical Data for all Compounds.

Table S1. Photophysical Data of α-Amino Acids 7a–h and 8.

amino acid	λ_{Abs} (nm)[a]	ε (cm^{-1} M^{-1})	λ_{Em} (nm)[a]	Φ_F[b]	brightness (cm^{-1} M^{-1})
**7a**	284	24900	314	---	---
**7b**	274	39200	341	0.23	8950
**7c**	316, 336	24100	343, 359	0.65	15550
**7d**	292	30200	326	---	---
**7e**	326	35400	423	0.03	1050
**7f**	302	34400	356	0.21	7340
**7g**	282	29400	324	---	---
**7h**	296	20400	334	0.06	1270
**8**	316, 346	25300	342, 362	0.57	14480

[a]Spectra were recorded at concentrations of 5 μM in methanol. [b]Quantum yields (Φ_F) were determined in methanol using anthracene and L-tryptophan as standards. Due to weak emission spectra, the quantum yields for amino acids **7a**, **7d** and **7g** were not measured.

Absorption and Emission Spectra for 7a (5 μM). Excitation at 283 nm.

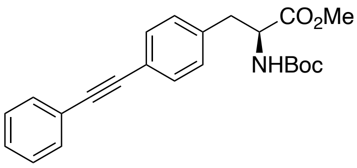

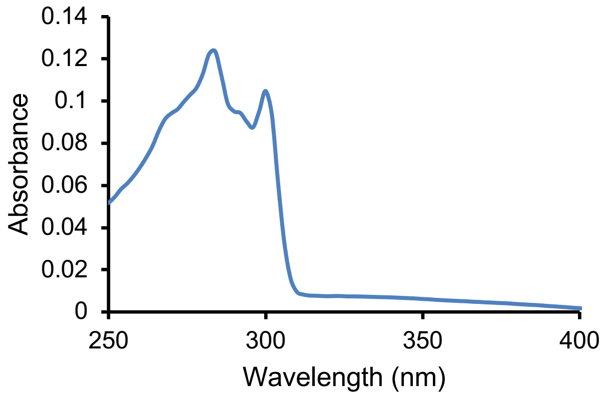

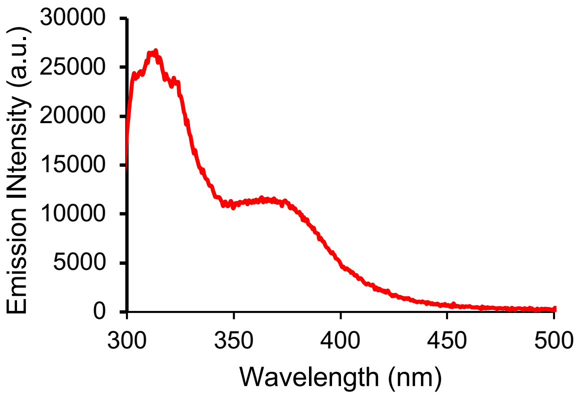

Absorption and Emission Spectra for 7b (5 μM). Excitation at 304 nm.

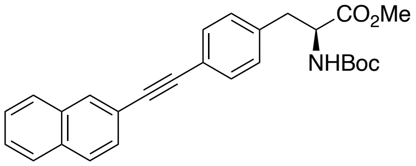

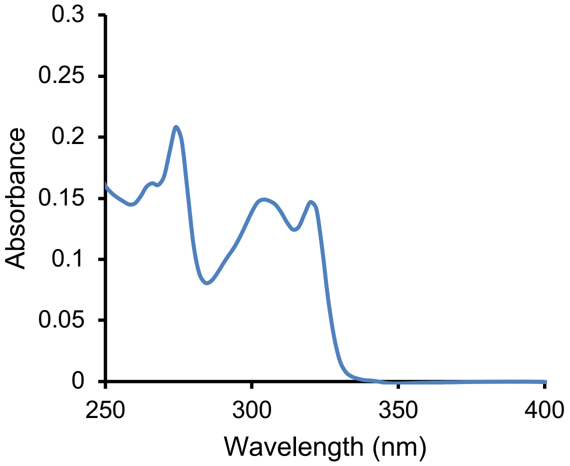

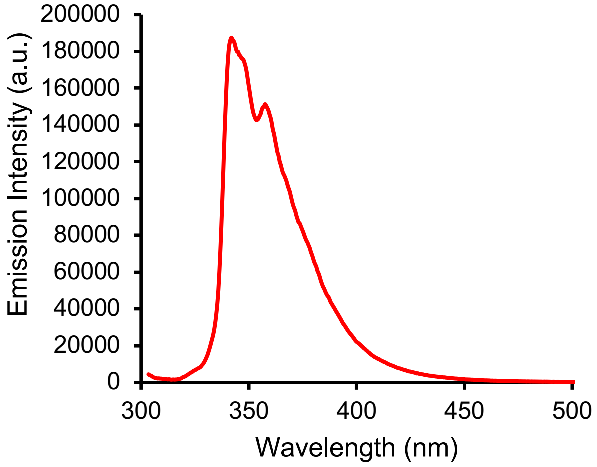

Absorption and Emission Spectra for 7c (5 μM). Excitation at 316 nm.

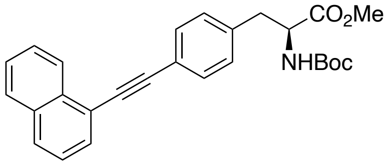

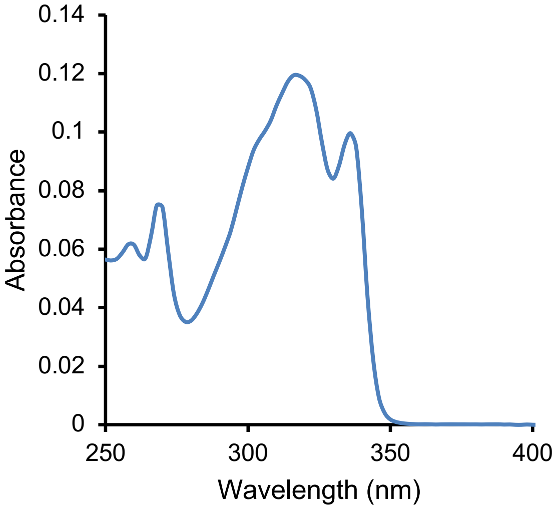

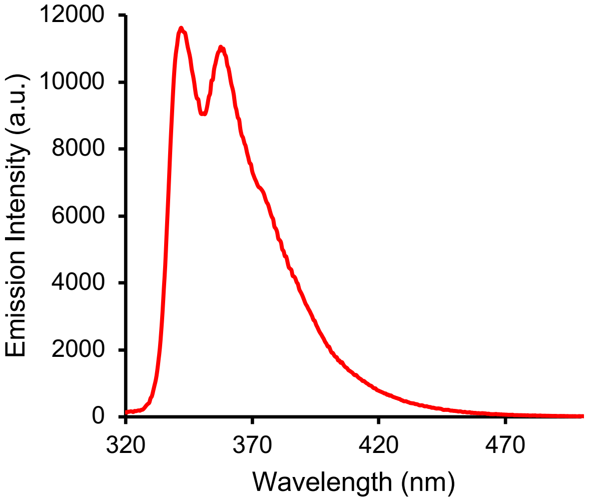

Absorption and Emission Spectra for 7d (5 μM). Excitation at 290 nm.

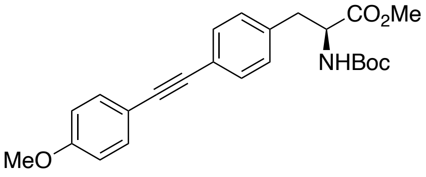

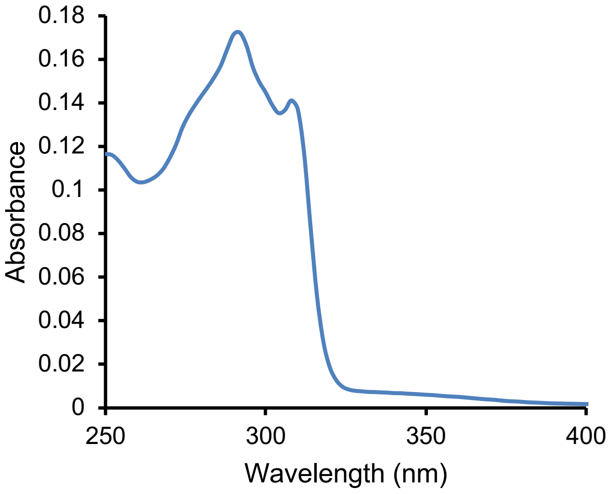

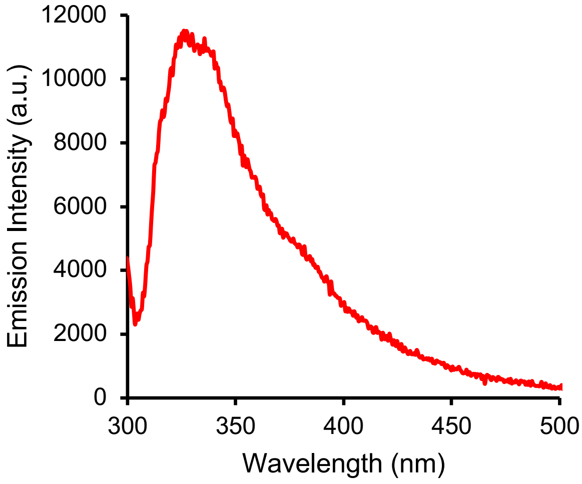

Absorption and Emission Spectra for 7e (5 μM). Excitation at 320 nm.

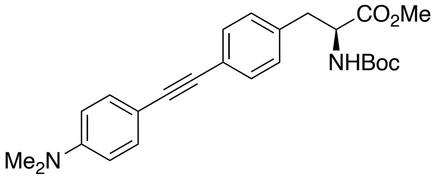

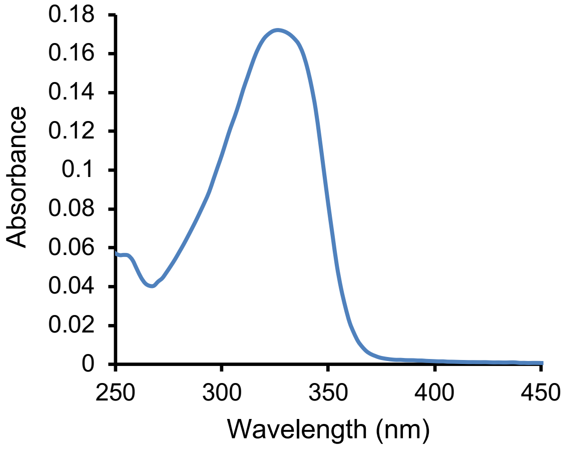

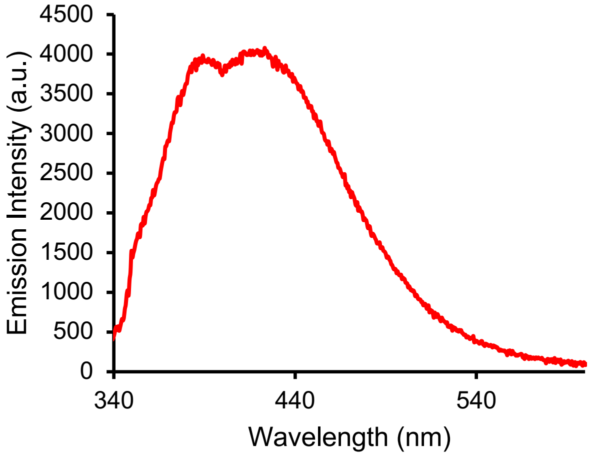

Absorption and Emission Spectra for 7f (5 µM). Excitation at 300 nm.

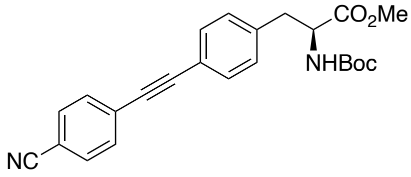

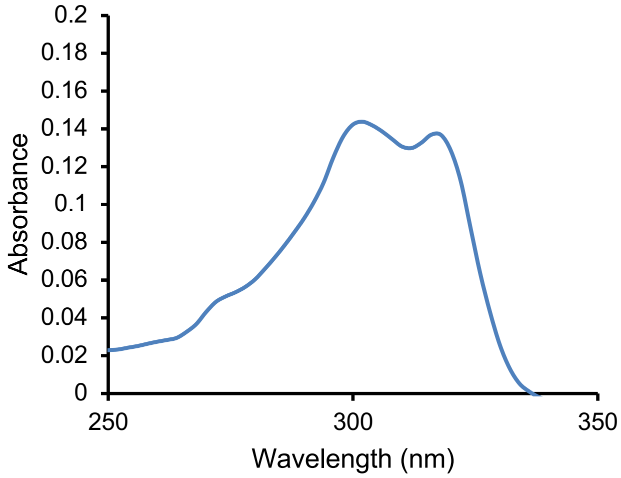

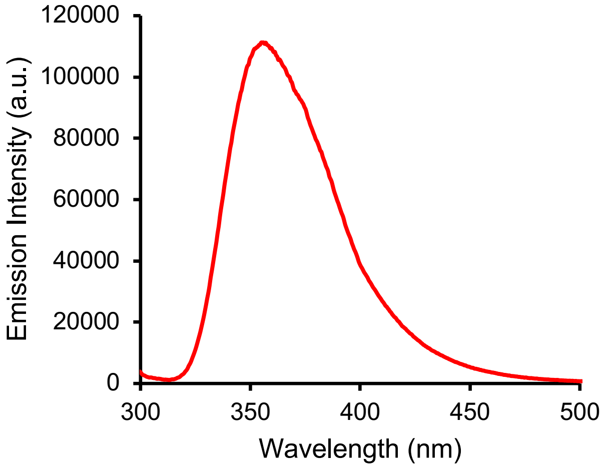

Absorption and Emission Spectra for 7g (5 µM). Excitation at 300 nm.

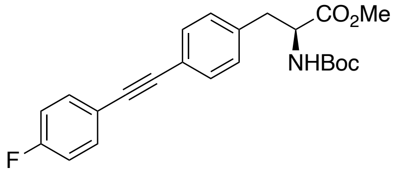

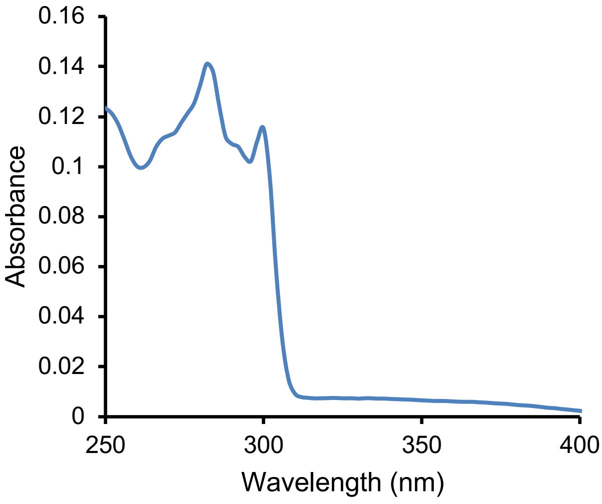

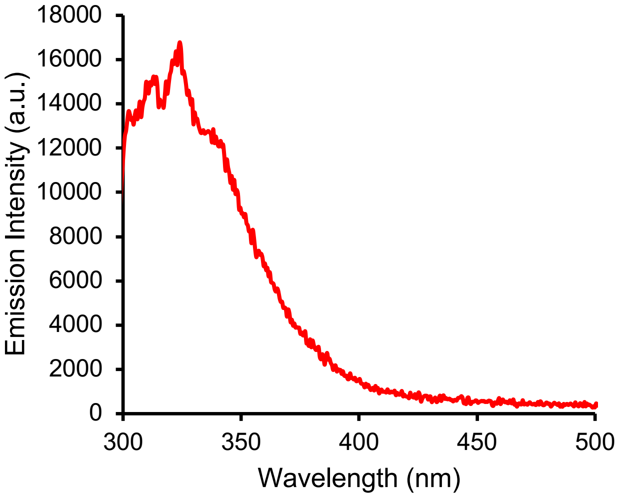

Absorption and Emission Spectra for 7h (5 μM). Excitation at 300 nm.

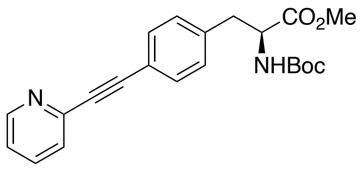

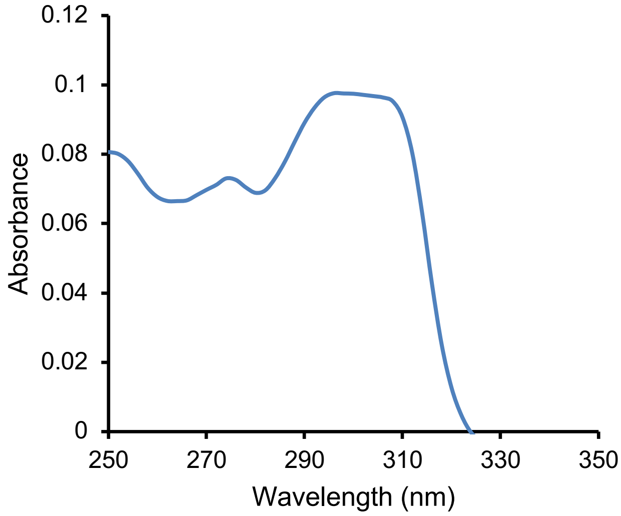

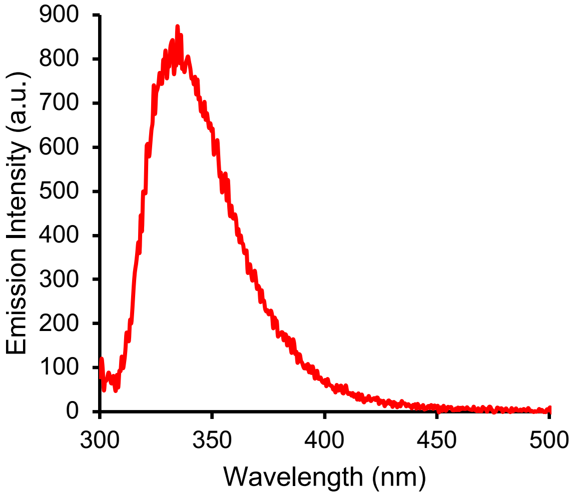

Solvatochromic Study with 7c (5 μM in Various Solvents).

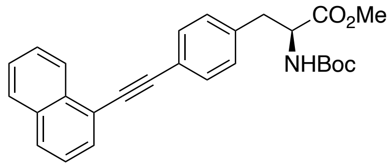

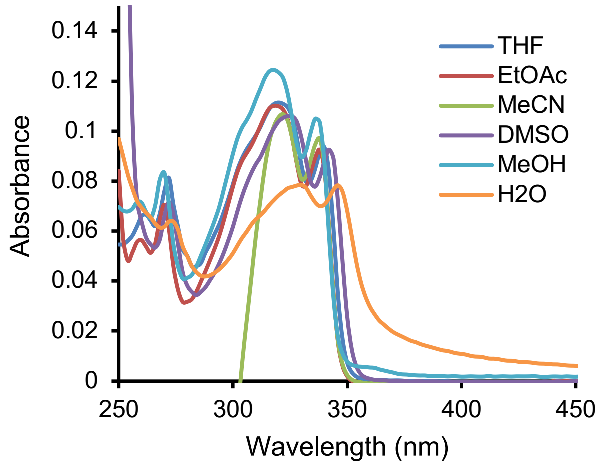

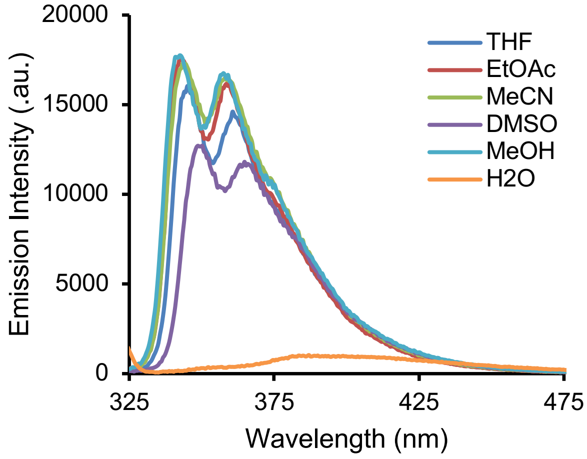

Viscosity Study with 7c (5 μM in Various Portions of Ethylene Glycol in Methanol).

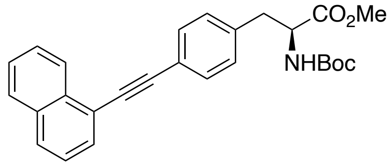

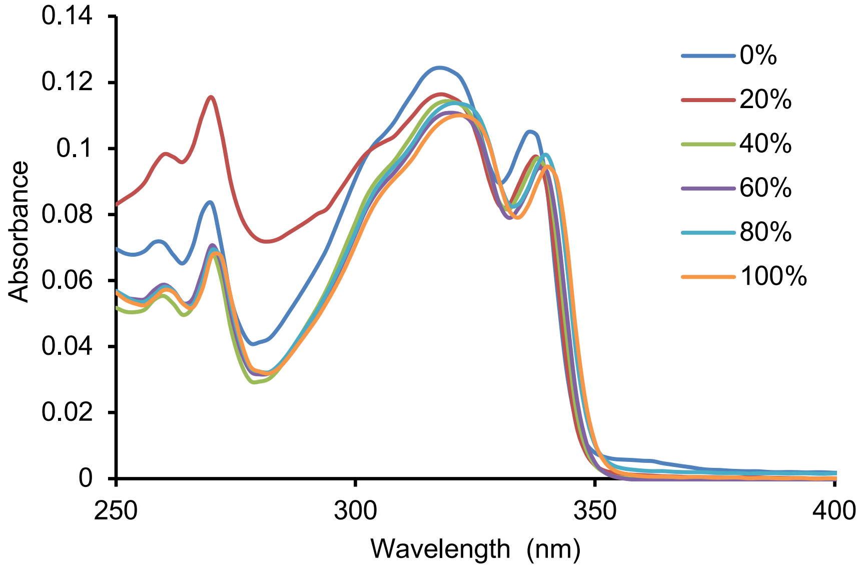

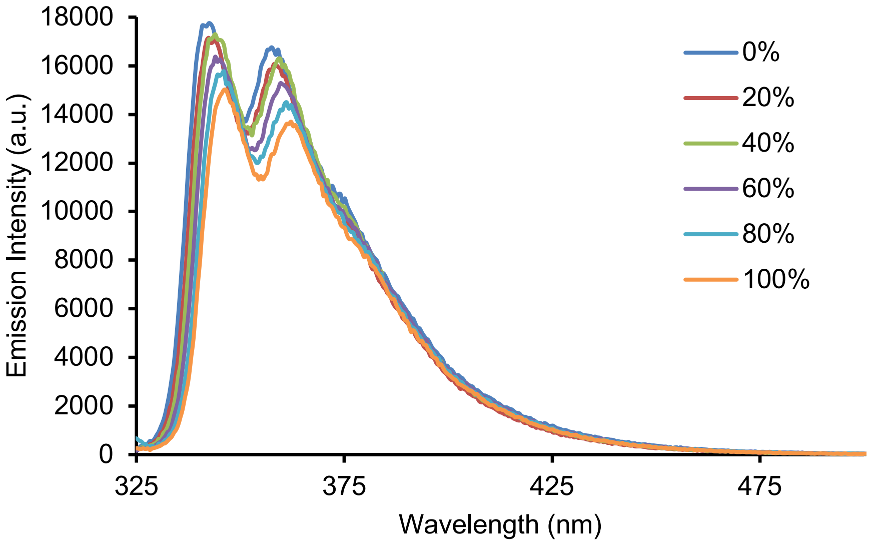

Aggregation Study with 7c (Various Concentrations in Methanol).

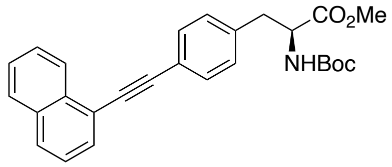

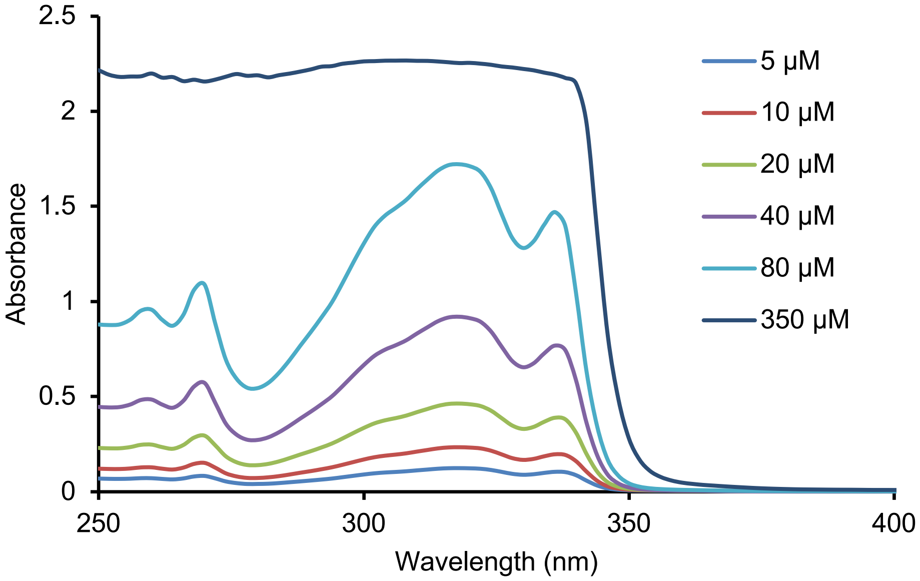

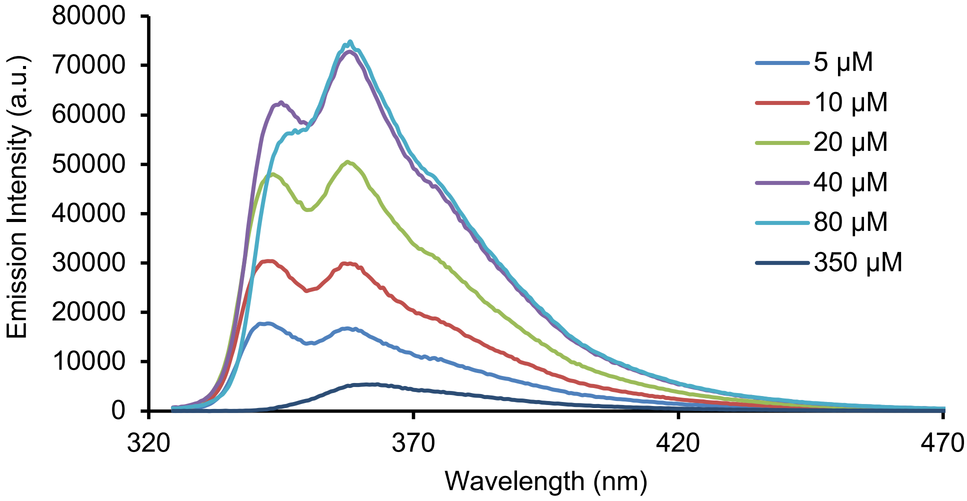

Absorption and Emission Spectra of 7c in Liposomes versus Phosphate-Buffered Saline (PBS)

Liposome preparation: Liposomes were prepared as described in literature.[5] L-α-Phosphatidylcholine (egg yolk) (75 mg) and cholesterol (11 mg) (7:1 PC:cholesterol) were dissolved in chloroform (15 mL). The solution was concentrated *in vacuo* to give a thin film on the flask wall. Sodium phosphate buffer (10 mM, PBS, pH 7.4) (10 mL) was added. The flask was swirled in a water bath at 37 °C for 10 minutes to form a suspension. The suspension was kept at 25 °C for 2 h, sonicated for 3 minutes, then kept at 25 °C for a further 2 h. The suspension was centrifuged at 4000 rcf for 0.5 h. The pellet was resuspended in 10 mM PBS (10 mL) to give a 7.5 mg/mL PC liposome concentration. Liposomes were stored in the fridge and allowed to come to room temperature prior to use. Stock solutions were prepared in methanol, using 3 mg of the compound to make up a 10 mL solution using a volumetric flask. Next, 5 μM sample solutions (total volume 1 mL) were prepared from the stock solution with either PBS or liposome solution. PBS and liposome solutions were incubated at 25 °C for 0.5 h prior to use.

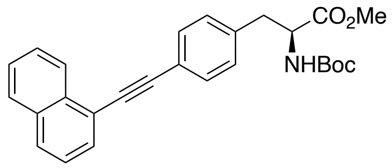

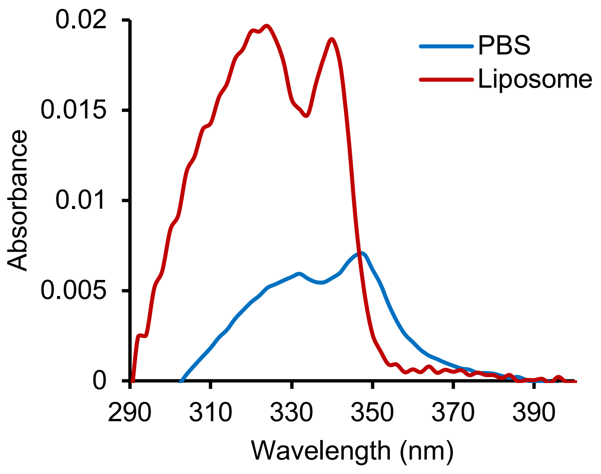

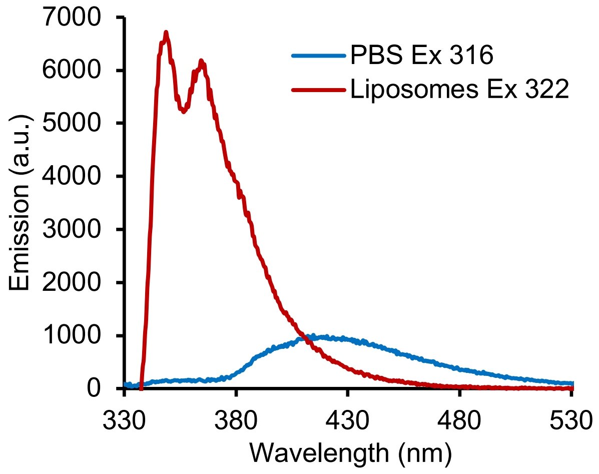

Absorption and Emission Spectra of Amino Acid 8 (5 μM in MeOH)

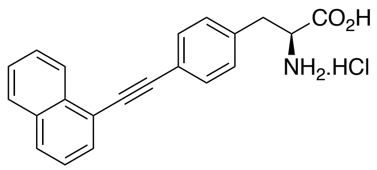

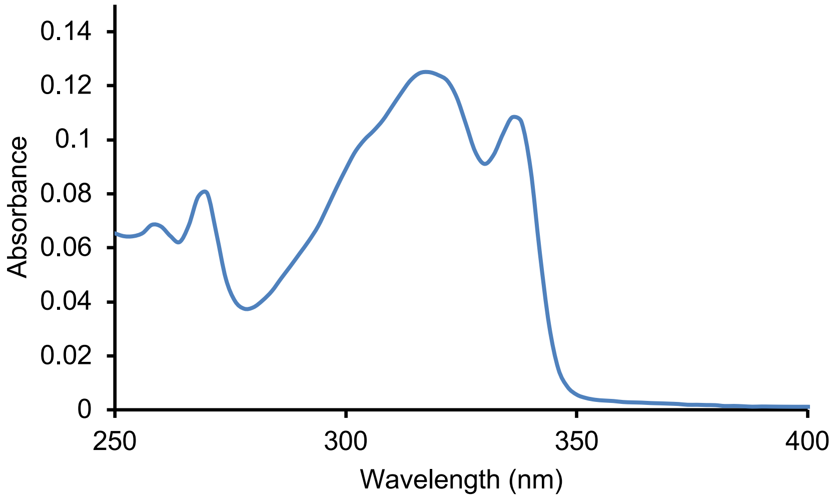

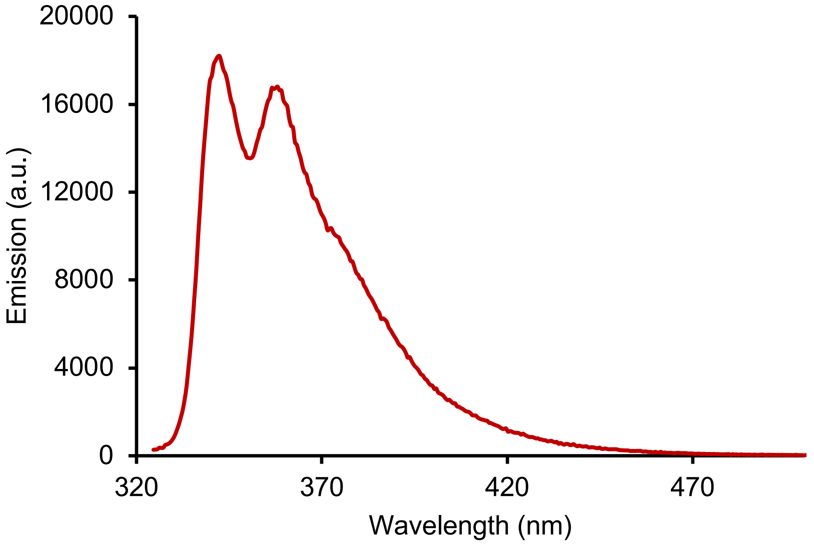

Absorption and Emission Spectra of Dipeptide 10a (5 μM in MeOH)

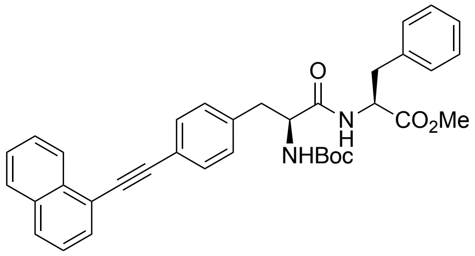

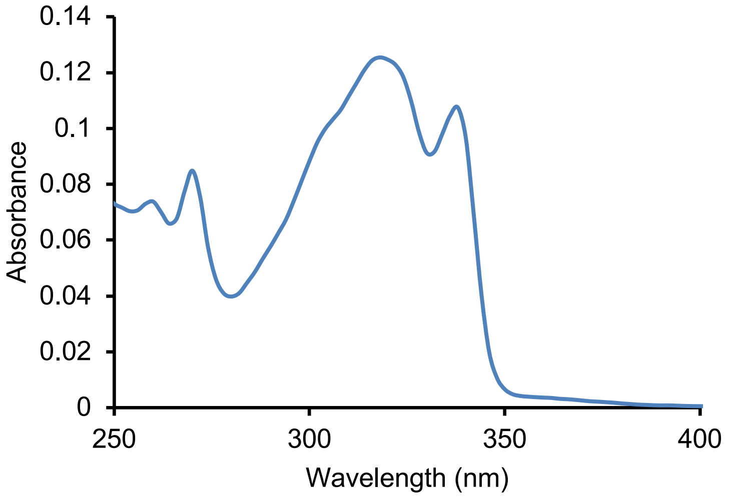

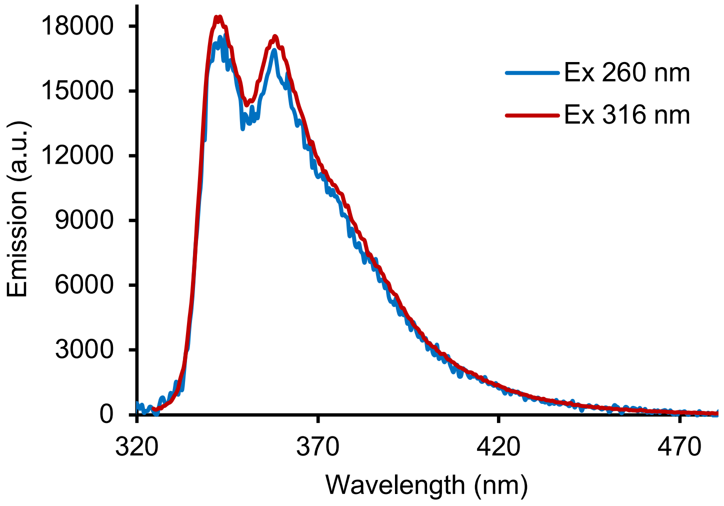

Absorption and Emission Spectra of Dipeptide 10b (5 μM in MeOH)

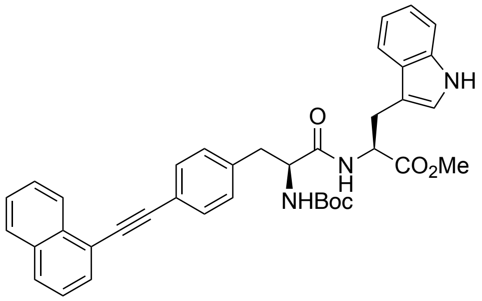

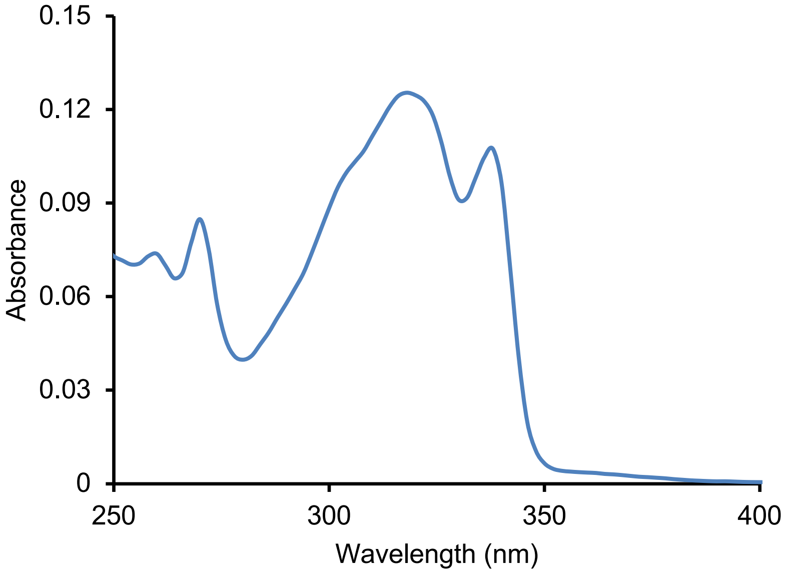

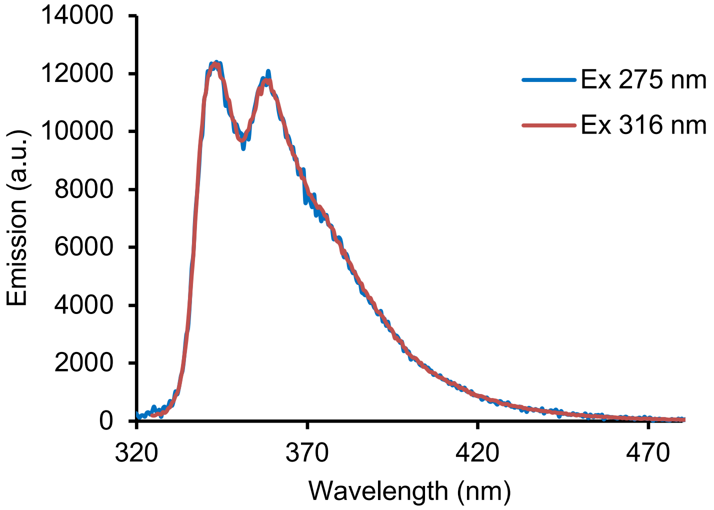

4. References

1. Lakowicz, J. R. Principles of Fluorescence Spectroscopy, Springer, 3rd Edition, New York, **2006**.

2. Williams, A. T. R.; Winfield, S. A.; Miller, J. N. Relative Fluorescence Quantum Yields Using a Computer-controlled Luminescence Spectrometer. *Analyst* **1983**, *108*, 1067–1071.

3. Verlinden, S.; Ballet, S.; Verniest, G. Synthesis of Heterocycle-Bridged Peptidic Macrocycles through 1,3-Diyne Transformations. *Eur. J. Org. Chem*. **2016**, 5807–5812.

4. Saghyan, A. S.; Mkrtchyan, A. F.; Mardiyan, Z. Z.; Hayriyan, L. A.; Karapetyan, A. J.; Belokon, Y. N.; Ehlers, P.; Langer, P. Synthesis of Enantiomerically Enriched Alkynylaryl-Substituted α-Amino Acids through Sonogashira Reactions. *ChemistrySelect* **2019**, *4*, 13806–13809.

5. van Rooijen, N.; van Nieuwmegen, R. Elimination of Phagocytic Cells in the Spleen after Intravenous Injection of Liposome-Encaspulated Dichloromethylene Diphosphonate. *Cell Tissue Res*. **1984**, *238*, 355–358.

5. ^{1}H and ^{13}C NMR Spectra for all Compounds

^{1}H NMR (400 MHz, $CDCl_3$)

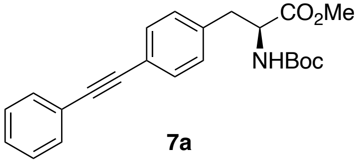

7a

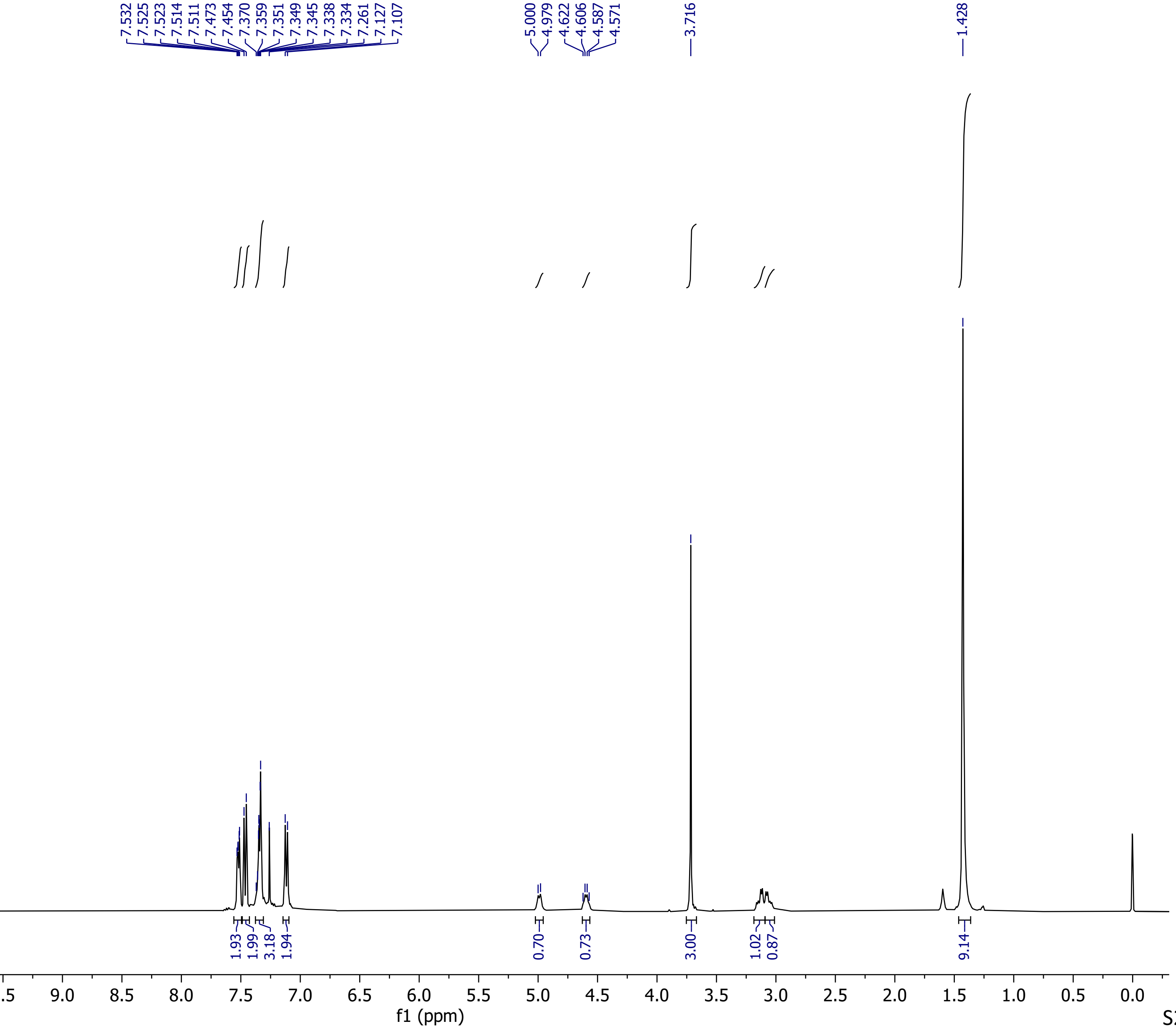

$^{13}C\{^1H\}$ NMR (101 MHz, $CDCl_3$)

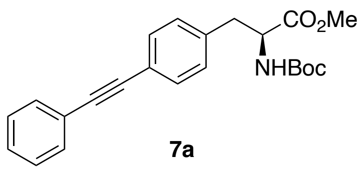

7a

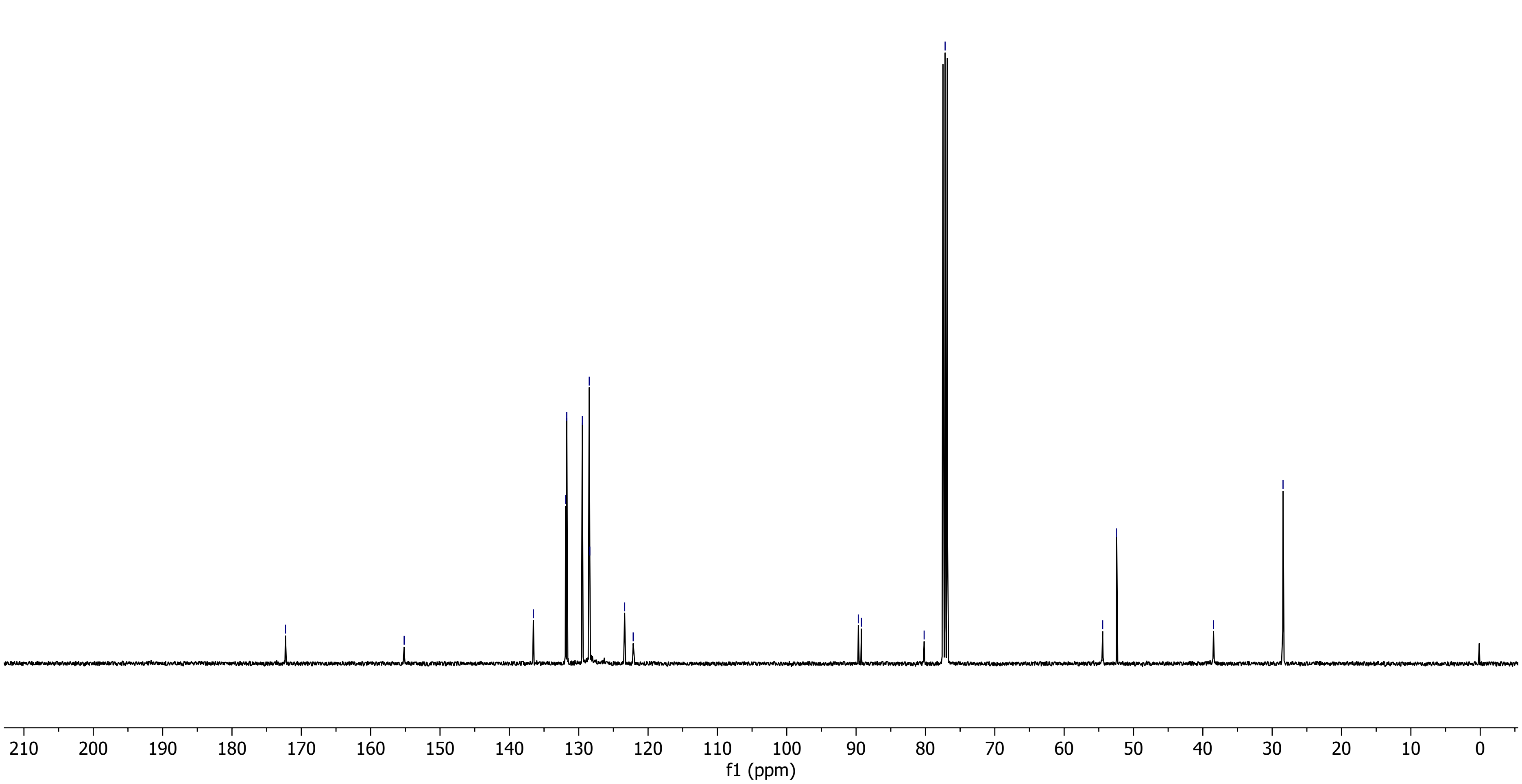

^{1}H NMR (400 MHz, $CDCl_3$)

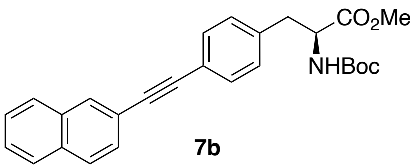

7b

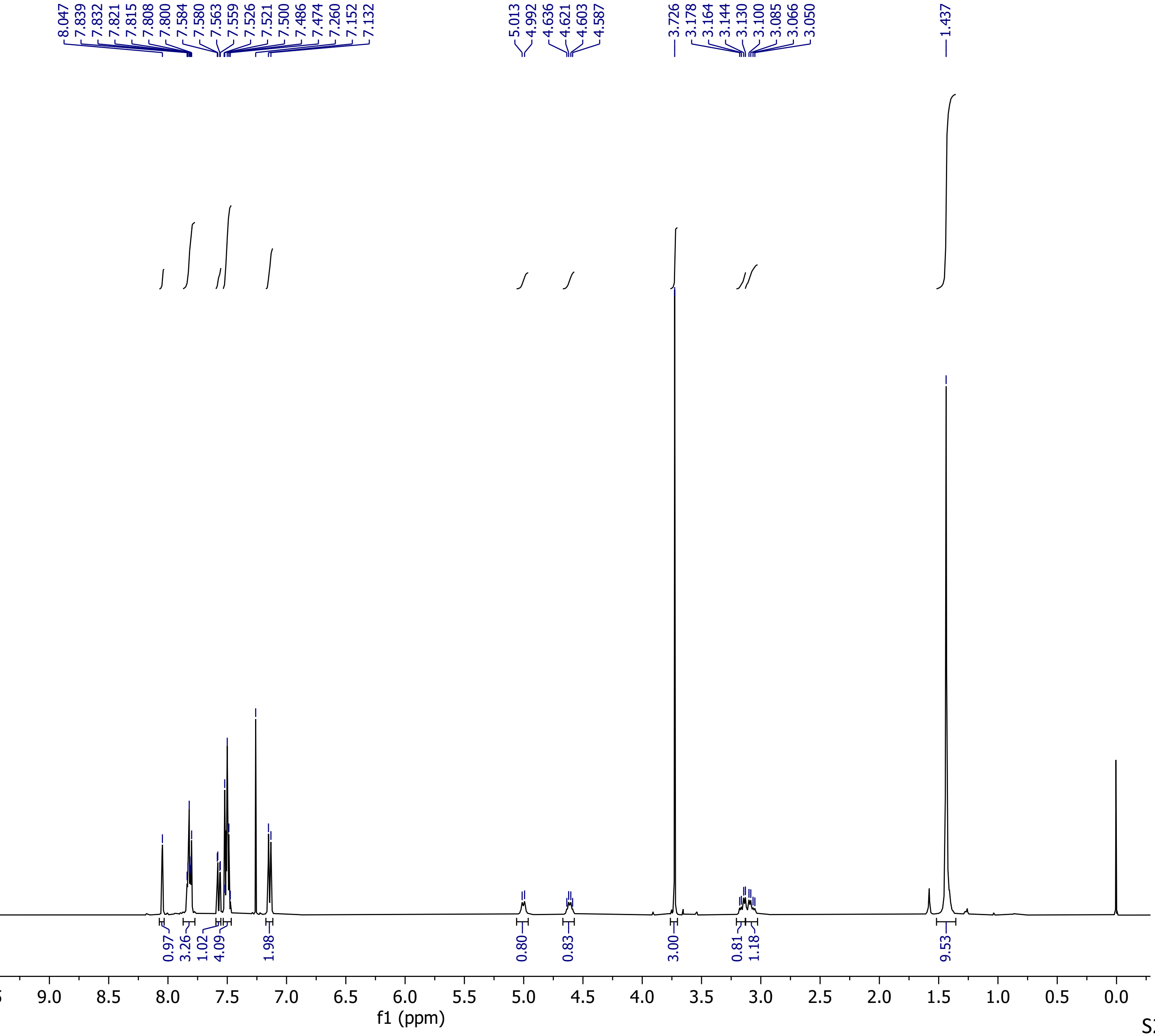

$^{13}C\{^{1}H\}$ NMR (101 MHz, $CDCl_3$)

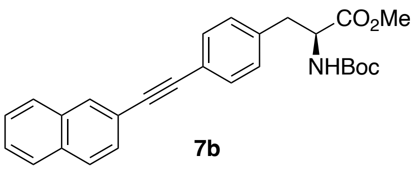

7b

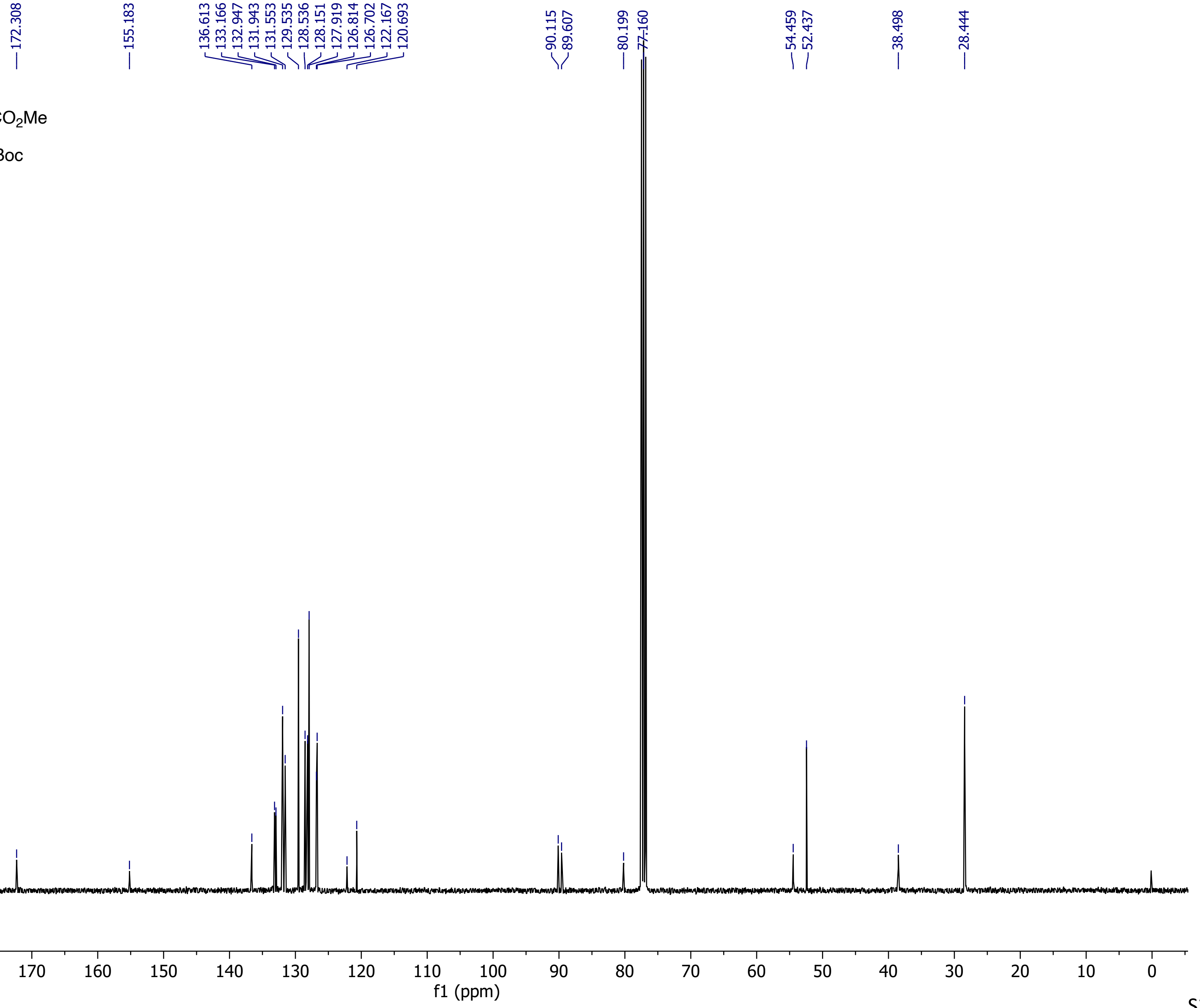

^{1}H NMR (400 MHz, $CDCl_3$)

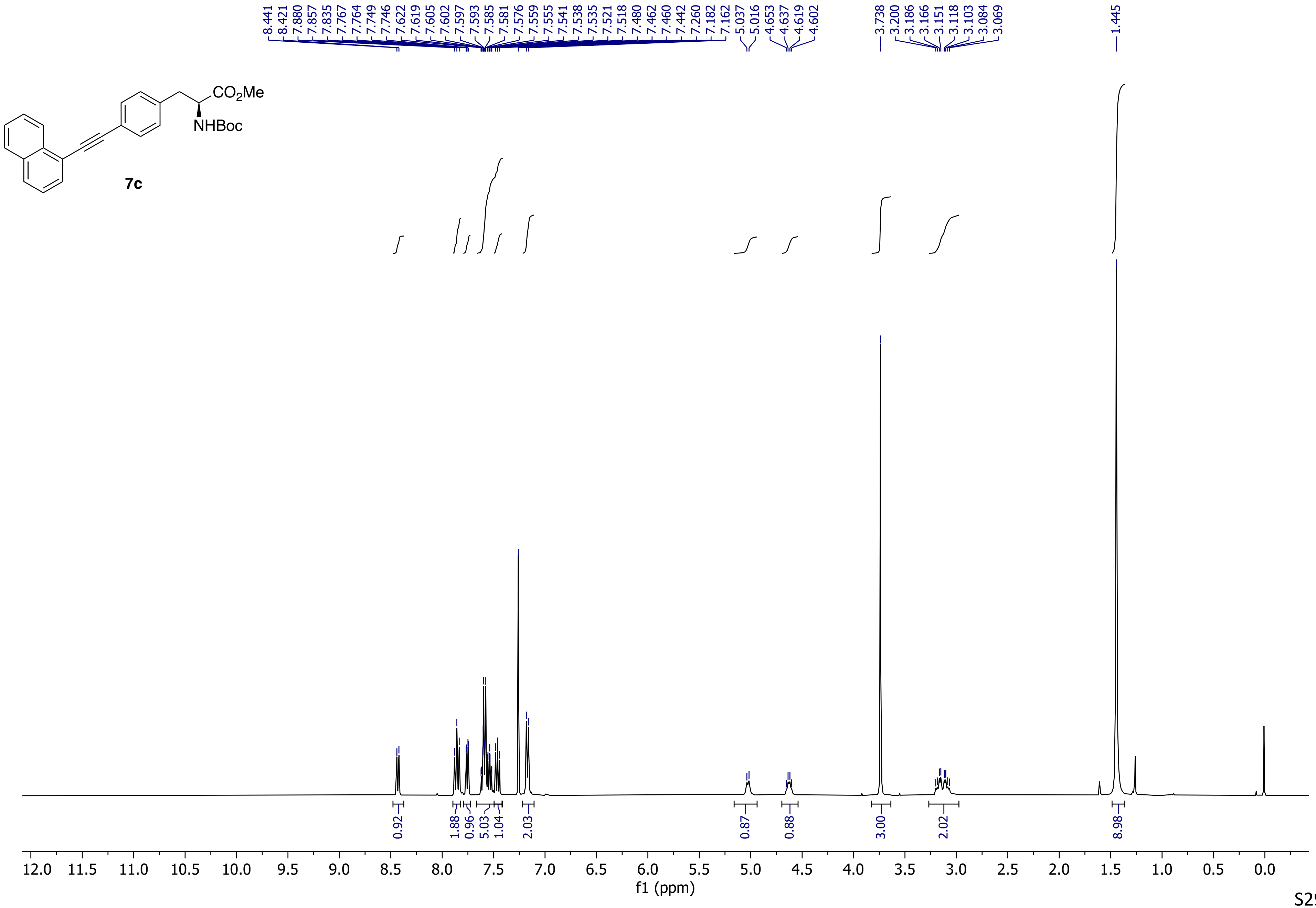

7c

$^{13}C\{^{1}H\}$ NMR (101 MHz, $CDCl_3$)

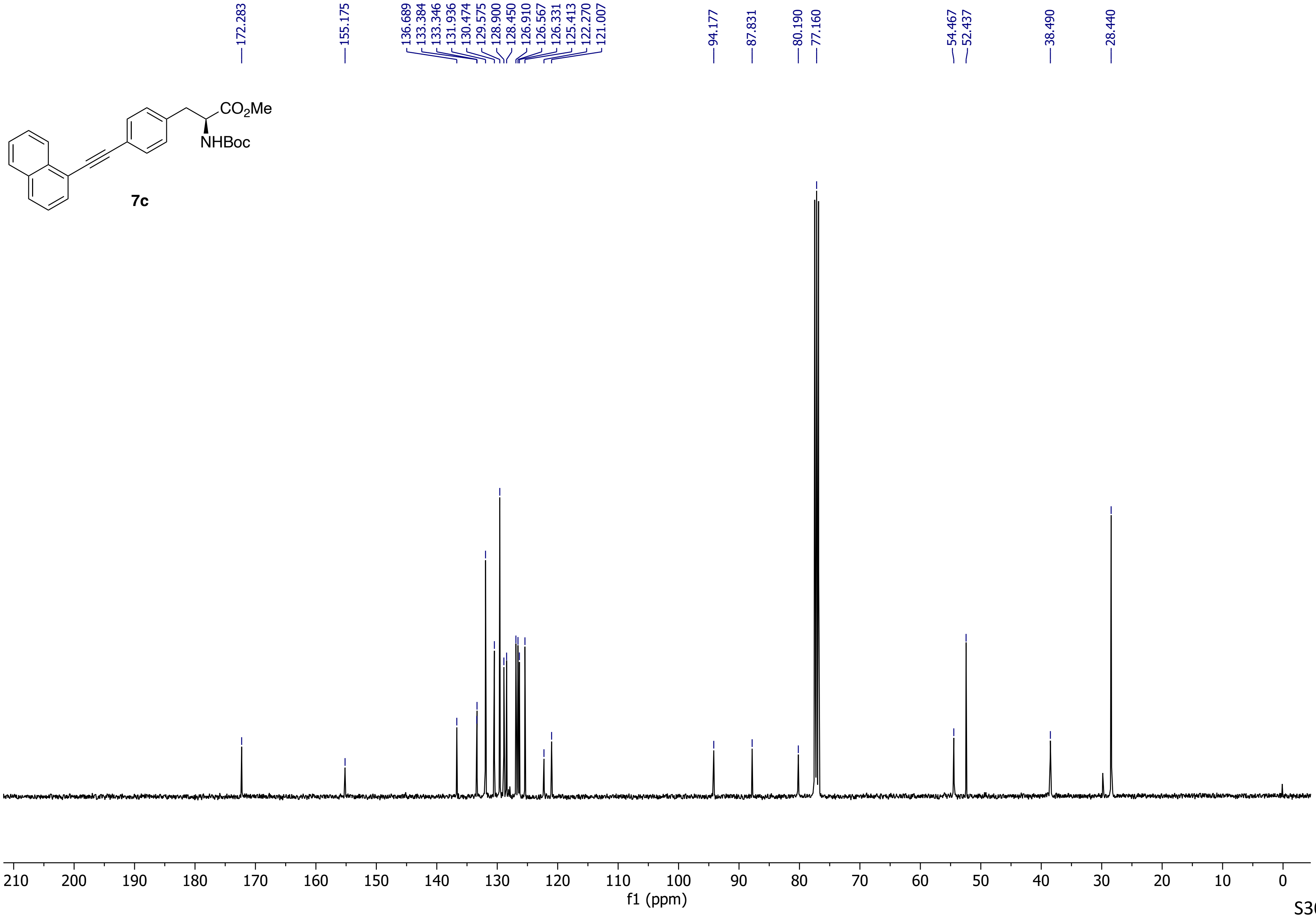

^{1}H NMR (400 MHz, $CDCl_3$)

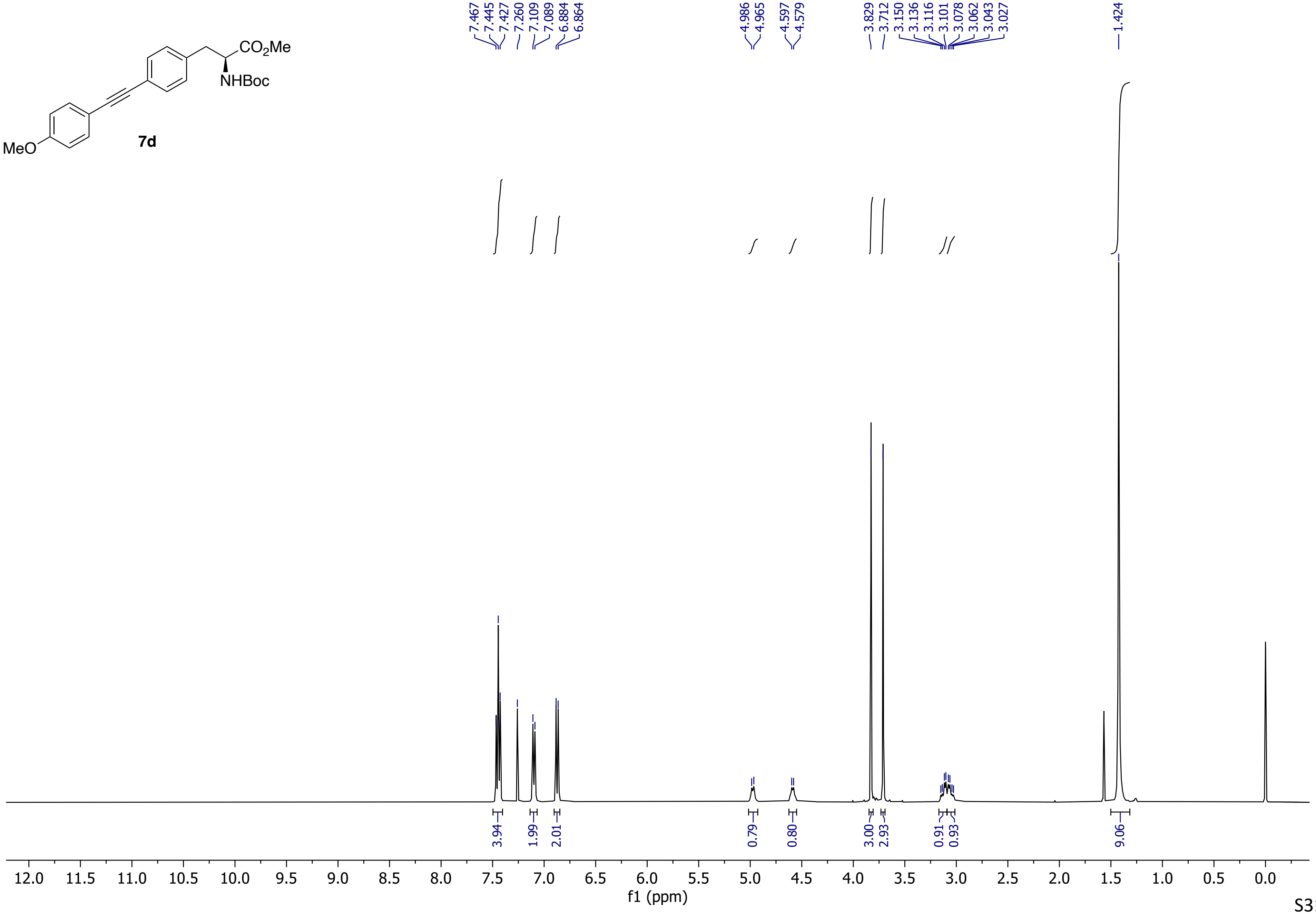

$^{13}C\{^1H\}$ NMR (101 MHz, $CDCl_3$)

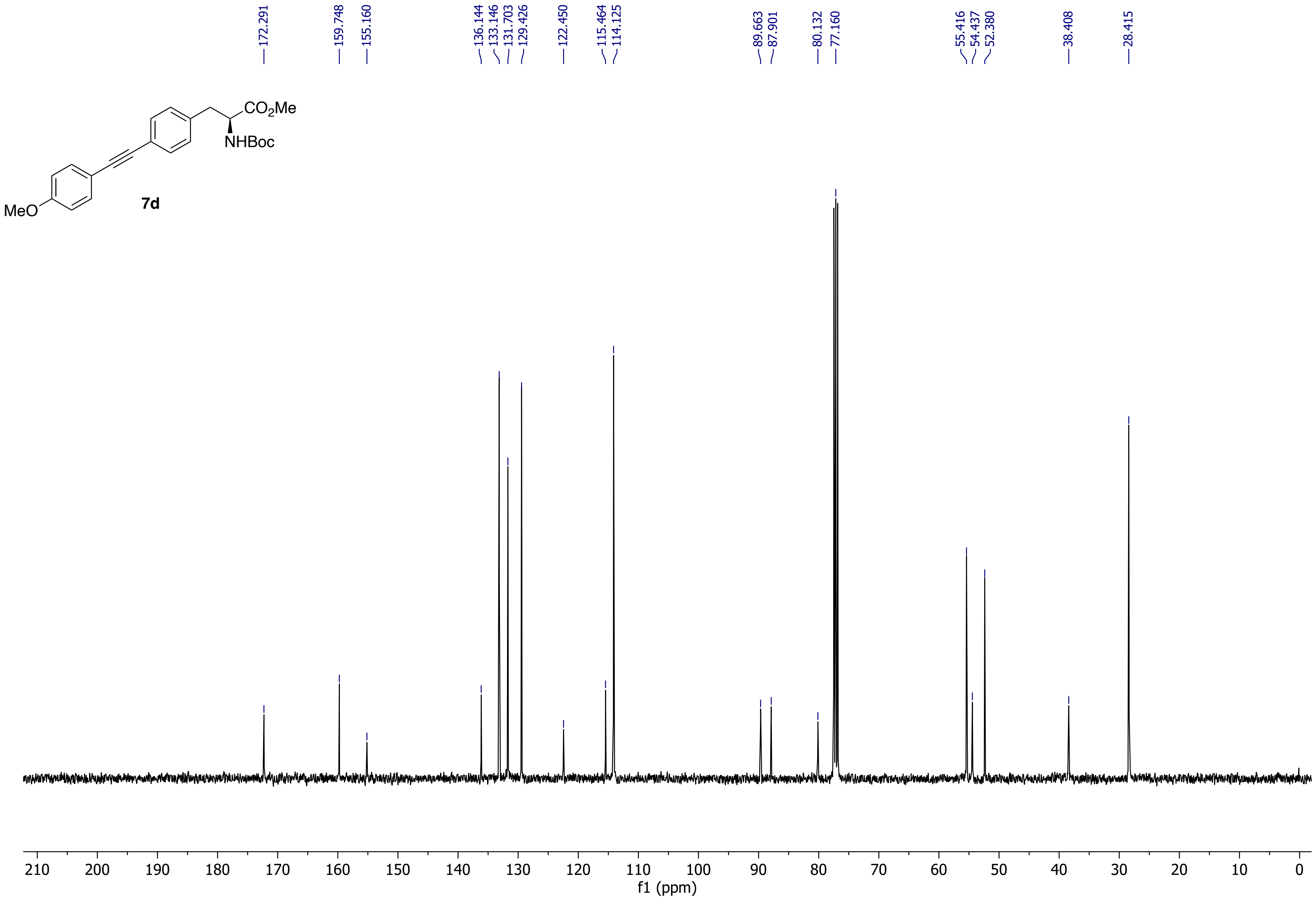

^{1}H NMR (400 MHz, $CDCl_3$)

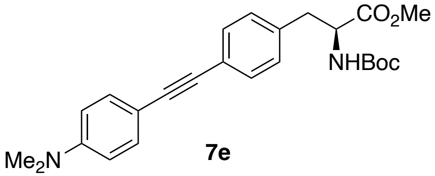

7e

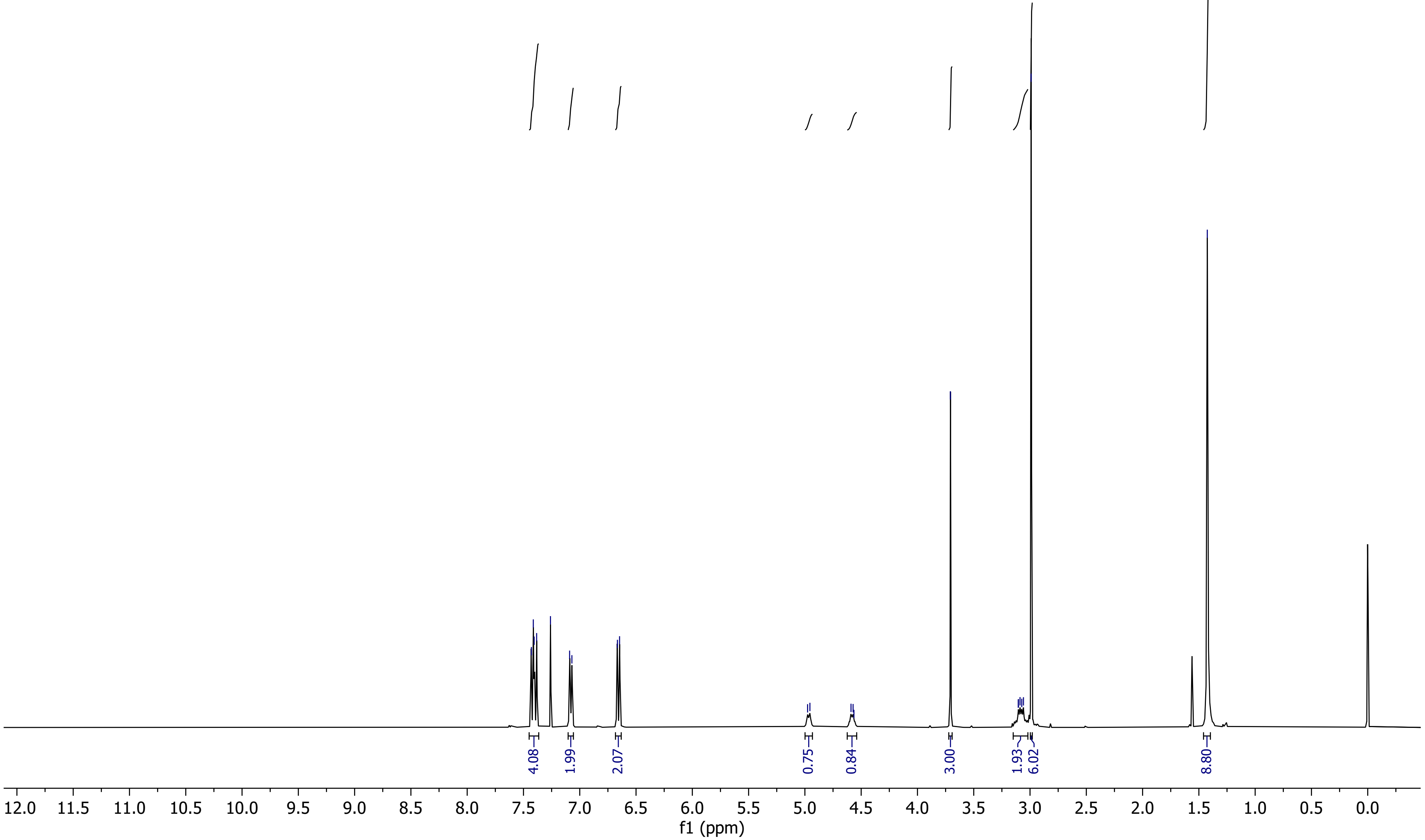

$^{13}C\{^{1}H\}$ NMR (101 MHz, $CDCl_3$)

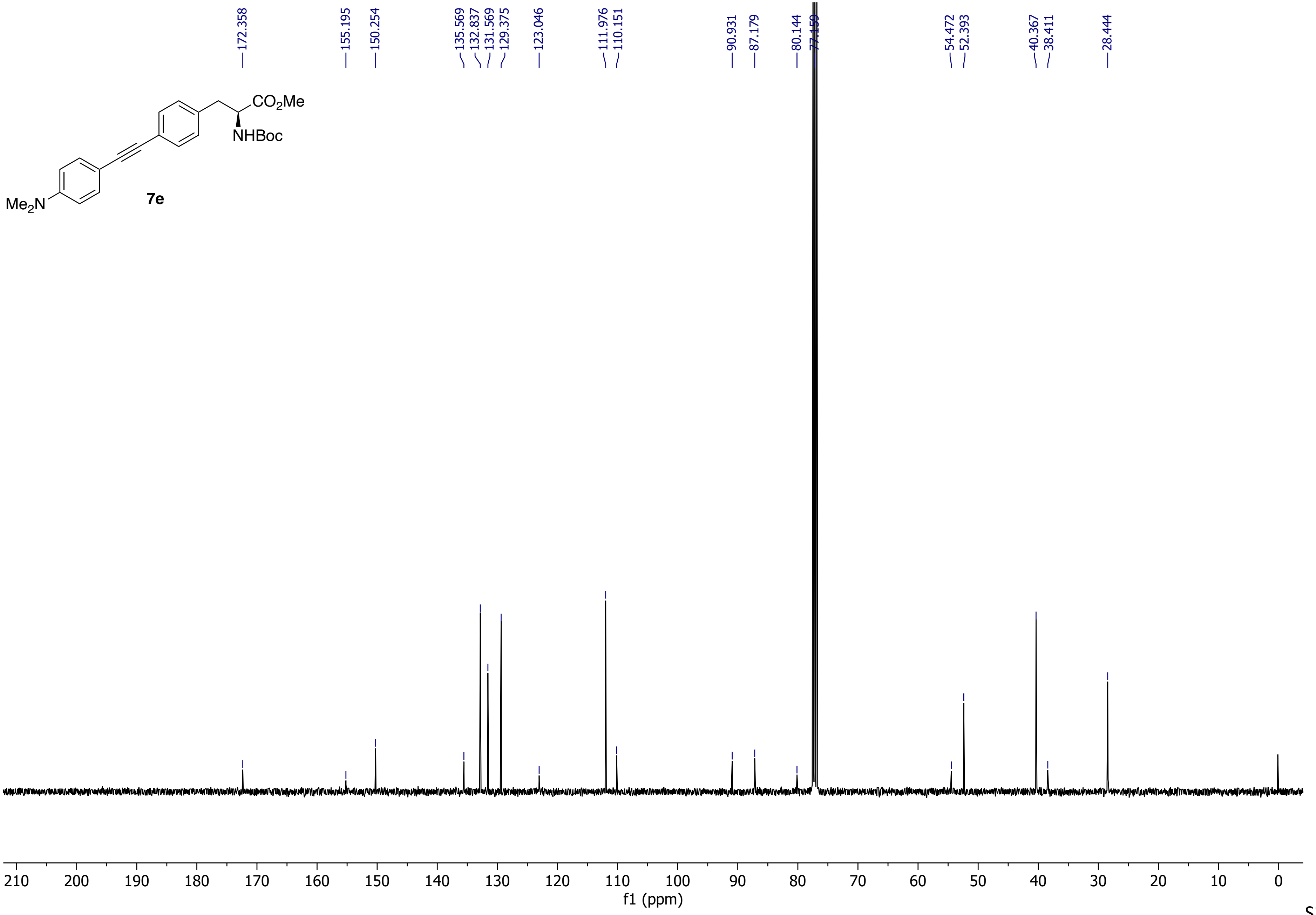

^{1}H NMR (400 MHz, CDCl$_3$)

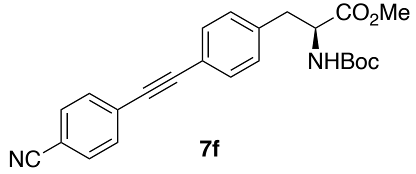

7f

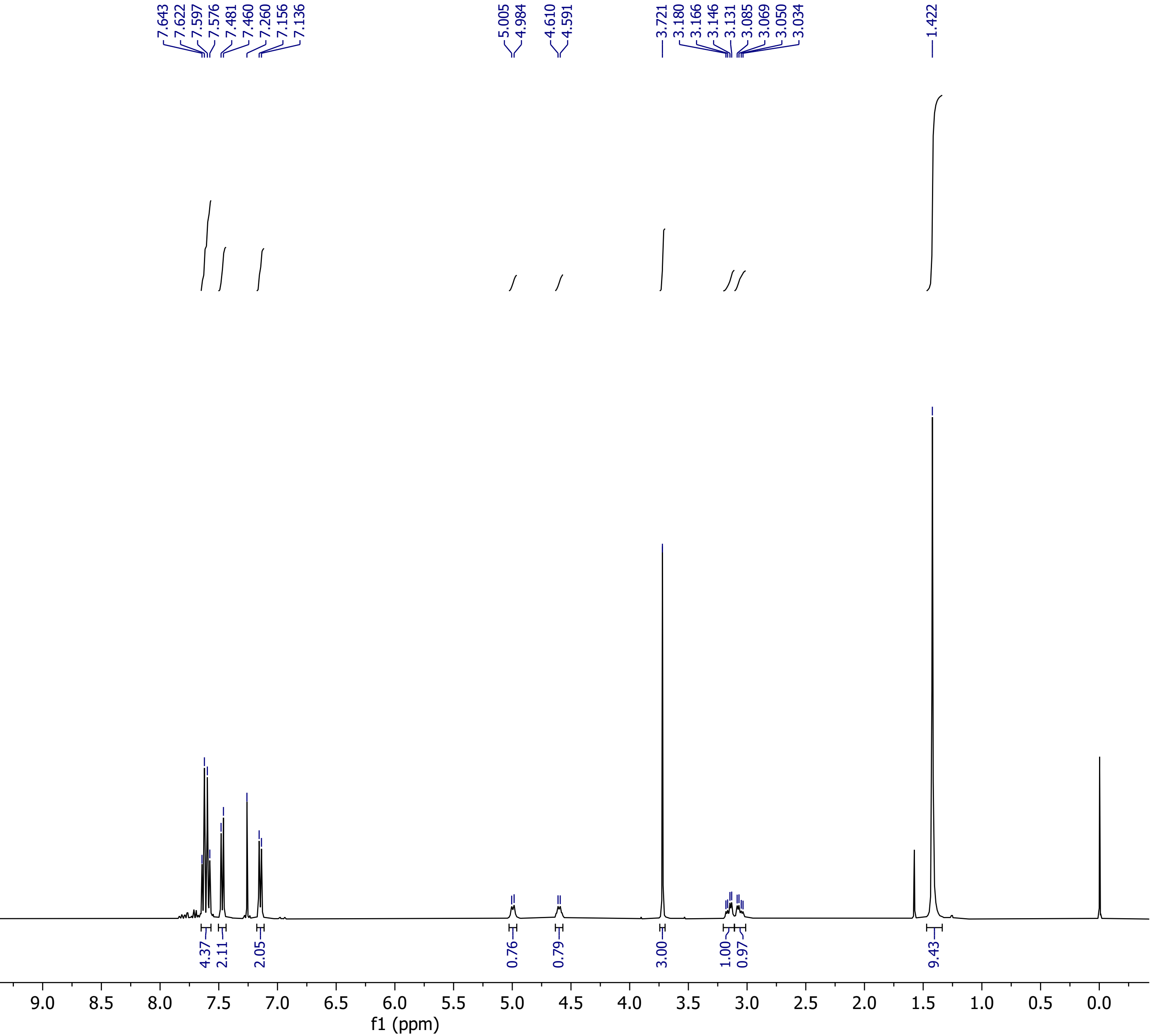

$^{13}C\{^1H\}$ NMR (101 MHz, $CDCl_3$)

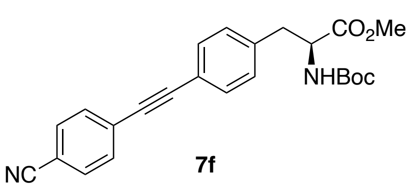

7f

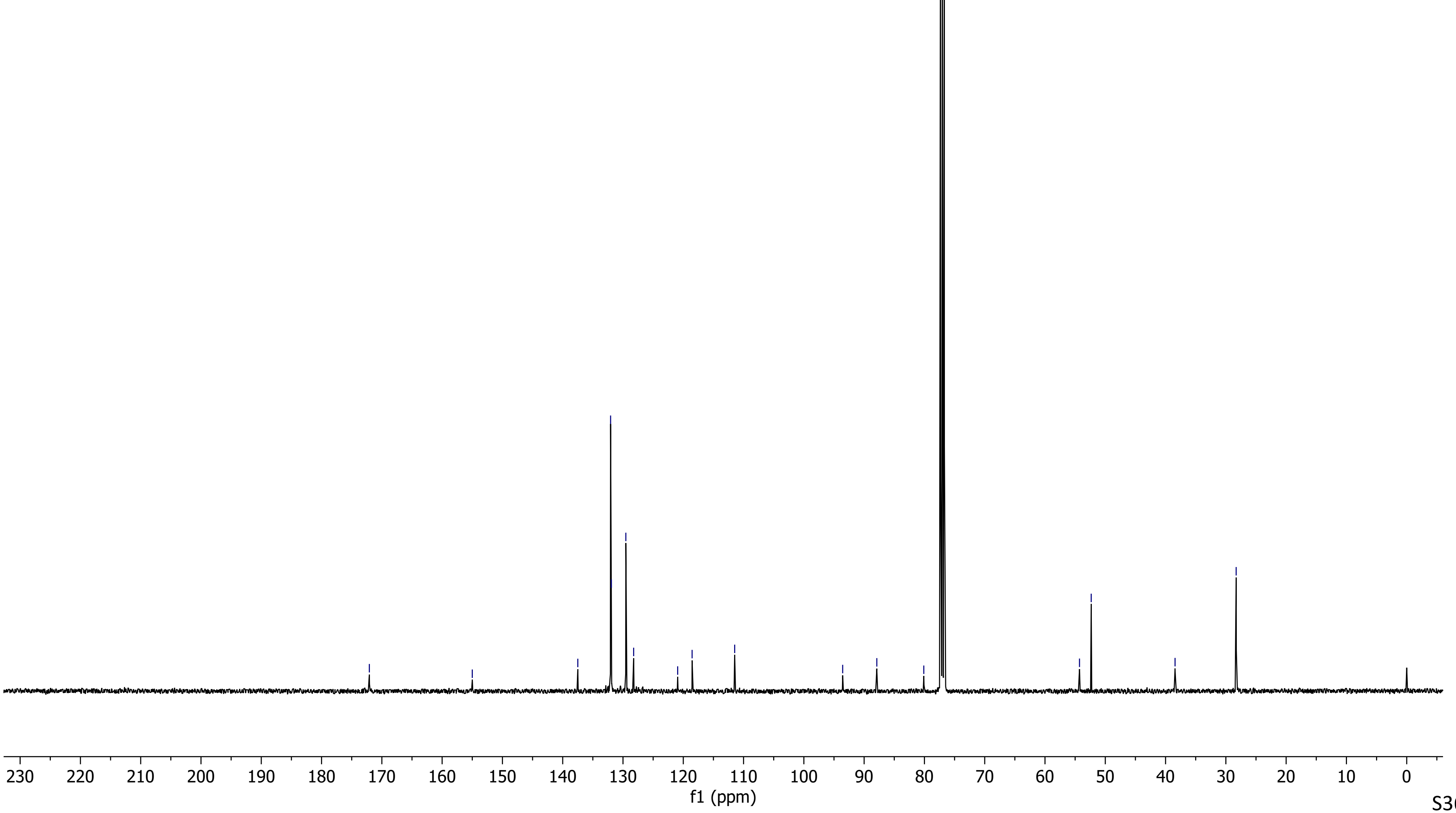

^{1}H NMR (400 MHz, $CDCl_3$)

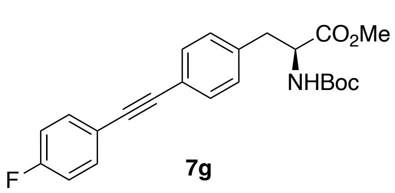

7g

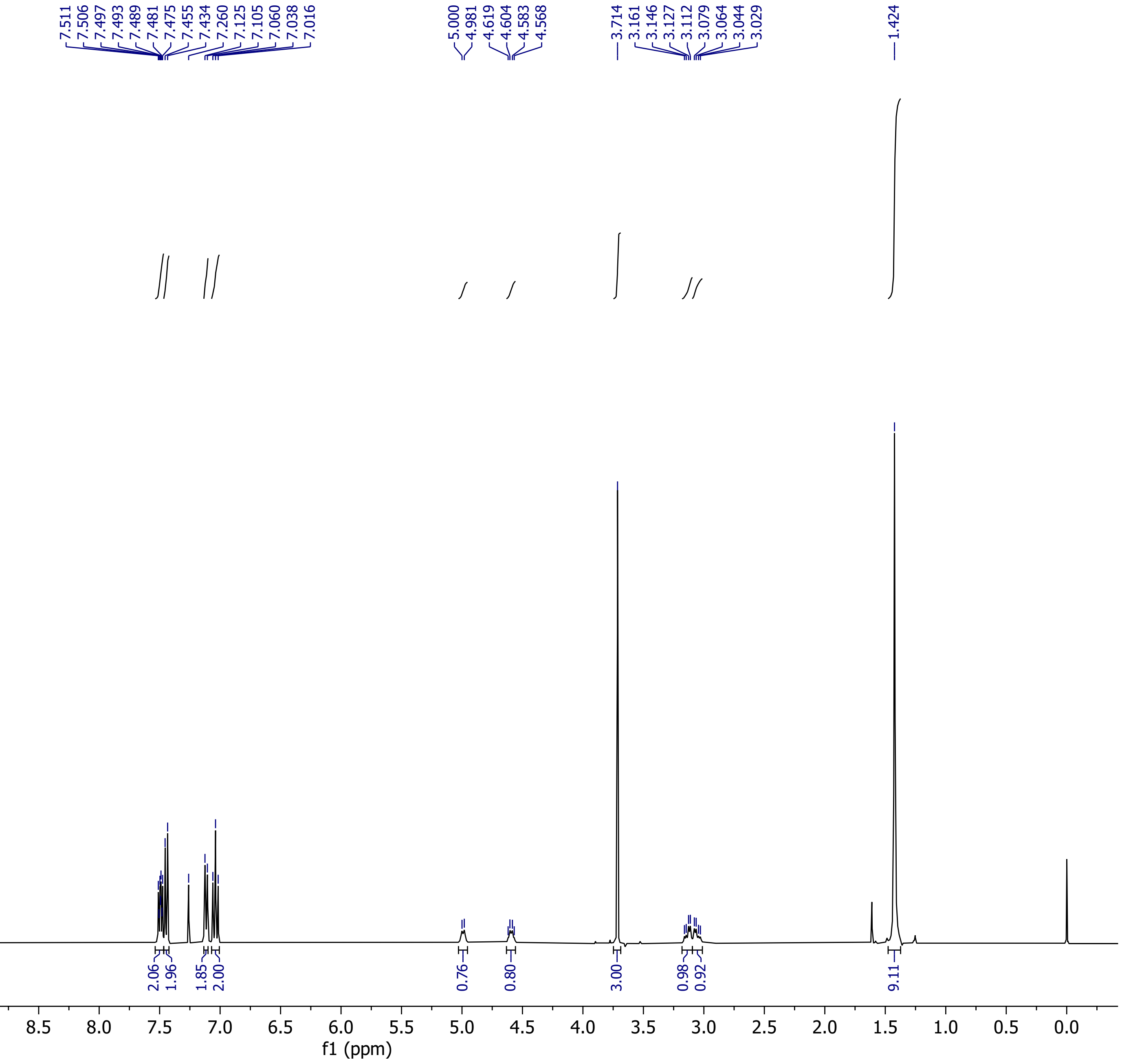

$^{13}C\{^1H\}$ NMR (101 MHz, $CDCl_3$)

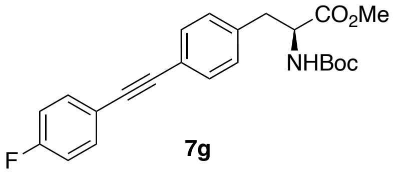

7g

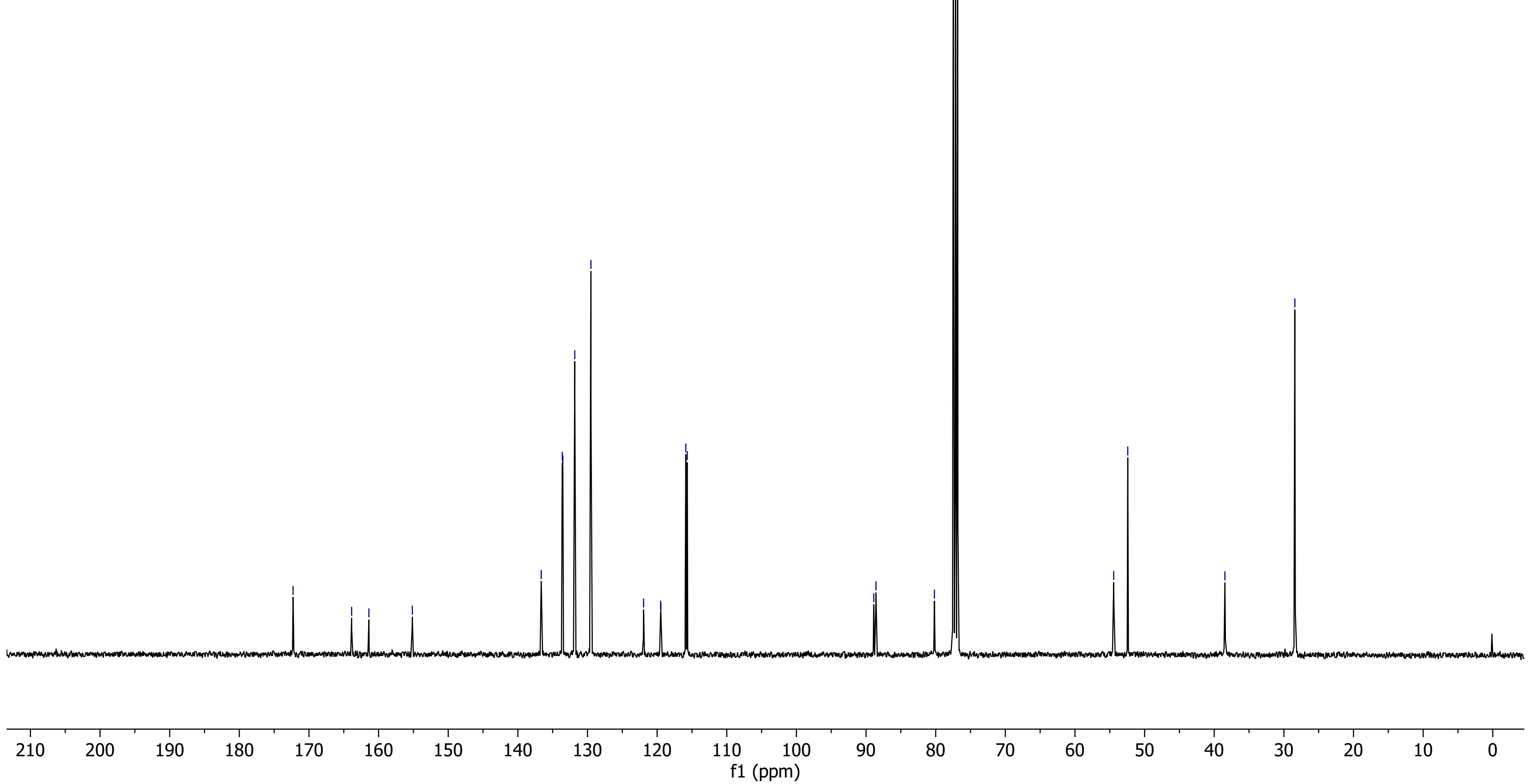

^{1}H NMR (400 MHz, $CDCl_3$)

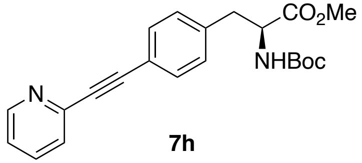

7h

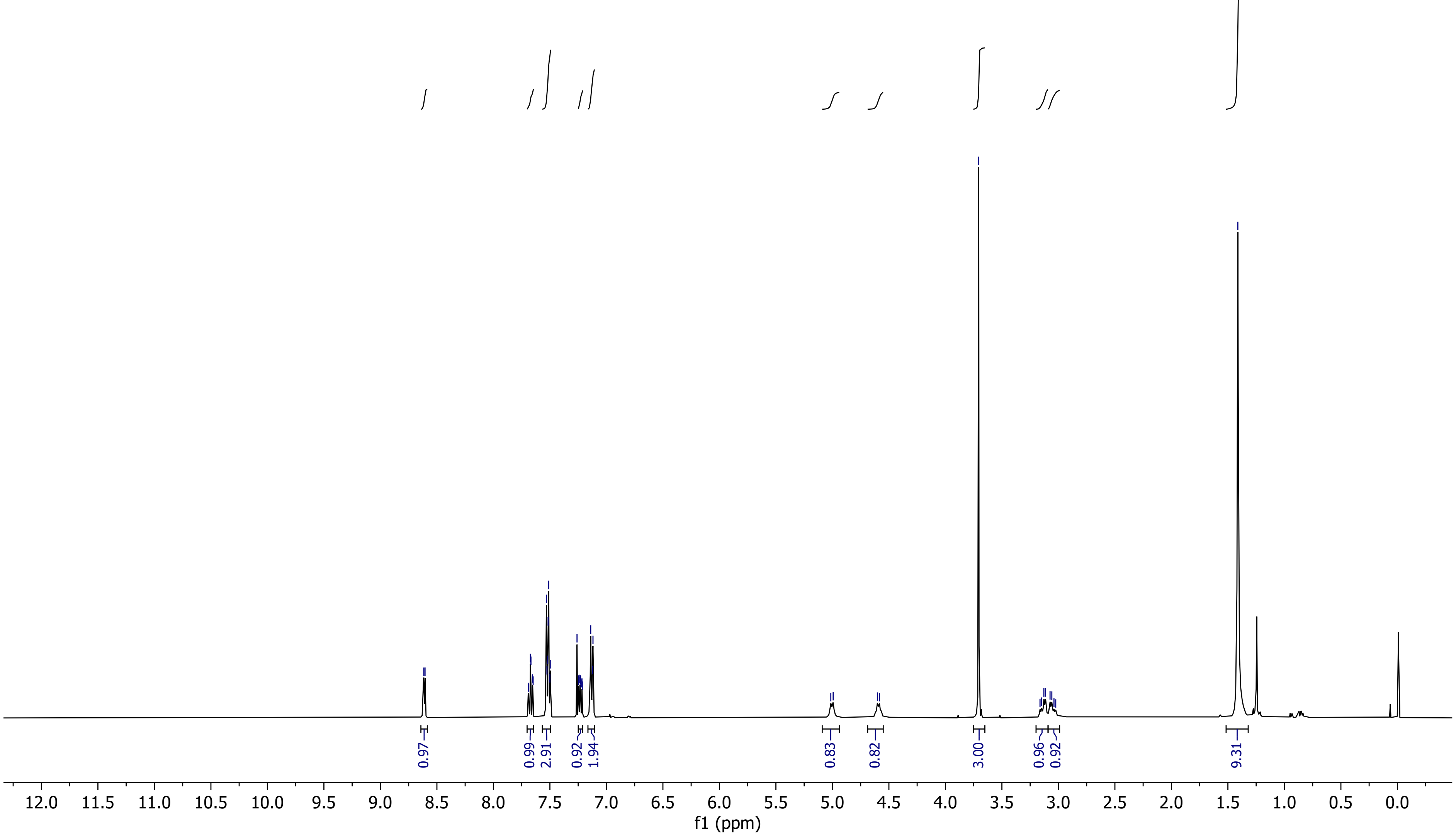

$^{13}C\{^{1}H\}$ NMR (101 MHz, $CDCl_3$)

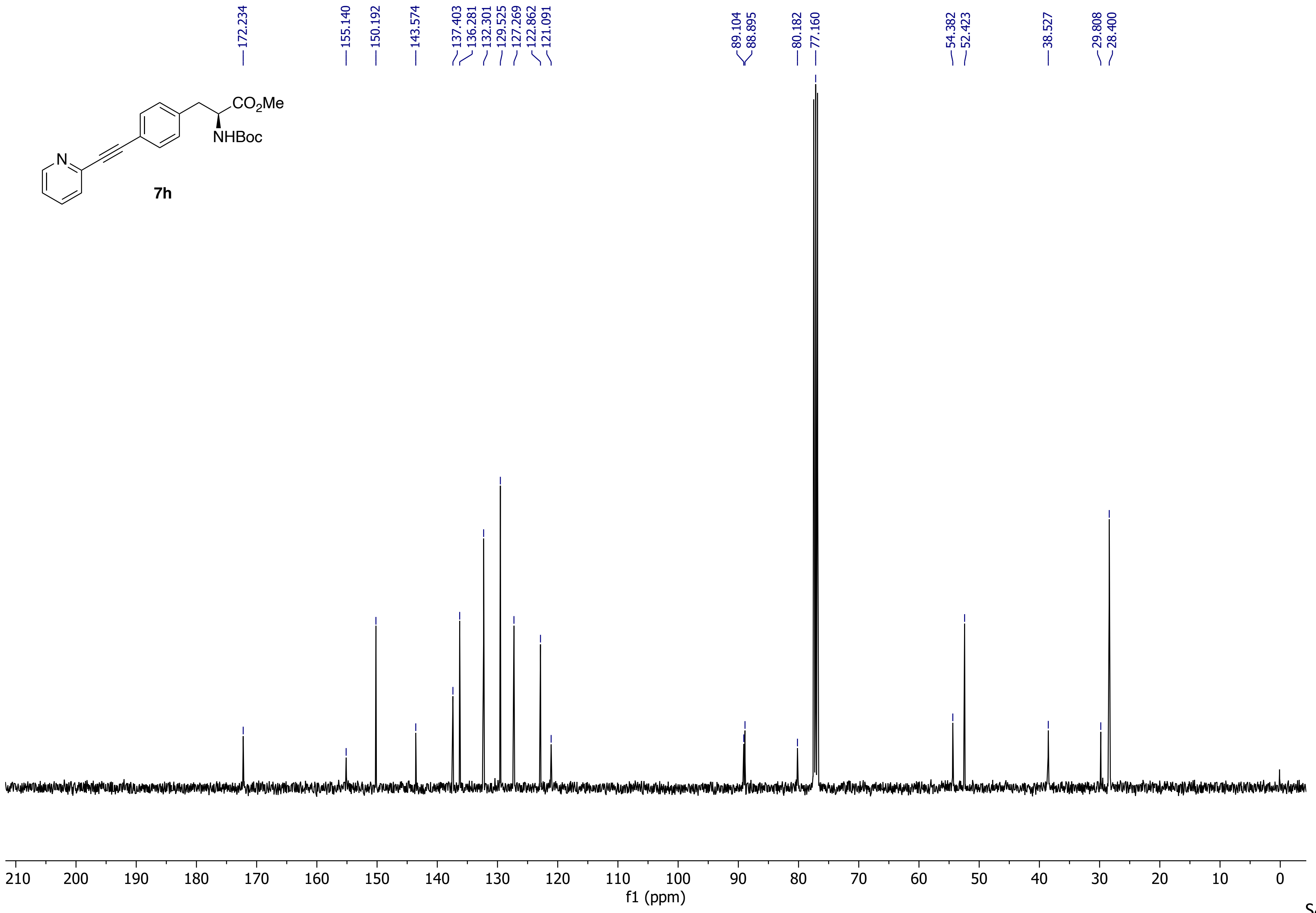

^{1}H NMR (400 MHz, $CDCl_3$)

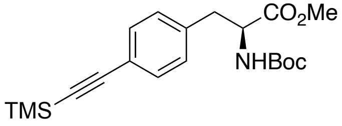

7i

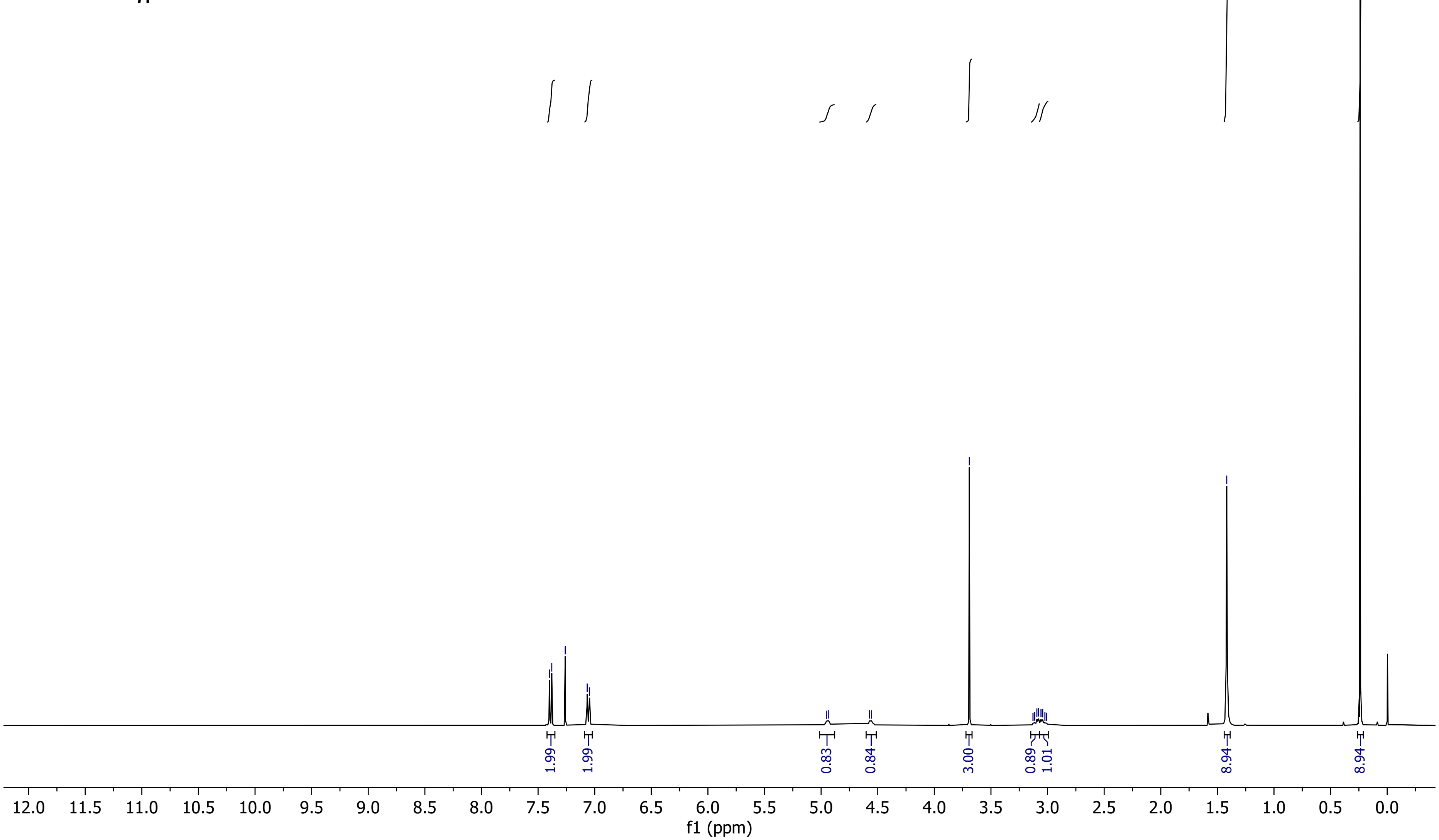

$^{13}C\{^{1}H\}$ NMR (101 MHz, $CDCl_3$)

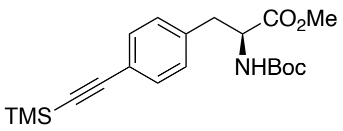

7i

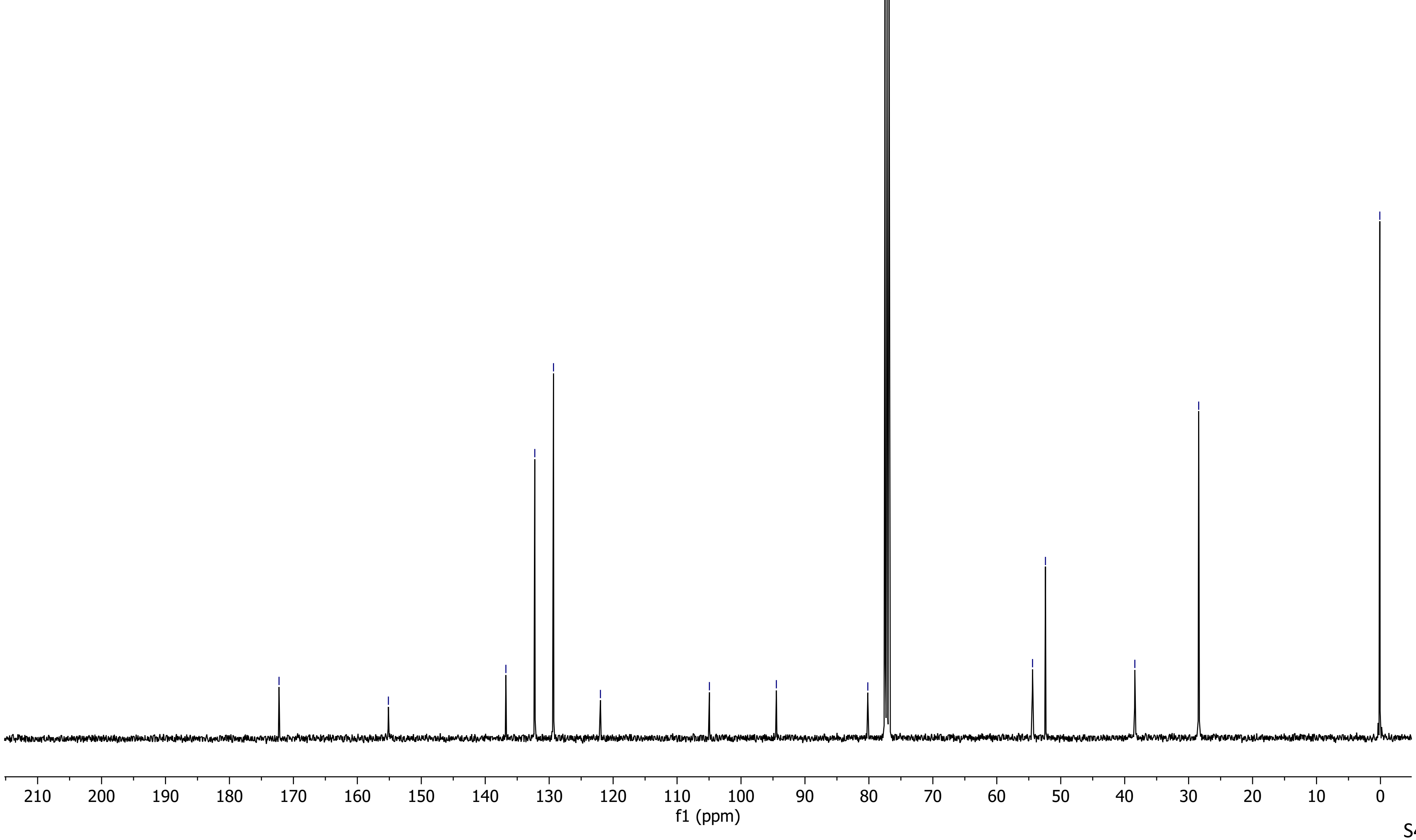

^{1}H NMR (400 MHz, CD_3OD)

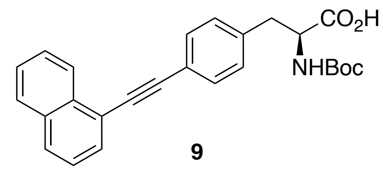

9

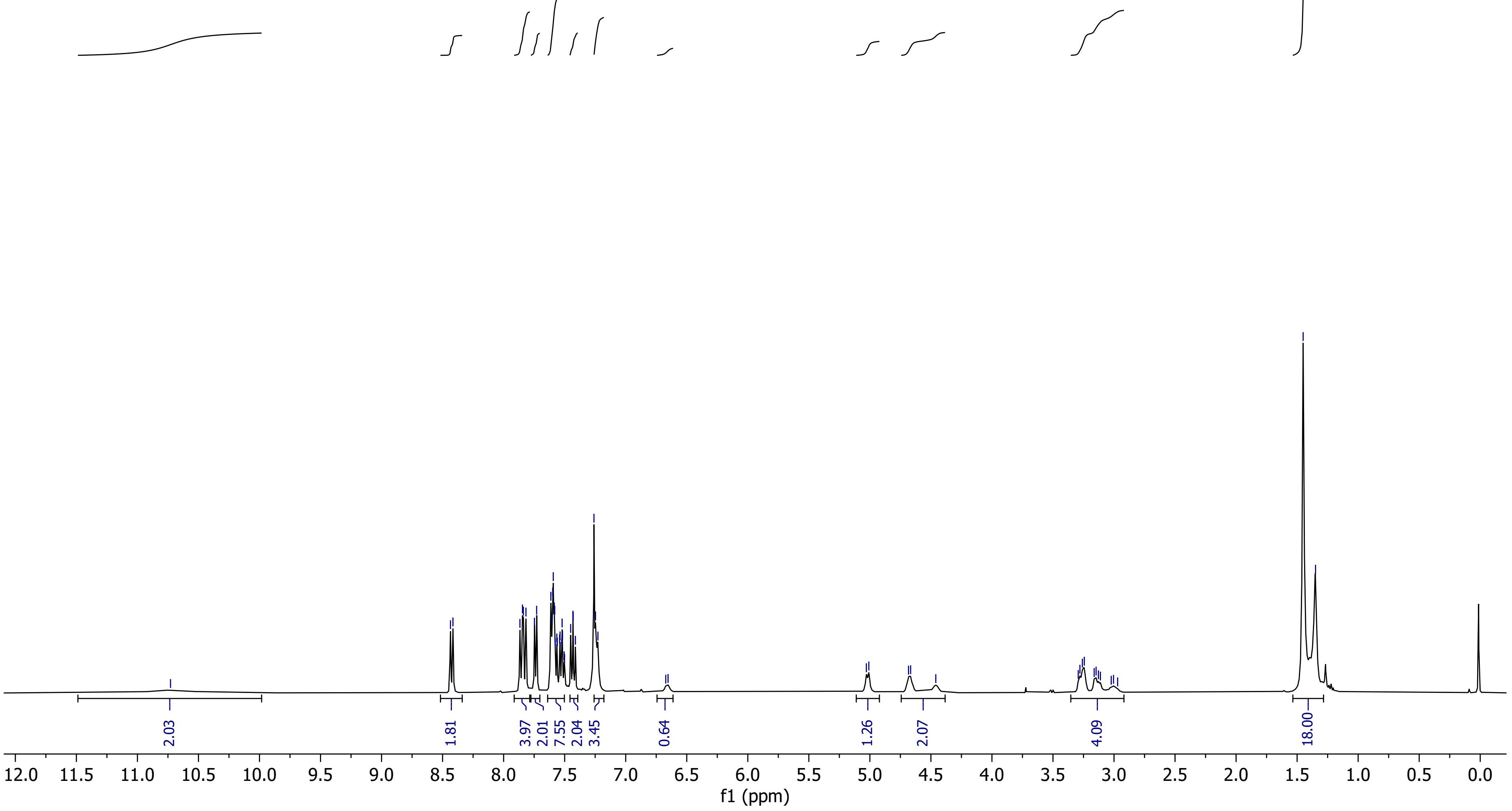

$^{13}C\{^1H\}$ NMR (101 MHz, CD_3OD)

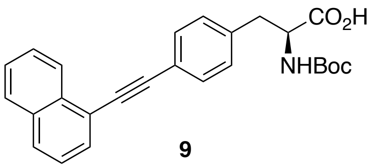

9

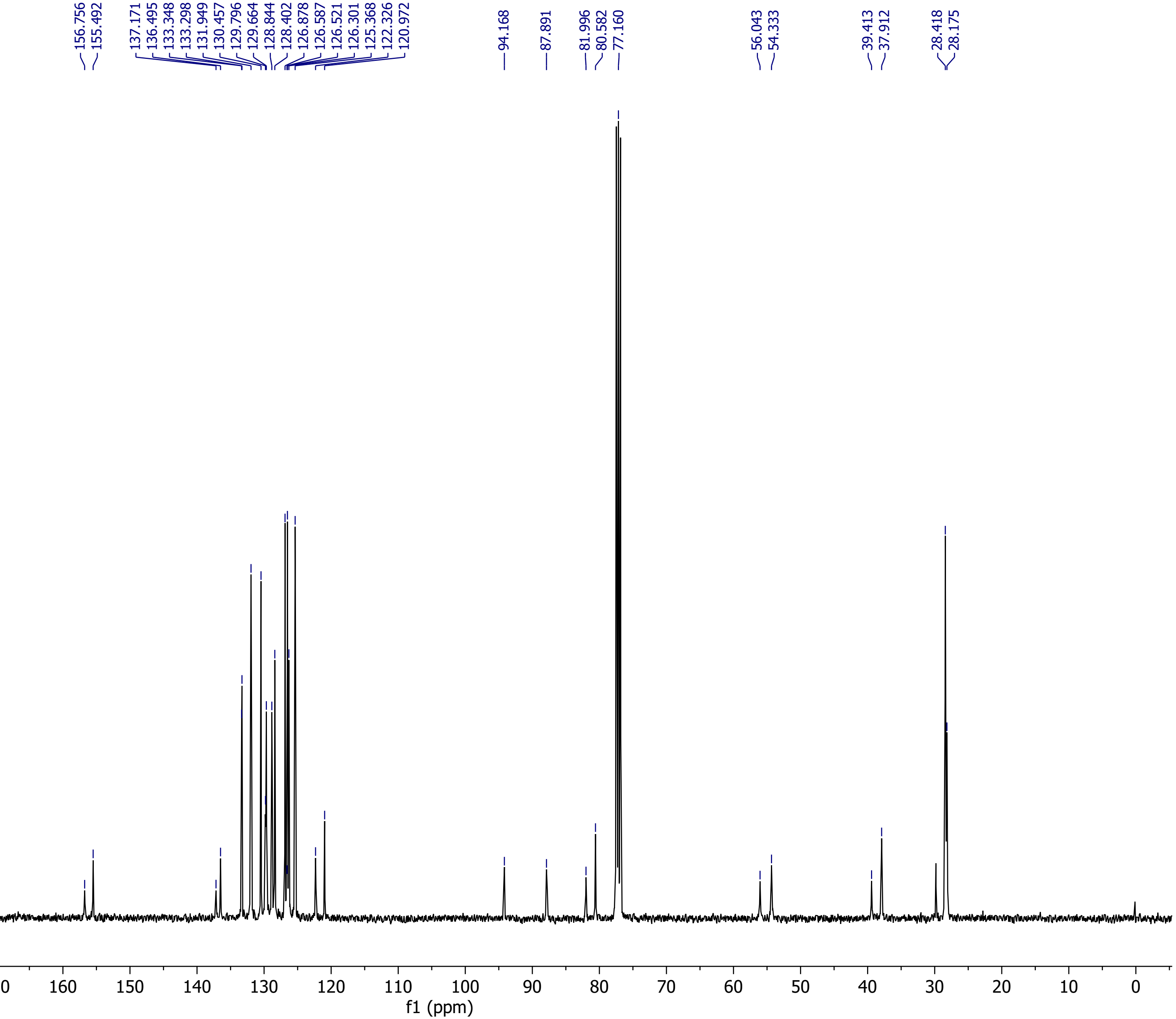

^{1}H NMR (400 MHz, CD_3OD)

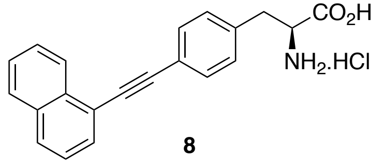

8

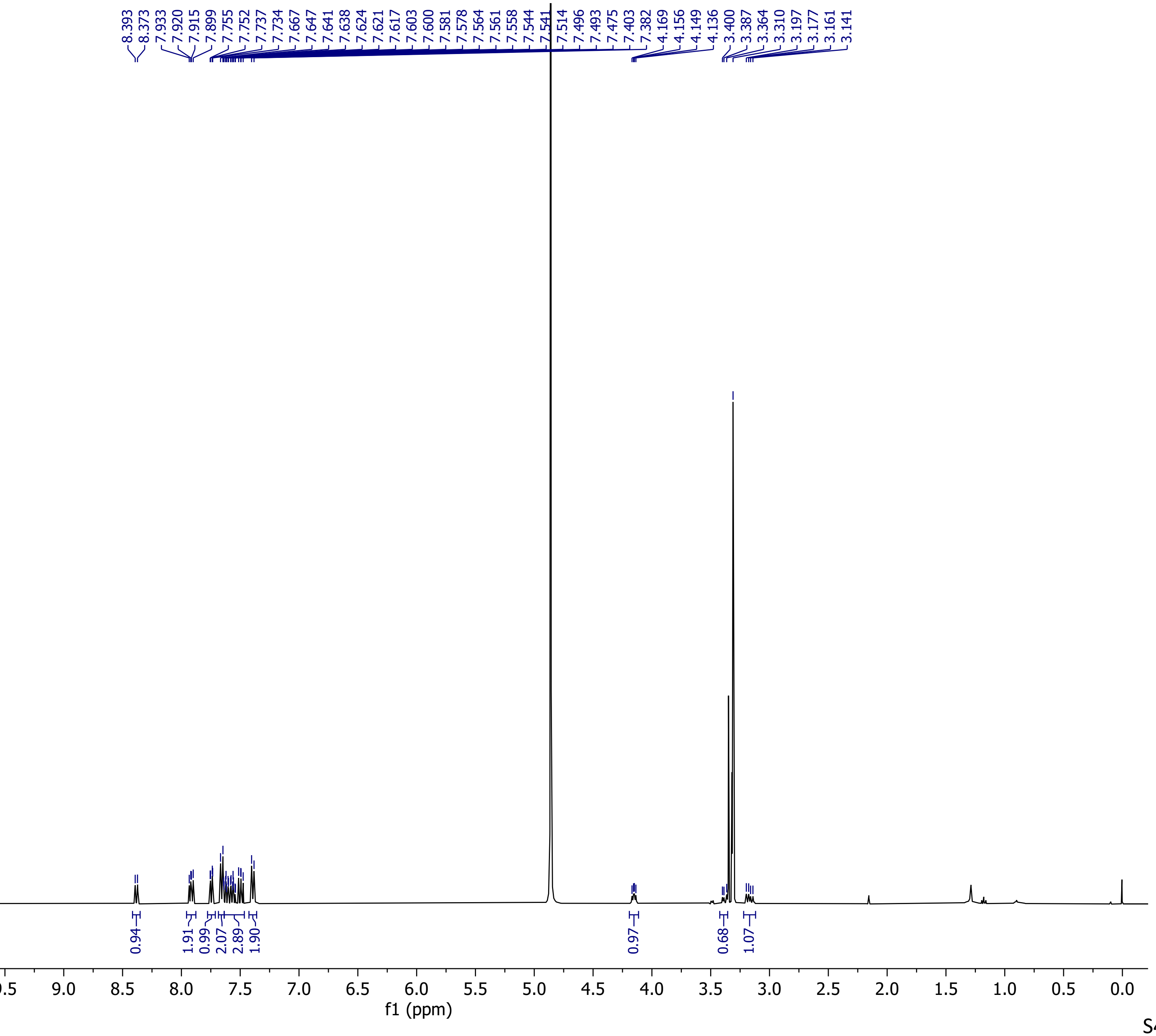

$^{13}C\{^1H\}$ NMR (101 MHz, CD_3OD)

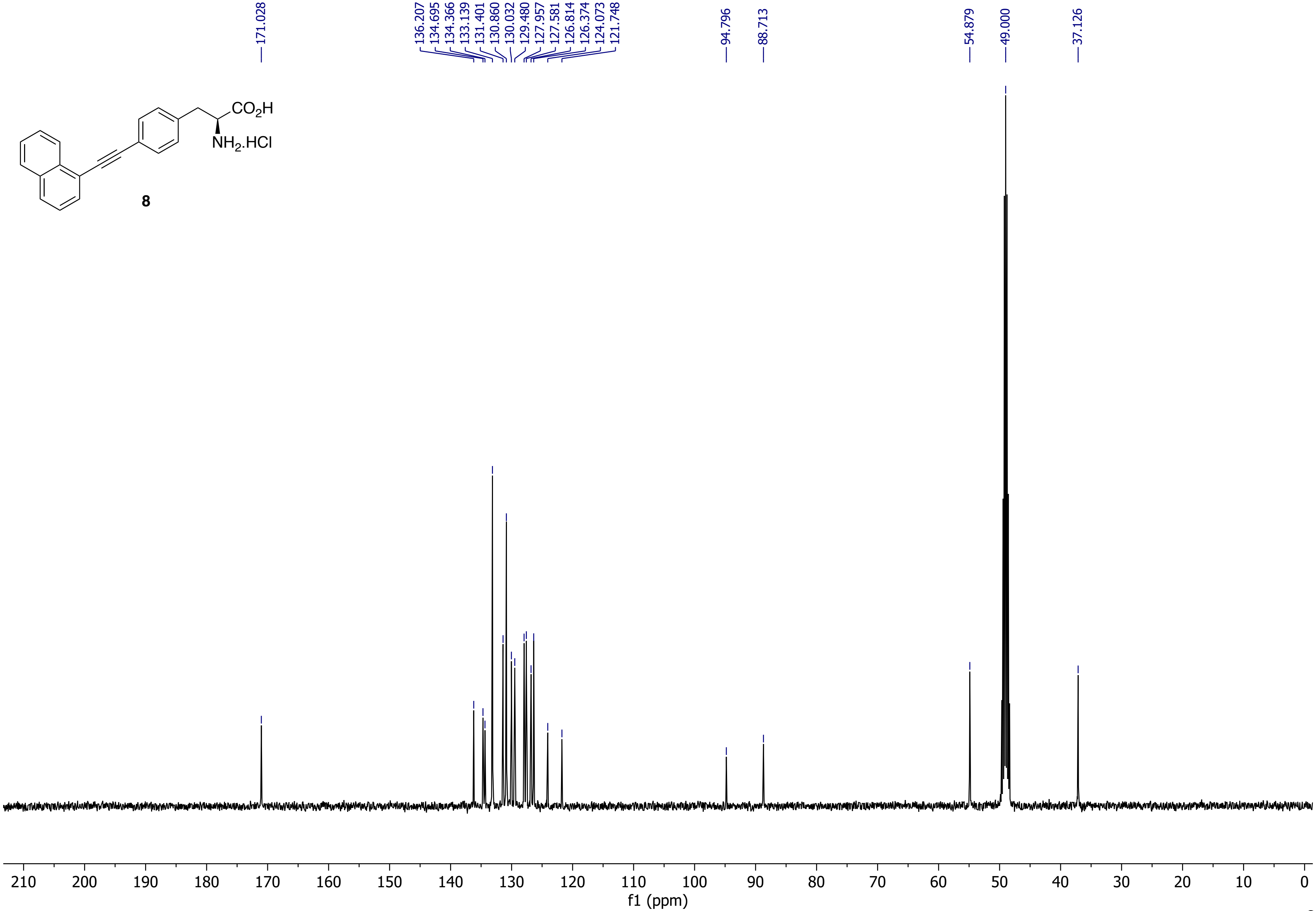

^{1}H NMR (400 MHz, $CDCl_3$)

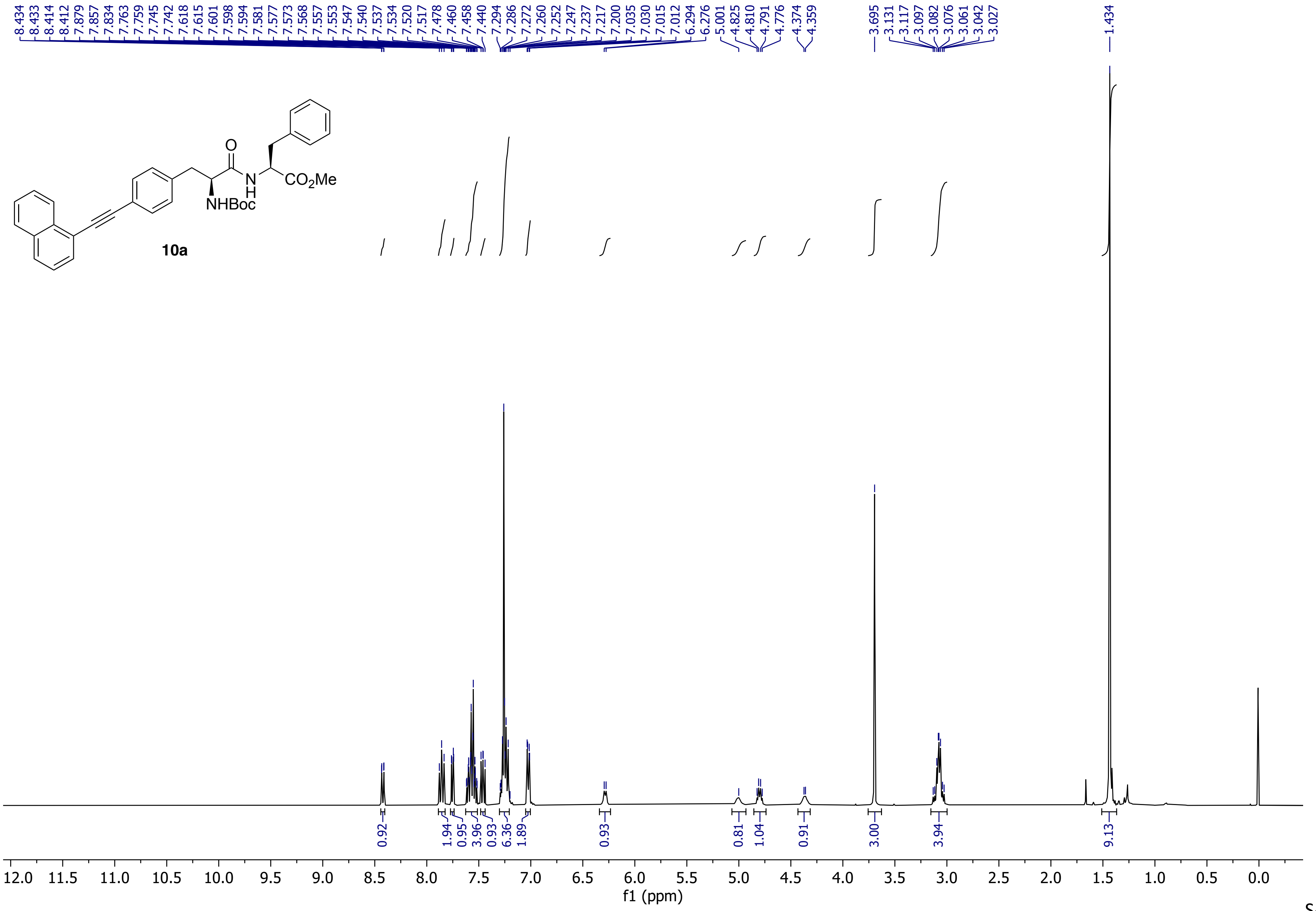

$^{13}C\{^1H\}$ NMR (101 MHz, $CDCl_3$)

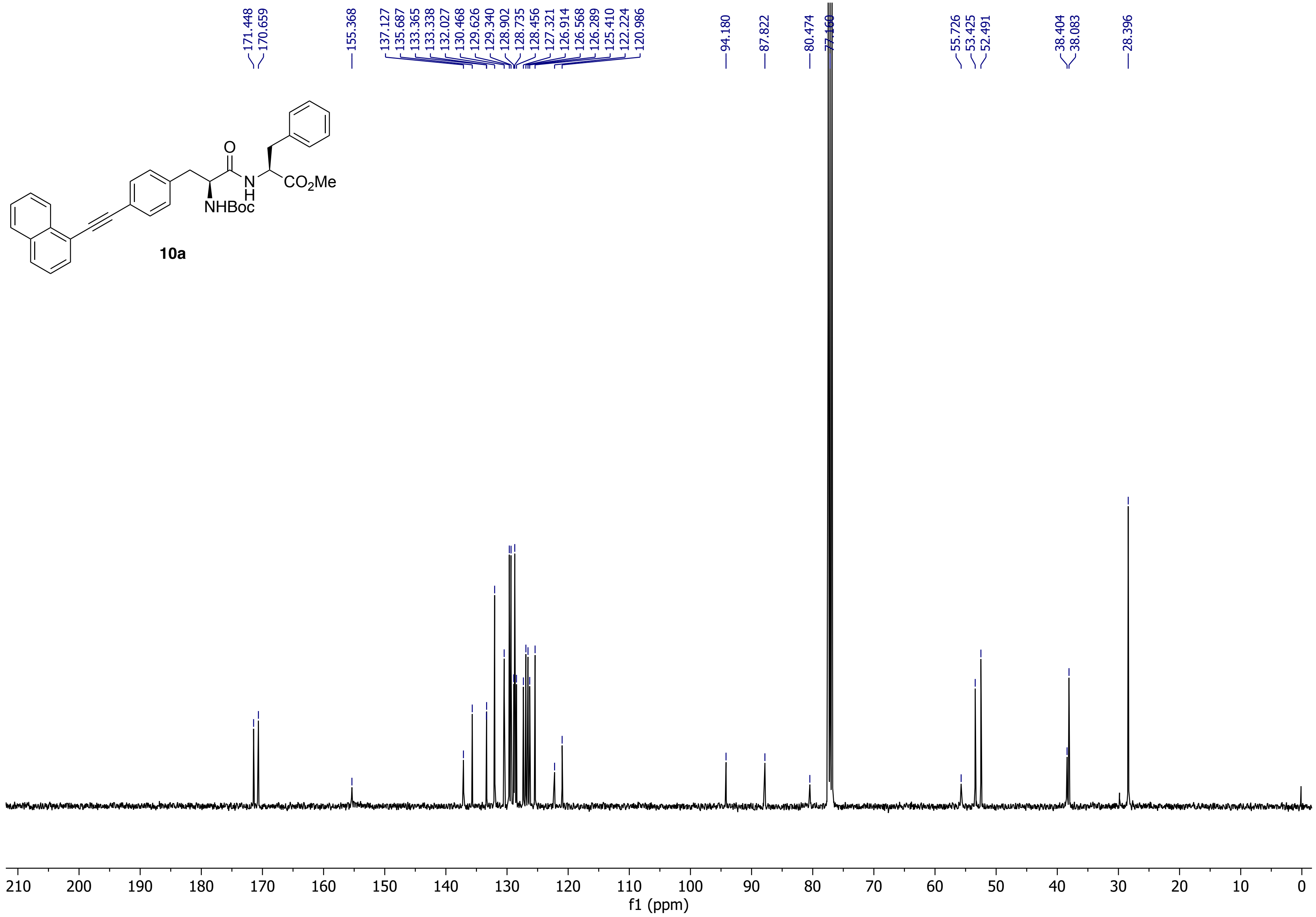

^{1}H NMR (400 MHz, $CDCl_3$)

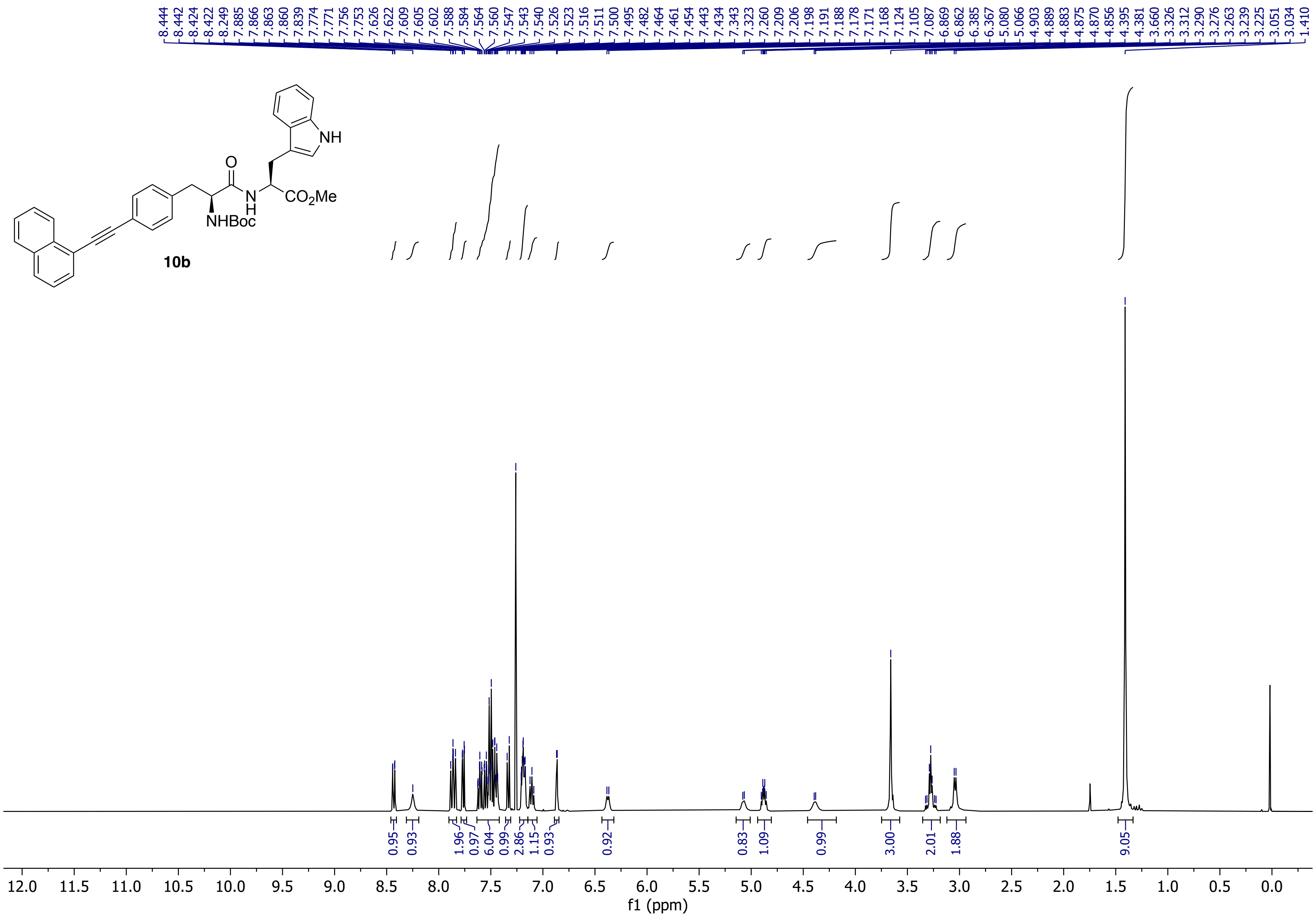

$^{13}C\{^{1}H\}$ NMR (101 MHz, $CDCl_3$)

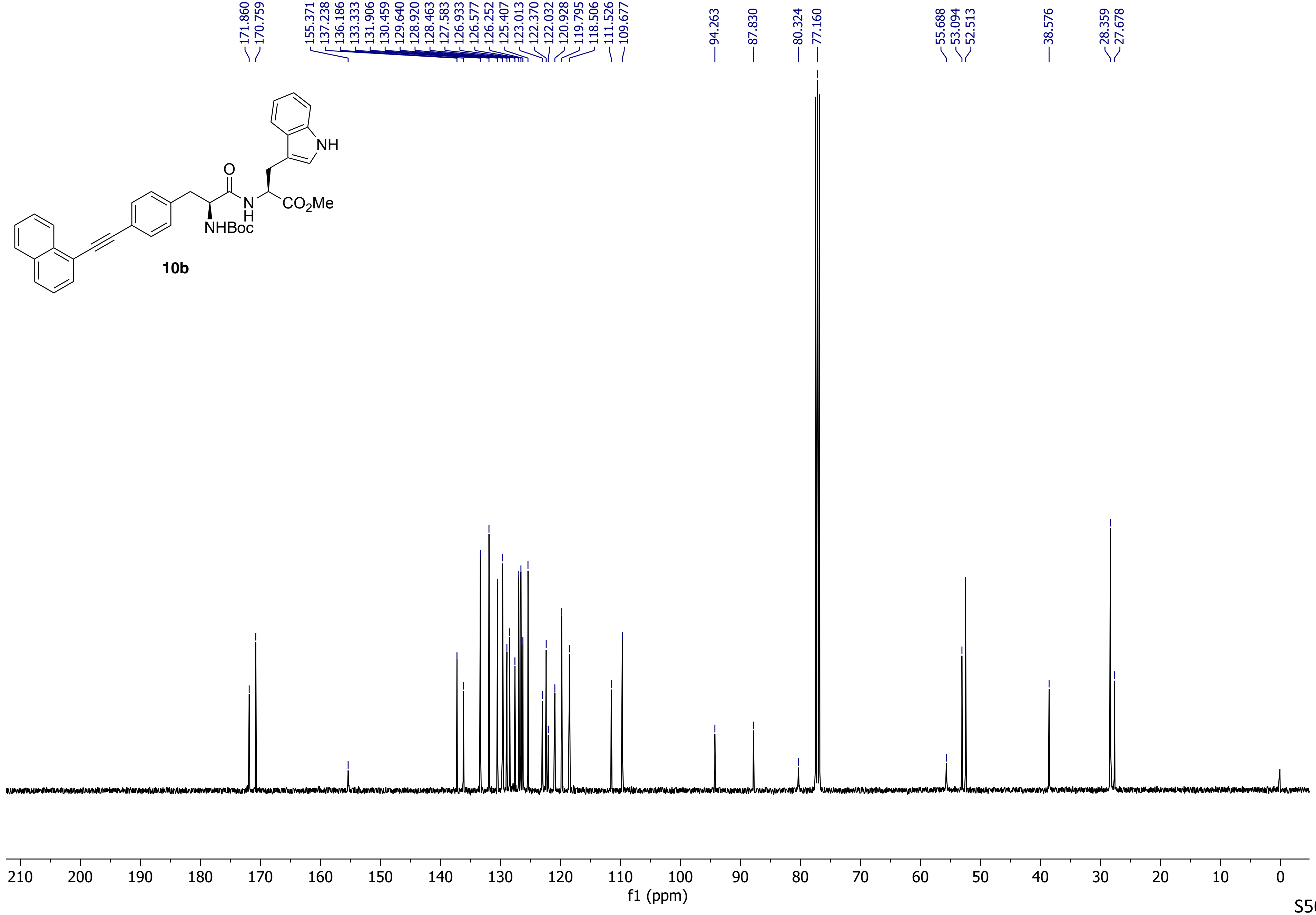

Supplement: Supplementary file 1 [file ol5c02361_si_001.pdf]
